# Ligand- and Additive-Free 2-Position-Selective Trifluoromethylation of Heteroarenes Under Ambient Conditions

**DOI:** 10.3389/fchem.2019.00613

**Published:** 2019-09-06

**Authors:** Xiaolin Shi, Xiaowei Li, Xiangqian Li, Dayong Shi

**Affiliations:** ^1^Key Laboratory of Experimental Marine Biology, Institute of Oceanology, Chinese Academy of Sciences, Qingdao, China; ^2^Laboratory for Marine Drugs and Bioproducts of Qingdao National Laboratory for Marine Science and Technology, Qingdao, China; ^3^University of Chinese Academy of Sciences, Beijing, China; ^4^State Key Laboratory of Microbial Technology, Shandong University, Qingdao, China

**Keywords:** C-H functionalization, trifluoromethylation, indoles, copper, radical reaction

## Abstract

A highly selective copper-catalyzed trifluoromethylation of indoles is reported with the assistance of a removable directing group. This protocol provides an easy and rapid method to various 2-position-selective trifluoromethylated heteroarenes including indoles, pyrroles, benzofuran, and acetanilide. What is more, the reaction takes place at ambient conditions without any external ligand or additive.

## Introduction

**Scheme 1:**
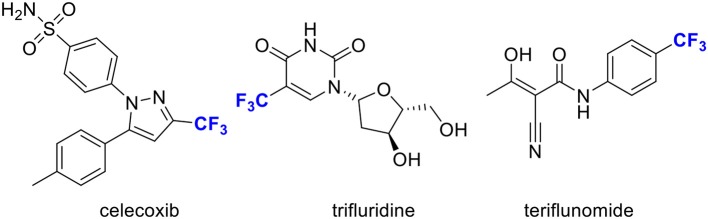
Several examples of pharmaceuticals with a trifluoromethylation group.

The introduction of trifluoromethyl (CF_3_) groups into heteroarenes enjoys a privileged role in medicinal chemistry, since it can substantially alter their properties, such as metabolic stability, lipophilicity and ability to penetrate the blood-brain barrier (Shimizu and Hiyama, 2005; Schlosser, 2006; Hagmann, 2008; Boechat and Bastos, 2010; Nie et al., 2011; Wang et al., 2014; Gouverneur and Seppelt, 2015). It has a great potential in the development of new pharmaceutical chemicals (Scheme 1). Thus, the trifluoromethylation of heteroarenes has received tremendous attentions (Sato et al., 2010; Furuya et al., 2011; Tomashenko and Grushin, 2011; Liu et al., 2013; Le et al., 2018). On the other hand, indoles represent ubiquitous structural motifs found in biologically active natural products and pharmaceutical compounds (Lee et al., 2015; Chripkova et al., 2016; Sravanthi and Manju, 2016; Goyal et al., 2018; Kaur et al., 2019). In this regard, direct trifluoromethylation of indoles offers an attractive alternative to the workers in the field of medicinal chemistry and biochemistry.

**Scheme 2:**
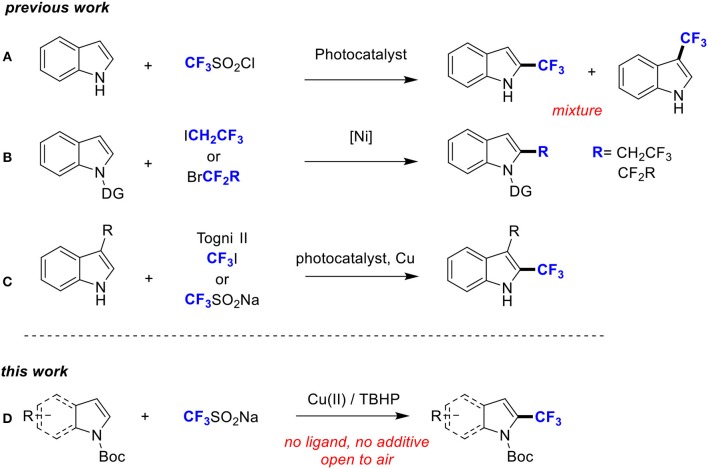
Different approaches to C2 functionalized indole derivatives **(A–D)**.

However, direct trifluoromethylation at the C2-position of indoles under radical trifluoromethylation conditions is quite difficult because of the lack of reaction selectivity at the C2/C3-position and the high reactivity of the CF_3_ radical (Scheme 2A; Nagib and MacMillan, 2011). Recently, directing group (DG) has emerged as a powerful tool to achieve regioselective C2-H functionalization of indoles (Nishino et al., 2012; Zhou et al., 2013; Yu et al., 2014). For example, Shi group and Punji group, respectively, achieved trifluoroethylation and difluoroalkylation of indoles at C2 position by introducing a directing group at the *N*-center of indoles (Scheme 2B; Yan et al., 2017; Soni et al., 2018). Also, Sodeoka group, Cho group, and Shi group accomplished trifluoromethylation of indoles at the C2 position with Togni's reagent, CF_3_I and CF_3_SO_2_Na respectively. However, a substituent at C3 was identified as a crucial factor for the selective trifluoromethylation at C2 (Scheme 2C; Shimizu et al., 2010; Iqbal et al., 2012; Shi et al., 2018).

In this context, we envisioned that with the aid of a readily removable *N*-protecting group, trifluoromethyl group can be introduced to C2 position, which is complementary to the previous work. Herein, we report a copper-catalyzed C2-H trifluoromethylation of *N*-Boc (*t*-butyloxy) indoles with CF_3_SO_2_Na under ambient conditions in the absence of any external ligand or additive (Scheme 2D). Notably, the key to our success is the installation of a suitable Boc director on the indole nitrogen atom.

## Results and Discussion

To begin, we chose *N*-Boc indole (**1a**) as model substrate. To our delight, when the reaction mixture of *N*-Boc indole (**1a**, 0.50 mmol), **2** (1.5 mmol), TBHP (*t*-butyl hydroperoxide, 70% solution in H_2_O, 2.5 mmol) and CuSO_4_ (10 mol%) in DMA (dimethylacetamide, 3 mL) was stirred at 85°C in air for 1 h, 22% yield of C2-trifluoromethylation product **3a** was obtained (Table 1, entry 1). Trace amount of product could be detected with other solvents, such as DCM (dichloromethane), toluene and THF (tetrahydrofuran) (entries 2-4). The yield could be increased to 46% when acetone was used, and it was elevated to 54% by using MeCN (acetonitrile) (entries 5-6). Subsequently, various metal catalysts were selected. To our delighted, the yield was increased to 65% by employing CuSO_4_•5H_2_O as catalyst (entry 7). Meanwhile, other catalysts such as FeCl_3_, FeCl_2_, Cu(OTf)_2_, and CuCl were screened. Unfortunately, no positive results were obtained (entries 8-11). In addition, the reaction was completely shut down in the absence of metal catalysts (entry 12). Finally, the desired product **3a** was obtained in 86% isolated yield when the solvent was reduced to 1 mL (entry 13). The reaction showed low reactivity at room temperature (entry 14). Afterward, the efficiency of different directing groups was investigated. And no desired product was achieved when Ac, Ts and 2-pym were tried (entries 15-17). In addition, the use of the methyl group resulted in a marked decreased in selectivity and yield (entry 18).

**Table 1:** Optimization of reaction conditions^a^.

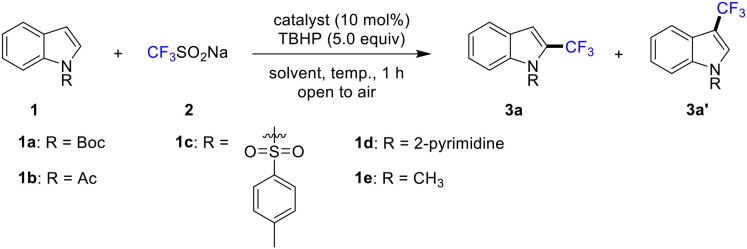					
**Entry**	**1**	**Cat**.	**Solvent**	**Yield^b^**	
				**3a**	**3a'**
1	**1a**	CuSO_4_	DMA	22	n.d.
2	**1a**	CuSO_4_	DCM	trace	n.d.
3	**1a**	CuSO_4_	toluene	trace	n.d.
4	**1a**	CuSO_4_	THF	trace	n.d.
5	**1a**	CuSO_4_	MeCN	54	6
6	**1a**	CuSO_4_	acetone	46	8
7	**1a**	CuSO_4_•5H_2_O	MeCN	65	<5
8	**1a**	FeCl_3_	MeCN	21	n.d.
9	**1a**	FeCl_2_	MeCN	23	n.d.
10	**1a**	Cu(OTf)_2_	MeCN	trace	n.d.
11	**1a**	CuCl	MeCN	12	n.d.
12	**1a**	-	MeCN	trace	n.d.
**13**	**1a**	**CuSO**_4_•**5H**_**2**_**O**	**MeCN**^c^	**89(86)**	** <5**
14	**1a**	CuSO_4_•5H_2_O	MeCN^c^^,^^d^	58	7
15	**1b**	CuSO_4_•5H_2_O	MeCN	n.d.	n.d.
16	**1c**	CuSO_4_•5H_2_O	MeCN	n.d.	n.d.
17	**1d**	CuSO_4_•5H_2_O	MeCN	n.d.	n.d.
18	**1e**	CuSO_4_•5H_2_O	MeCN	12	9

a *Conditions: **1a** (0.5 mmol), **2** (1.5 mmol), catalyst (10 mol %), solvent (3.0 mL), 85°C, 1 h, in air*.

b *Reported yields were based on **3a** and determined by ^1^H NMR using CH_2_Br_2_ as an internal standard*.

c *MeCN (1 ml)*.

d *room temperature, 12 h*.

**Scheme 3:**
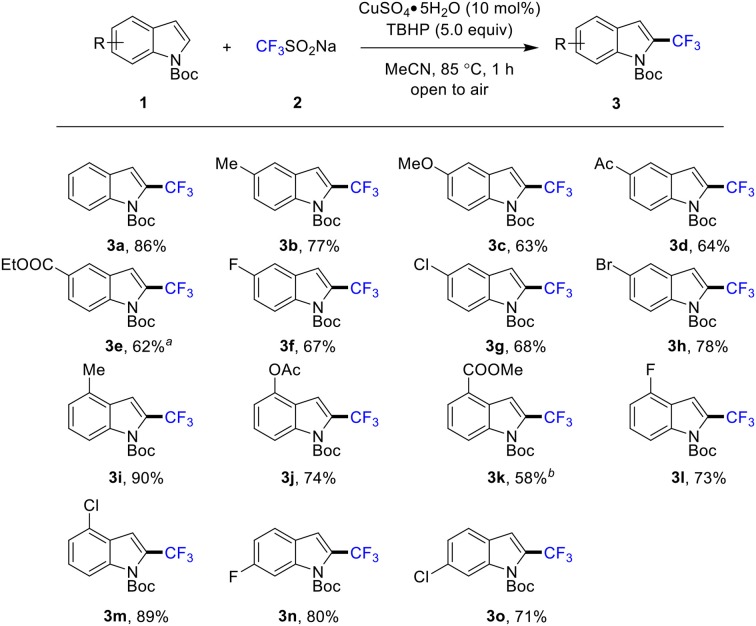
Scope of indoles. Conditions: **1** (0.5 mmol), **2** (1.5 mmol), CuSO_4_•5H_2_O (10 mol %), MeCN (1.0 mL), 85°C, 1 h, in air. Isolated yield. ^*a*^ 12 h. ^*b*^ 5 h.

With an optimized protocol in hand, the scope and limitation of the title reaction was explored (Scheme 3). With respect to the various indole derivatives, the reaction was found quite general and tolerated by various functional groups. A wide range of 2-trifluoromethylated products with substituent groups such as methyl (**3b**, **3i**), methoxy (**3c**), acetyl (**3d**), esters (**3e**, **3j**, **3k**), and halogen (**3f**-**3h**, **3l**-**3o**) at 4-, 5- and 6-position of indole were produced in moderate to good yields. In particular, halides, such as F, Cl, and Br, were well tolerated, affording the desired 2-trifluoromethylated products (**3f**-**3h** and **3l**-**3o**) in good yields of 67–89%. However, C7-substituted indoles are not reactive under the optimized reaction conditions, which is presumed due to the steric hindrance. In addition, owing to the strong electron-withdrawing property, the indoles containing cyanide and nitro are not reactive.

**Scheme 4:**
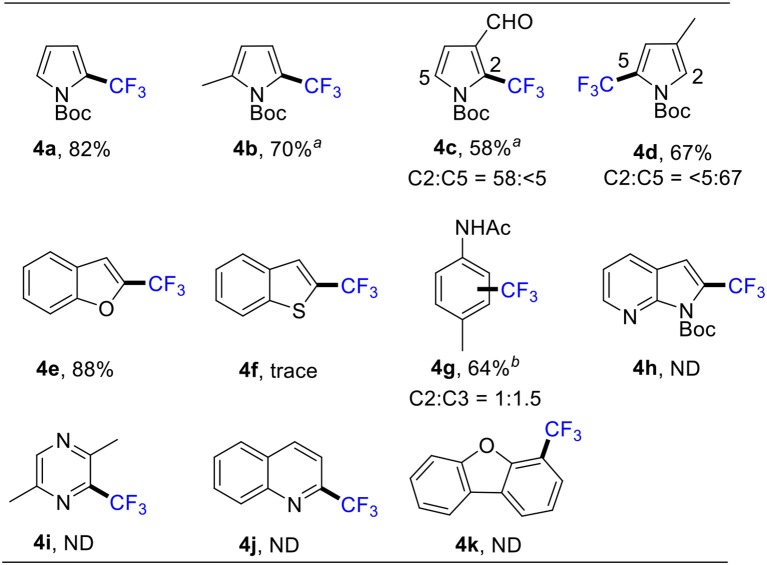
Scope of other heteroarenes. Conditions: **1** (0.5 mmol), **2** (1.5 mmol), CuSO_4_•5H_2_O (10 mol %), MeCN (1.0 mL), 85°C, 1 h, in air. Isolated yield. ^*a*^ NMR yield. ^*b*^ Using 6 equiv of **2** and 10 equiv of TBHP, 12h.

To extend the substrate scope of the above reaction, we proceeded to study the trifluoromethylation of other aromatics under the optimized reaction conditions. As shown in Scheme 4, pyrroles reacted smoothly to afford the corresponding 2-trifluoromethylated pyrroles (**4a**-**4d**) in good yields. Conventionally, direct deprotonation of benzofuran takes place at the most acidic C2 position (Larbi et al., 2017; Wang et al., 2018). Following C2 deprotonation, we obtained 2-trifluoromethylated benzofuran **4e** in 88% through a radical addition mechanism. Notably, benzothiophene was also examined, but only a trace amount of product **4f** was detected. Acetanilide, a drug to relieve pain or reduce fever, was also used for the synthesis of **4g**. Additionally, we tried other “indole-like” compounds, but the products (**4h**-**4k**) were not gained.

## Mechanism

**Scheme 5:**
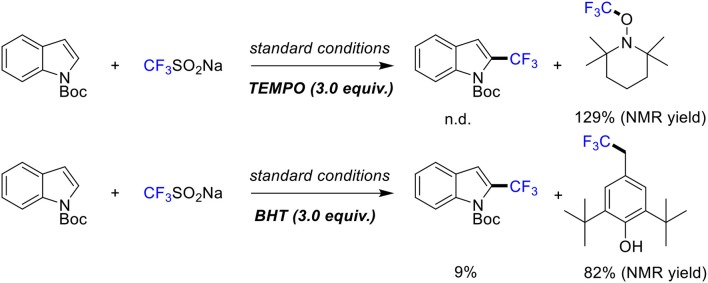
Mechanistic study.

The radical scavenger experiments were conducted to gain some insights into the mechanism of this reaction (Scheme 5). When radical inhibitors such as TEMPO (2,2,6,6-tetramethyl-1-piperidinyloxy) and BHT (butylated hydroxytoluene) were added, the reaction was suppressed to a great extent. Also, ^19^F NMR analysis showed that radical trapping product TEMPO/BHT-CF_3_ was formed dominantly. Therefore, we speculated that the high C2 selective is due to the formation of a five membered metallacycle at the C2 position through *N*-Boc-directed C-H activation (Sandtorv, 2015; Yang et al., 2016).

**Scheme 6:**
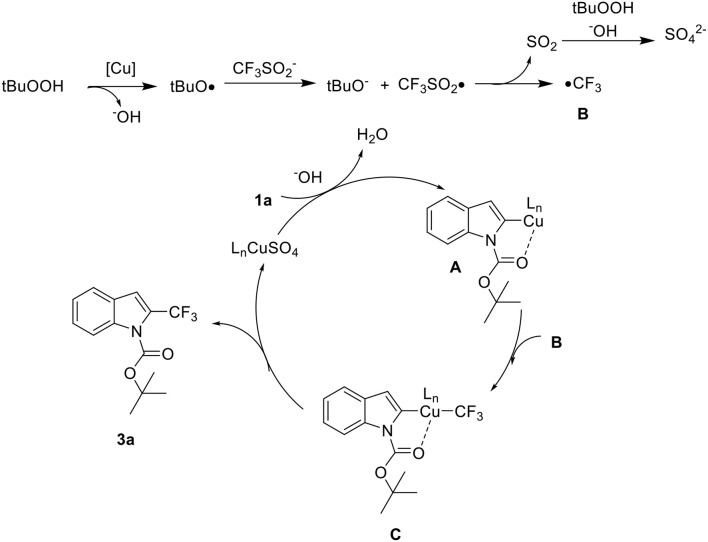
Proposed reaction mechanism. L = H_2_O, O_2_, or solvent.

Based on the analysis of the aforementioned results and previous reports, a plausible mechanism was proposed in Scheme 6 (Langlois et al., 1991; Ji et al., 2011; Zhang et al., 2018; Khan et al., 2019). Initially, the *t*-butoxy radical, generated from copper metal, reacts with CF_3_${\text{SO}}_{2}^{-}$ to provide •CF_3_SO_2_. This transient intermediate disproportionates, releasing SO_2_ and •CF_3_ **B**. Meanwhile, the copper catalyst was introduced to ensure the formation of a five membered metallacycle **A** at the C2 position. Subsequently, chelation-assisted C-H metalation of **1a A** reacts with **B** to form **C** as a key intermediate. After reductive elimination the product **3a** was formed and copper(II) catalyst regenerated.

## Conclusion

In conclusion, we have developed a direct C2-H trifluoromethylation of indoles with the assistance of a removable directing group under ambient conditions. This transformation exhibits high regioselectivity, functional group tolerance and provide a practical method to various trifluoromethylated heteroarenes including indoles, pyrroles, benzofuran, and acetanilide. What is more, control experiments testified that a radical mechanism may be involved in the reaction.

## Materials and Methods

### General

^1^H NMR spectra were recorded on Bruker 500 MHz spectrometer and the chemical shifts were reported in parts per million (δ) relative to internal standard TMS (0 ppm) for CDCl_3_. The peak patterns are indicated as follows: s, singlet; d, doublet; dd, doublet of doublet; t, triplet; m, multiplet; q, quartet. The coupling constants, *J*, are reported in Hertz (Hz). ^13^C NMR spectra were obtained at Bruker 126 MHz and referenced to the internal solvent signals (central peak is 77.0 ppm in CDCl_3_). The NMR yield was determined by ^1^H NMR using CH_2_Br_2_ as an internal standard. APEX II (Bruker Inc.) was used for ESI-HRMS. Flash column chromatography was performed over silica gel 200–300. All reagents were weighed and handled in air at room temperature. All chemical reagents were purchased from Alfa, Acros, Aldrich, and TCI, J&K and used without further purification.

### General Procedure and Characterization Data for Product 3, 4

To a mixture of *N*-Boc indole **1** (0.5 mmol), CF_3_SO_2_Na **2** (1.5 mmol) and CuSO_4_•5H_2_O (10 mol%), MeCN (1.0 mL) was added in air at room temperature. *tert*-Butyl hydroperoxide (TBHP, 70% solution in H_2_O, 2.5 mmol) was dropped into the mixture in air at room temperature. The resulting mixture was stirred at 85°C in air for 1 h. Once the mixture was cooled to room temperature, the solvent was removed under reduced pressure. The crude product was purified by flash column chromatography on silica gel (petroleum ether/CH_2_Cl_2_) to give product **3** or **4** as colorless oil. The NMR spectra of synthesized compounds are depicted in Supplementary Material.

#### Tert-Butyl 2-(Trifluoromethyl)-1H-Indole-1-Carboxylate (3a) (Xu et al., 2011; Arimori and Shibata, 2015) (123 mg, 86%)

Isolated by flash column chromatography (petroleum ether/CH_2_Cl_2_ = 50:1, R_f_ = 0.3). ^1^H NMR (500 MHz, CDCl_3_) δ 8.28 (d, *J* = 8.5 Hz, 1H), 7.62 (d, *J* = 7.9 Hz, 1H), 7.45 (t, *J* = 7.9 Hz, 1H), 7.30 (t, *J* = 7.5 Hz, 1H), 7.14 (s, 1H), 1.68 (s, 9H). ^13^C NMR (126 MHz, CDCl_3_) δ 148.57, 137.70, 126.98, 126.93 (q, *J* = 38.9 Hz), 126.46, 123.51, 122.77 (q, *J* = 266.6 Hz), 121.99, 116.04, 113.43 (q, *J* = 5.0 Hz), 85.43, 27.86. ^19^F NMR (470 MHz, CDCl_3_) δ−58.15. HRMS (ESI) caculated for C_9_H_5_NF_3_ [M-Boc]^−^, 184.0374; found: 184.0380.

#### Tert-Butyl 5-Methyl-2-(Trifluoromethyl)-1H-Indole-1-Carboxylate (3b) (115 mg, 77%)

Isolated by flash column chromatography (petroleum ether/CH_2_Cl_2_ = 50:1, R_f_ = 0.3). ^1^H NMR (500 MHz, CDCl_3_) δ 8.14 (d, *J* = 8.7 Hz, 1H), 7.39 (s, 1H), 7.27 (d, *J* = 7.5 Hz, 1H), 7.06 (s, 1H), 2.45 (s, 3H), 1.67 (s, 9H). ^13^C NMR (126 MHz, CDCl_3_) δ 148.61, 135.95, 133.07, 128.52, 126.83 (q, *J* = 40.4 Hz), 126.65, 121.63, 120.78 (q, *J* = 266.1 Hz), 115.67, 113.19 (q, *J* = 5.1 Hz), 85.20, 27.86, 21.17. ^19^F NMR (470 MHz, CDCl_3_) δ−58.15. HRMS (ESI) caculated for C_10_H_7_NF_3_ [M-Boc]^−^, 198.0531; found: 198.0536.

#### Tert-Butyl 5-Methoxy-2-(Trifluoromethyl)-1H-Indole-1-Carboxylate (3c) (99 mg, 63%)

Isolated by flash column chromatography (petroleum ether/CH_2_Cl_2_ = 50:1, R_f_ = 0.3). ^1^H NMR (500 MHz, CDCl_3_) δ 8.17 (d, *J* = 9.2 Hz, 1H), 7.08 – 7.05 (m, 2H), 7.03 (d, *J* = 2.4 Hz, 1H), 3.86 (s, 3H), 1.66 (s, 9H). ^13^C NMR (126 MHz, CDCl_3_) δ 156.25, 148.52, 132.44, 127.24 (q, *J* = 38.6 Hz), 127.17, 120.68 (q, *J* = 266.4 Hz), 116.98, 116.53, 113.14 (q, *J* = 5.1 Hz), 103.45, 85.26, 55.63, 27.86. ^19^F NMR (470 MHz, CDCl_3_) δ−58.24. HRMS (ESI) caculated for C_10_H_7_ONF_3_ [M-Boc]^−^, 214.0480; found: 214.0485.

#### Tert-Butyl 5-Acetyl-2-(Trifluoromethyl)-1H-Indole-1-Carboxylate (3d) (105 mg, 64%)

Isolated by flash column chromatography (petroleum ether/ ethyl acetate = 50:1, R_f_ = 0.3). ^1^H NMR (500 MHz, CDCl_3_) δ 8.34 (d, *J* = 8.9 Hz, 1H), 8.25 (d, *J* = 1.2 Hz, 1H), 8.06 (dd, *J* = 9.0, 1.7 Hz, 1H), 7.22 (s, 1H), 2.67 (s, 3H), 1.68 (s, 9H). ^13^C NMR (126 MHz, CDCl_3_) δ 197.39, 148.14, 140.21, 132.93, 128.42 (q, *J* = 38.9 Hz), 126.85, 126.23, 123.23, 122.52 (q, *J* = 266.9 Hz), 116.06, 113.85 (q, *J* = 5.0 Hz), 86.31, 27.78, 26.68. ^19^F NMR (470 MHz, CDCl_3_) δ−58.38. HRMS (ESI) caculated for C_11_H_7_ONF_3_ [M-Boc]^−^, 226.0480; found: 226.0485.

#### 1-(Tert-Butyl) 5-Ethyl 2-(Trifluoromethyl)-1H-Indole-1,5-Dicarboxylate (3e) (111 mg, 62%)

Isolated by flash column chromatography (petroleum ether/ ethyl acetate = 50:1, R_f_ = 0.3). ^1^H NMR (500 MHz, CDCl_3_) δ 8.36 (d, J = 0.9 Hz, 1H), 8.32 (d, J = 8.9 Hz, 1H), 8.13 (dd, J = 8.9, 1.5 Hz, 1H), 7.20 (s, 1H), 4.41 (q, J = 7.1 Hz, 2H), 1.68 (s, 9H), 1.43 (t, J = 7.1 Hz, 3H). ^13^C NMR (126 MHz, CDCl_3_) δ 166.43, 148.21, 140.16, 128.23 (q, *J* = 39.3 Hz), 127.97, 126.15, 125.99, 124.32, 120.45 (q, *J* = 266.5 Hz), 115.83, 113.70 (q, *J* = 5.0 Hz), 86.18, 61.08, 27.79, 14.36. ^19^F NMR (470 MHz, CDCl_3_) δ−58.35. HRMS (ESI) caculated for C_12_H_9_F_3_NO_2_ [M-Boc]^−^, 256.0585; found: 256.0591.

#### Tert-Butyl 5-Fluoro-2-(Trifluoromethyl)-1H-Indole-1-Carboxylate (3f) (102 mg, 67%)

Isolated by flash column chromatography (petroleum ether/CH_2_Cl_2_ = 50:1, R_f_ = 0.3). ^1^H NMR (500 MHz, CDCl_3_) δ 8.26 (dd, *J* = 9.2, 4.5 Hz, 1H), 7.28 – 7.25 (m, 1H), 7.18 (td, *J* = 9.2, 2.5 Hz, 1H), 7.09 (s, 1H), 1.67 (s, 9H). ^13^C NMR (126 MHz, CDCl_3_) δ 160.30, 158.38, 148.34, 134.09, 128.30 (q, *J* = 39.1 Hz), 120.47 (q, *J* = 266.5 Hz), 117.41 (d, *J* = 8.9 Hz), 115.22 (d, *J* = 25.0 Hz), 112.84 (q, *J* = 4.9 Hz), 107.07 (d, *J* = 23.8 Hz), 85.80, 27.81. ^19^F NMR (470 MHz, CDCl_3_) δ−58.42,−119.41 (td, *J* = 8.5, 4.7 Hz). HRMS (ESI) caculated for C_9_H_4_NF_4_ [M-Boc]^−^, 202.0280; found: 202.0285.

#### Tert-Butyl 5-Chloro-2-(Trifluoromethyl)-1H-Indole-1-Carboxylate (3g) (109 mg, 68%)

Isolated by flash column chromatography (petroleum ether/CH_2_Cl_2_ = 50:1, R_f_ = 0.3). ^1^H NMR (500 MHz, CDCl_3_) δ 8.22 (d, *J* = 9.0 Hz, 1H), 7.58 (d, *J* = 2.0 Hz, 1H), 7.39 (dd, *J* = 9.0, 2.0 Hz, 1H), 7.06 (s, 1H), 1.67 (s, 9H). ^13^C NMR (126 MHz, CDCl_3_) δ 148.22, 136.05, 129.18, 128.10 (q, *J* = 39.3 Hz), 127.49, 127.28, 121.36, 120.43 (q, *J* = 266.5 Hz), 117.28, 112.45 (q, *J* = 5.1 Hz), 85.98, 27.80. ^19^F NMR (470 MHz, CDCl_3_) δ−58.39. HRMS (ESI) caculated for C_9_H_4_NClF_3_ [M-Boc]^−^, 217.9984; found: 217.9990.

#### Tert-Butyl 5-Bromo-2-(Trifluoromethyl)-1H-Indole-1-Carboxylate (3h) (142 mg, 78%)

Isolated by flash column chromatography (petroleum ether/CH_2_Cl_2_ = 50:1, R_f_ = 0.3). ^1^H NMR (500 MHz, CDCl_3_) δ 8.17 (d, *J* = 9.0 Hz, 1H), 7.75 (d, *J* = 1.7 Hz, 1H), 7.53 (dd, *J* = 9.0, 1.9 Hz, 1H), 7.06 (s, 1H), 1.67 (s, 9H). ^13^C NMR (126 MHz, CDCl_3_) δ 148.20, 136.41, 129.92, 128.03, 127.95 (q, *J* = 39.2 Hz), 124.48, 120.39 (q, *J* = 266.5 Hz), 117.62, 116.75, 112.32 (q, *J* = 5.0 Hz), 86.01, 27.80. ^19^F NMR (470 MHz, CDCl_3_) δ−58.37. HRMS (ESI) caculated for C_9_H_4_NBrF_3_ [M-Boc]^−^, 261.9479; found: 261.9485.

#### Tert-Butyl 4-Methyl-2-(Trifluoromethyl)-1H-Indole-1-Carboxylate (3i) (135 mg, 90%)

Isolated by flash column chromatography (petroleum ether/CH_2_Cl_2_ = 50:1, *R*_f_ = 0.3). ^1^H NMR (500 MHz, CDCl_3_) δ 8.10 (d, *J* = 8.5 Hz, 1H), 7.34 (t, *J* = 7.9 Hz, 1H), 7.18 (s, 1H), 7.09 (d, *J* = 7.2 Hz, 1H), 2.54 (s, 3H), 1.67 (s, 9H). ^13^C NMR (126 MHz, CDCl_3_) δ 148.63, 137.60, 131.57, 127.10, 126.31 (q, *J* = 38.9 Hz), 126.22, 123.81, 120.85 (q, *J* = 266.3 Hz), 113.51, 111.86 (q, *J* = 5.1 Hz), 85.33, 27.85, 18.23. ^19^F NMR (470 MHz, CDCl_3_) δ−57.97. HRMS (ESI) caculated for C_10_H_7_NF_3_ [M-Boc]^−^, 198.0531; found: 198.0536.

#### Tert-Butyl 4-Acetoxy-2-(Trifluoromethyl)-1H-Indole-1-Carboxylate (3j) (127 mg, 74%)

Isolated by flash column chromatography (petroleum ether/ ethyl acetate = 100:1, *R*_f_ = 0.3). ^1^H NMR (500 MHz, CDCl_3_) δ 8.16 (d, *J* = 8.6 Hz, 1H), 7.43 (t, *J* = 8.2 Hz, 1H), 7.07 (dd, *J* = 7.9, 0.6 Hz, 1H), 7.05 (s, 1H), 2.40 (s, 3H), 1.67 (s, 9H). ^13^C NMR (126 MHz, CDCl_3_) δ 168.91, 148.30, 144.04, 138.98, 127.46, 127.17 (q, *J* = 39.0 Hz), 120.46 (q, *J* = 266.4 Hz), 120.07, 115.80, 113.96, 109.74 (q, *J* = 5.3 Hz), 85.88, 27.80, 20.96. ^19^F NMR (470 MHz, CDCl_3_) δ−58.27. HRMS (ESI) caculated for C_11_H_7_O_2_NF_3_ [M-Boc]^−^, 242.0429; found: 242.0434.

#### 1-(Tert-Butyl) 4-Methyl 2-(Trifluoromethyl)-1H-Indole-1,4-Dicarboxylate (3k) (100 mg, 58%)

Isolated by flash column chromatography (petroleum ether/ ethyl acetate = 50:1, *R*_f_ = 0.3). ^1^H NMR (500 MHz, CDCl_3_) δ 8.54 (d, *J* = 8.5 Hz, 1H), 8.04 (dd, *J* = 7.6, 0.8 Hz, 1H), 7.85 (s, 1H), 7.50 (t, *J* = 8.2 Hz, 1H), 4.00 (s, 3H), 1.67 (s, 9H). ^13^C NMR (126 MHz, CDCl_3_) δ 166.68, 148.35, 138.28, 128.38 (q, *J* = 38.9 Hz), 126.41, 126.34, 126.14, 123.10, 120.72, 120.59 (q, *J* = 266.8 Hz), 113.77 (q, *J* = 5.4 Hz), 86.04, 52.13, 27.79. ^19^F NMR (470 MHz, CDCl_3_) δ−58.20. HRMS (ESI) caculated for C_11_H_7_O_2_NF_3_ [M-Boc]^−^, 242.0429; found: 242.0434.

#### Tert-Butyl 4-Fluoro-2-(Trifluoromethyl)-1H-Indole-1-Carboxylate (3l) (111 mg, 73%)

Isolated by flash column chromatography (petroleum ether/CH_2_Cl_2_ = 50:1, R_f_ = 0.3). ^1^H NMR (500 MHz, CDCl_3_) δ 8.06 (d, *J* = 8.6 Hz, 1H), 7.38 (td, *J* = 8.3, 5.6 Hz, 1H), 7.24 (s, 1H), 6.97 (t, *J* = 9.0, 1H), 1.67 (s, 9H). ^13^C NMR (126 MHz, CDCl_3_) δ 157.23, 155.23, 148.34, 139.54, 127.84 (d, *J* = 7.5 Hz), 127.01 (q, *J* = 39.1 Hz), 120.42 (q, *J* = 266.5 Hz), 112.11 (d, *J* = 4.0 Hz), 108.91 (q, *J* = 5.4 Hz), 108.51 (d, *J* = 17.9 Hz), 86.02, 27.80. ^19^F NMR (470 MHz, CDCl_3_) δ−58.31,−120.93 (q, *J* = 9.4 Hz). HRMS (ESI) caculated for C_9_H_4_NF_4_ [M-Boc]^−^, 202.0280; found: 202.0285.

#### Tert-Butyl 4-Chloro-2-(Trifluoromethyl)-1H-Indole-1-Carboxylate (3m) (142 mg, 89%)

Isolated by flash column chromatography (petroleum ether/CH_2_Cl_2_ = 50:1, R_f_ = 0.3). ^1^H NMR (500 MHz, CDCl_3_) δ 8.19 (d, *J* = 8.5 Hz, 1H), 7.36 (t, *J* = 8.1 Hz, 1H), 7.29 (dd, *J* = 7.8, 0.6 Hz, 1H), 7.27 (s, 1H), 1.67 (s, 9H). ^13^C NMR (126 MHz, CDCl_3_) δ 148.23, 138.29, 127.62, 127.46 (q, *J* = 35.8 Hz), 127.28, 125.45, 123.27, 120.46 (q, *J* = 266.3 Hz), 114.66, 111.37 (q, *J* = 5.3 Hz), 86.10, 27.80. ^19^F NMR (470 MHz, CDCl_3_) δ−58.30. HRMS (ESI) caculated for C_9_H_4_NClF_3_ [M-Boc]^−^, 217.9984; found: 217.9990.

#### Tert-Butyl 6-Fluoro-2-(Trifluoromethyl)-1H-Indole-1-Carboxylate (3n) (121 mg, 80%)

Isolated by flash column chromatography (petroleum ether/CH_2_Cl_2_ = 50:1, R_f_ = 0.3). ^1^H NMR (500 MHz, CDCl_3_) δ 8.02 (dd, *J* = 10.6, 1.8 Hz, 1H), 7.55 (dd, *J* = 8.5, 5.5 Hz, 1H), 7.10 (s, 1H), 7.06 (td, *J* = 8.7, 1.7 Hz, 1H), 1.67 (s, 9H). ^13^C NMR (126 MHz, CDCl_3_) δ 163.29, 161.36, 148.33, 122.93 (d, *J* = 10.1 Hz), 122.74, 120.51 (q, *J* = 266.1 Hz), 113.15 (q, *J* = 4.9 Hz), 112.38 (d, *J* = 24.6 Hz), 103.36 (d, *J* = 29.1 Hz), 102.90 (q, *J* = 28.5 Hz), 85.92, 27.81. ^19^F NMR (470 MHz, CDCl_3_) δ−58.26,−112.94 (td, *J* = 9.6, 5.6 Hz). HRMS (ESI) caculated for C_9_H_4_NF_4_ [M-Boc]^−^, 202.0280; found: 202.0285.

### Tert-Butyl 6-Chloro-2-(Trifluoromethyl)-1H-Indole-1-Carboxylate (3o) (113 mg, 71%)

Isolated by flash column chromatography (petroleum ether/CH_2_Cl_2_ = 50:1, R_f_ = 0.3). ^1^H NMR (500 MHz, CDCl_3_) δ 8.35 (s, 1H), 7.53 (d, *J* = 8.4 Hz, 1H), 7.30 – 7.26 (m, 1H), 7.10 (s, 1H), 1.67 (s, 9H). ^13^C NMR (126 MHz, CDCl_3_) δ 148.22, 138.03, 133.12, 127.48 (q, *J* = 39.1 Hz), 124.87, 124.37, 122.72, 120.48 (q, *J* = 266.5 Hz), 116.37, 113.03 (q, *J* = 5.0 Hz), 86.07, 27.80. ^19^F NMR (470 MHz, CDCl_3_) δ−58.30. HRMS (ESI) caculated for C_9_H_4_NClF_3_ [M-Boc]^−^, 217.9984; found: 217.9990.

#### Tert-Butyl 2-(Trifluoromethyl)-1H-Pyrrole-1-Carboxylate (4a) (Nagib and MacMillan, 2011; Du et al., 2017) (96 mg, 82%)

Isolated by flash column chromatography (petroleum ether/CH_2_Cl_2_ = 50:1, R_f_ = 0.3). ^1^H NMR (500 MHz, CDCl_3_) δ 7.44 (d, *J* = 1.9 Hz, 1H), 6.73 (s, 1H), 6.19 (t, *J* = 3.2 Hz, 1H), 1.61 (s, 9H). ^13^C NMR (126 MHz, CDCl_3_) δ 125.78, 120.53 (q, *J* = 264.9 Hz), 117.76 (q, *J* = 4.6 Hz), 109.59, 85.62, 27.71. ^19^F NMR (470 MHz, CDCl_3_) δ−58.33. HRMS (ESI) caculated for C_5_H_3_NF_3_ [M-Boc]^−^, 134.0218; found: 134.0223.

#### Tert-Butyl 2-Methyl-5-(Trifluoromethyl)-1H-Pyrrole-1-Carboxylate (4b)

Isolated by flash column chromatography (petroleum ether/CH_2_Cl_2_ = 50:1, R_f_ = 0.3). ^1^H NMR (500 MHz, CDCl_3_) δ 6.59 (d, *J* = 3.5 Hz, 1H), 5.92 (d, *J* = 3.2 Hz, 1H), 2.44 (s, 3H), 1.60 (s, 9H). ^13^C NMR (126 MHz, CDCl_3_) δ 148.43, 137.08, 121.58 (q, *J* = 39.3 Hz), 120.77 (q, *J* = 264.5 Hz), 116.00 (q, *J* = 4.8 Hz), 109.96, 85.39, 31.60, 27.63. ^19^F NMR (470 MHz, CDCl_3_) δ−57.19. HRMS (ESI) caculated for C_6_H_5_NF_3_ [M-Boc]^−^, 148.0374; found: 148.0380.

#### Tert-Butyl 3-Formyl-2-(Trifluoromethyl)-1H-Pyrrole-1-Carboxylate (4c)

Isolated by flash column chromatography (petroleum ether/ ethyl acetate = 50:1, R_f_ = 0.3). ^1^H NMR (500 MHz, cdcl_3_) δ 10.18 (s, 1H), 7.43 (d, *J* = 3.3 Hz, 1H), 6.72 (d, *J* = 3.4 Hz, 1H), 1.63 (s, 9H). ^13^C NMR (126 MHz, CDCl_3_) δ 185.88 (q, *J* = 5.6 Hz), 171.10, 146.75, 125.86, 123.69 (q, *J* = 41.4 Hz), 120.46 (q, *J* = 267.8 Hz), 109.08, 87.39, 27.57. ^19^F NMR (470 MHz, CDCl_3_) δ−54.31. HRMS (ESI) caculated for C_6_H_3_ONF_3_ [M-Boc]^−^, 162.0167; found: 162.0172.

#### Tert-Butyl 4-Methyl-2-(Trifluoromethyl)-1H-Pyrrole-1-Carboxylate (4d) (83 mg, 67%)

Isolated by flash column chromatography (petroleum ether/CH_2_Cl_2_ = 50:1, R_f_ = 0.3). ^1^H NMR (500 MHz, CDCl_3_) δ 7.32 (s, 1H), 6.01 (s, 1H), 2.21 (s, 3H), 1.59 (s, 9H). ^13^C NMR (126 MHz, CDCl_3_) δ 147.67, 128.96 (q, *J* = 2.6 Hz), 124.61, 121.68 (q, *J* = 266.0 Hz), 119.54 (q, *J* = 4.4 Hz), 117.06 (q, *J* = 38.3 Hz), 113.52, 85.09, 27.69. ^19^F NMR (376 MHz, CDCl_3_) δ−54.63. HRMS (ESI) caculated for C_6_H_5_NF_3_ [M-Boc]^−^, 148.0374; found: 148.0380.

#### 2-(Trifluoromethyl)Benzofuran (4e) (Liu and Shen, 2011) (82 mg, 88%)

Isolated by flash column chromatography (petroleum ether, R_f_ = 0.3). ^1^H NMR (500 MHz, CDCl_3_) δ 7.67 (d, *J* = 7.8 Hz, 1H), 7.58 (d, *J* = 8.0 Hz, 1H), 7.45 (t, *J* = 7.4 Hz, 1H), 7.34 (t, *J* = 7.2 Hz, 1H), 7.18 (s, 1H). ^13^C NMR (126 MHz, CDCl_3_) δ 155.13, 143.48 (q, *J* = 41.9 Hz), 126.90, 125.99, 123.95, 122.46, 119.31 (q, *J* = 266.5 Hz), 112.09, 108.09 (q, *J* = 3.1 Hz). ^19^F NMR (470 MHz, CDCl_3_) δ -64.87.

#### N-(4-Methyl-2-(Trifluoromethyl)Phenyl)Acetamide (4g) (Zou et al., 2019) (28 mg, 26%)

Isolated by flash column chromatography (petroleum ether/ ethyl acetate = 5:1, R_f_ = 0.3). ^1^H NMR (500 MHz, CDCl_3_) δ 7.95 (d, *J* = 8.1 Hz, 1H), 7.40 (s, 1H), 7.34 (d, *J* = 7.8 Hz, 2H), 2.36 (s, 3H), 2.19 (s, 3H). ^13^C NMR (126 MHz, CDCl_3_) δ 168.48, 134.75, 133.28, 132.45, 126.33 (q, *J* = 4.9 Hz), 125.20, 124.00 (q, *J* = 271.5 Hz), 120.65 (q, *J* = 29.4 Hz), 24.43, 20.79. ^19^F NMR (470 MHz, CDCl_3_) δ−60.67. HRMS (ESI) caculated for C_10_H_11_ONF_3_ [M+H]^+^, 218.0793; found: 218.0787.

#### N-(4-Methyl-3-(Trifluoromethyl)Phenyl)Acetamide (4g) (41 mg, 38%)

Isolated by flash column chromatography (petroleum ether/ ethyl acetate = 5:1, R_f_ = 0.3). ^1^H NMR (500 MHz, CDCl_3_) δ 7.64 (d, *J* = 10.7 Hz, 2H), 7.57 (s, 1H), 7.21 (d, *J* = 8.1 Hz, 1H), 2.42 (s, 3H), 2.17 (s, 3H). ^13^C NMR (126 MHz, CDCl_3_) δ 168.60, 135.70, 132.48, 132.26, 129.18 (q, *J* = 29.9 Hz), 124.13 (q, *J* = 272.4 Hz), 123.00, 117.40 (q, *J* = 5.9 Hz), 24.41, 18.70. ^19^F NMR (470 MHz, CDCl_3_) δ−61.97. HRMS (ESI) caculated for C_10_H_11_ONF_3_ [M+H]^+^, 218.0793; found: 218.0787.

## Data Availability

All datasets generated for this study are included in the manuscript/Supplementary Files.

## Author Contributions

XS, XianL, and DS constructed the workflow. XS synthesized and purified the compounds. XS and XiaoL performed the mass spectrometric analysis. XS completed the paper.

### Conflict of Interest Statement

The authors declare that the research was conducted in the absence of any commercial or financial relationships that could be construed as a potential conflict of interest.
