# Supplementary material for: Ligand- and Additive-Free 2-Position-Selective Trifluoromethylation of Heteroarenes Under Ambient Conditions
Source: Front Chem. 2019 Sep 6;7:613. doi: 10.3389/fchem.2019.00613 (PMC6743045; doi:10.3389/fchem.2019.00613)

Supplementary Material

**Ligand- and Additive-Free 2-Position-Selective Trifluoromethylation of Heteroarenes Under Ambient Conditions**

**Xiaolin Shi 1,2,3, Xiaowei Li 1,2,3, Xiangqian Li 4* and Dayong Shi 4***

^1 Key Laboratory of Experimental Marine Biology, Institute of Oceanology, Chinese Academy of Sciences, Qingdao, China^

^2 Laboratory for Marine Drugs and Bioproducts of Qingdao National Laboratory for Marine Science and Technology, Qingdao, China^

^3 University of Chinese Academy of Sciences, Beijing, China^

^4 State Key Laboratory of Microbial Technology, Shandong University, Qingdao, China^

[shidayong@sdu.edu.cn](mailto:shidayong@sdu.edu.cn)

**Table of Contents**

Copies of ^1^H, ^13^C, ^19^F NMR spectra for new compounds S2-S33


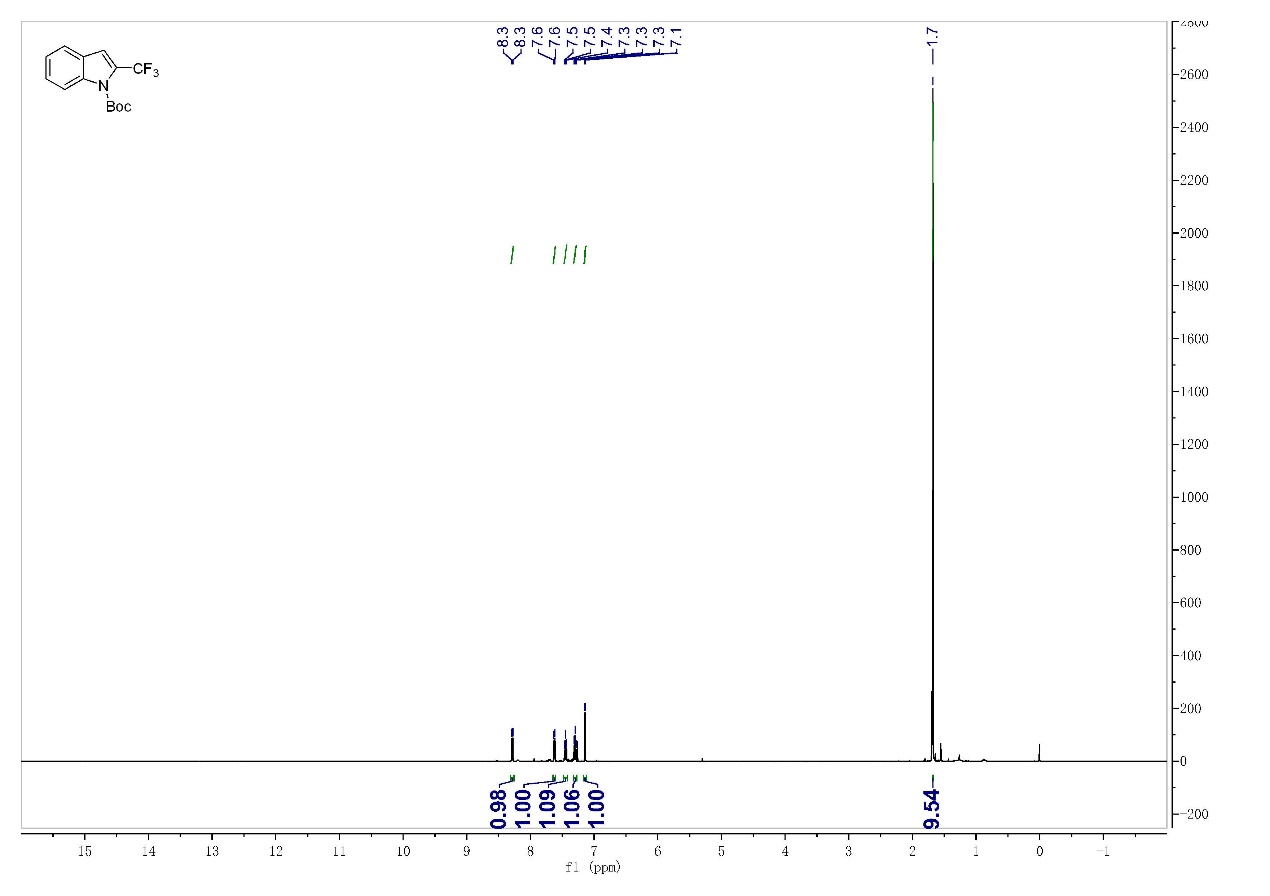


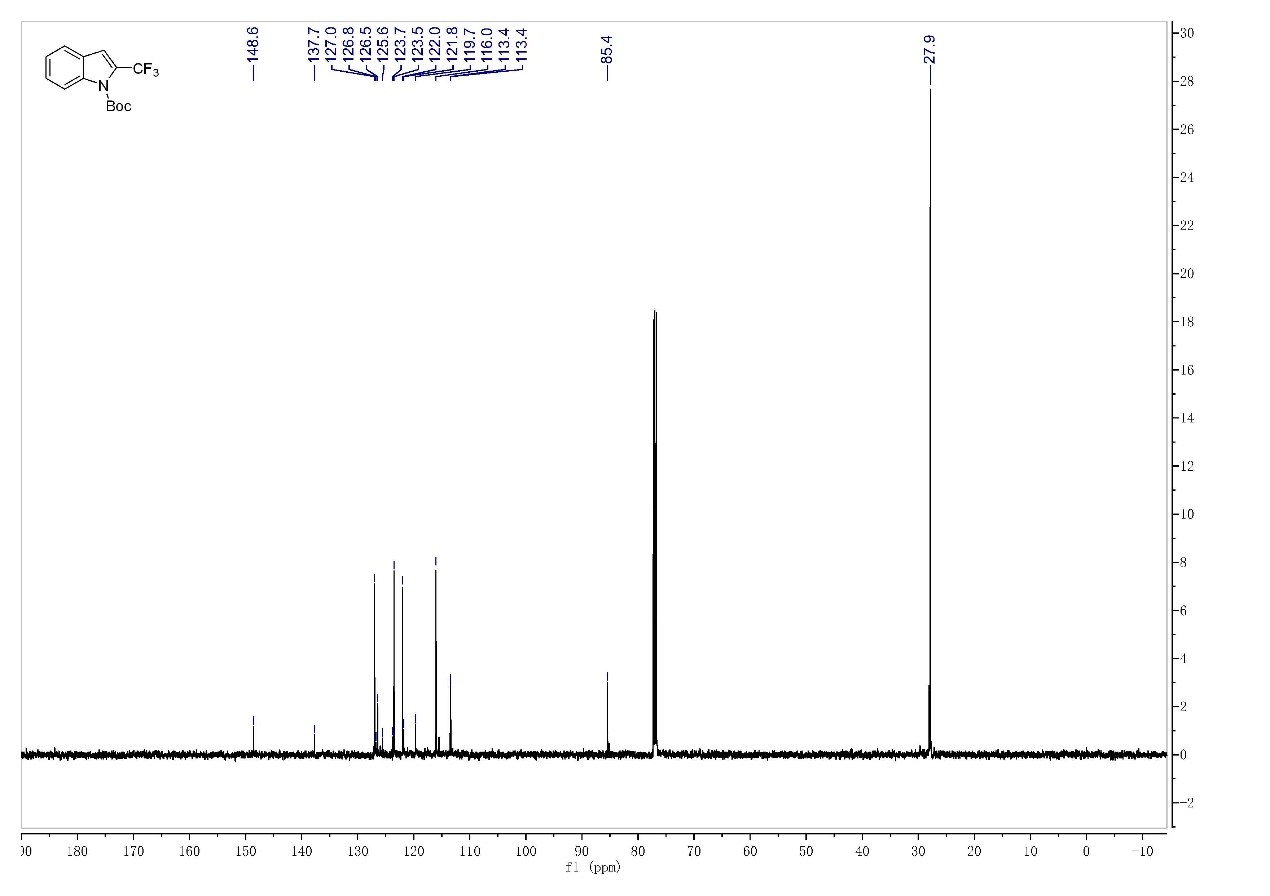

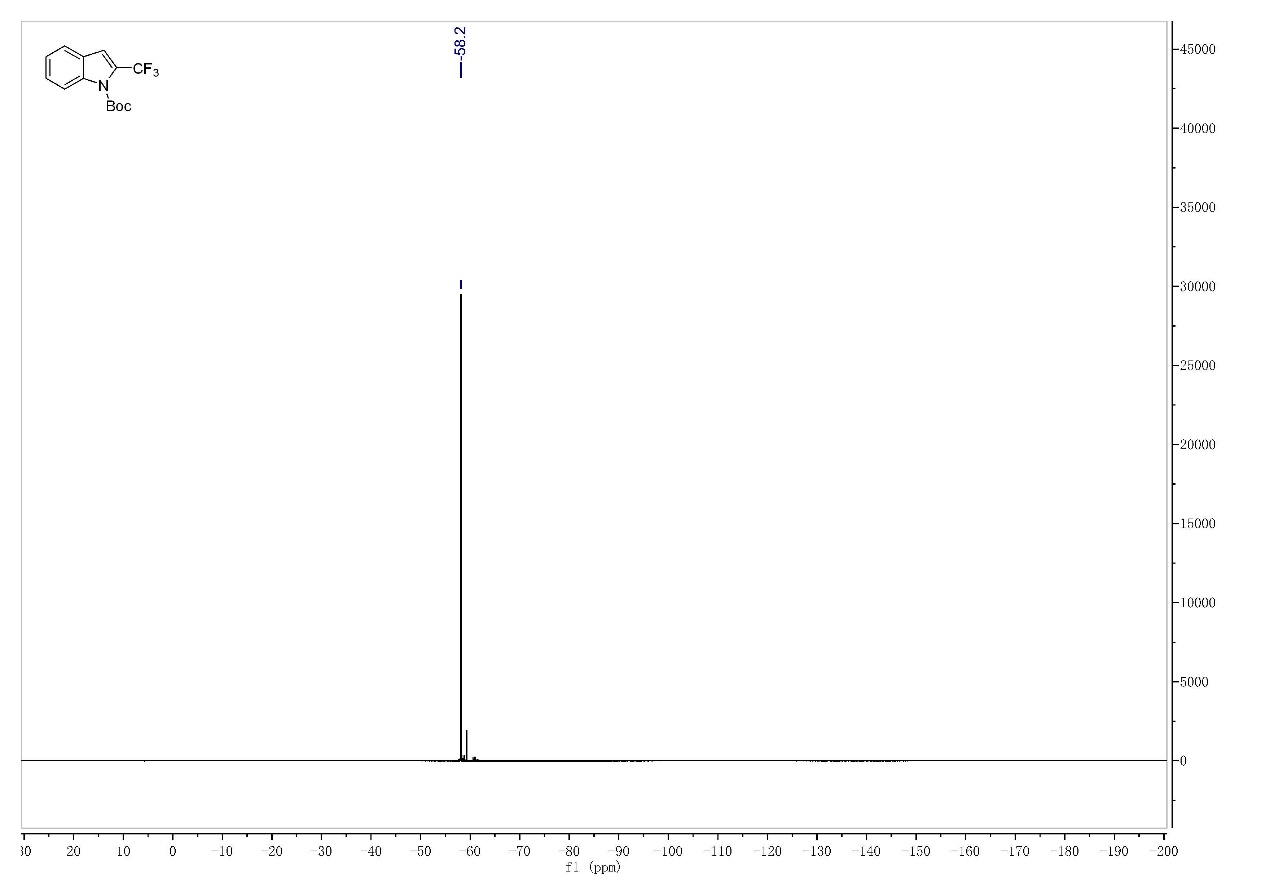

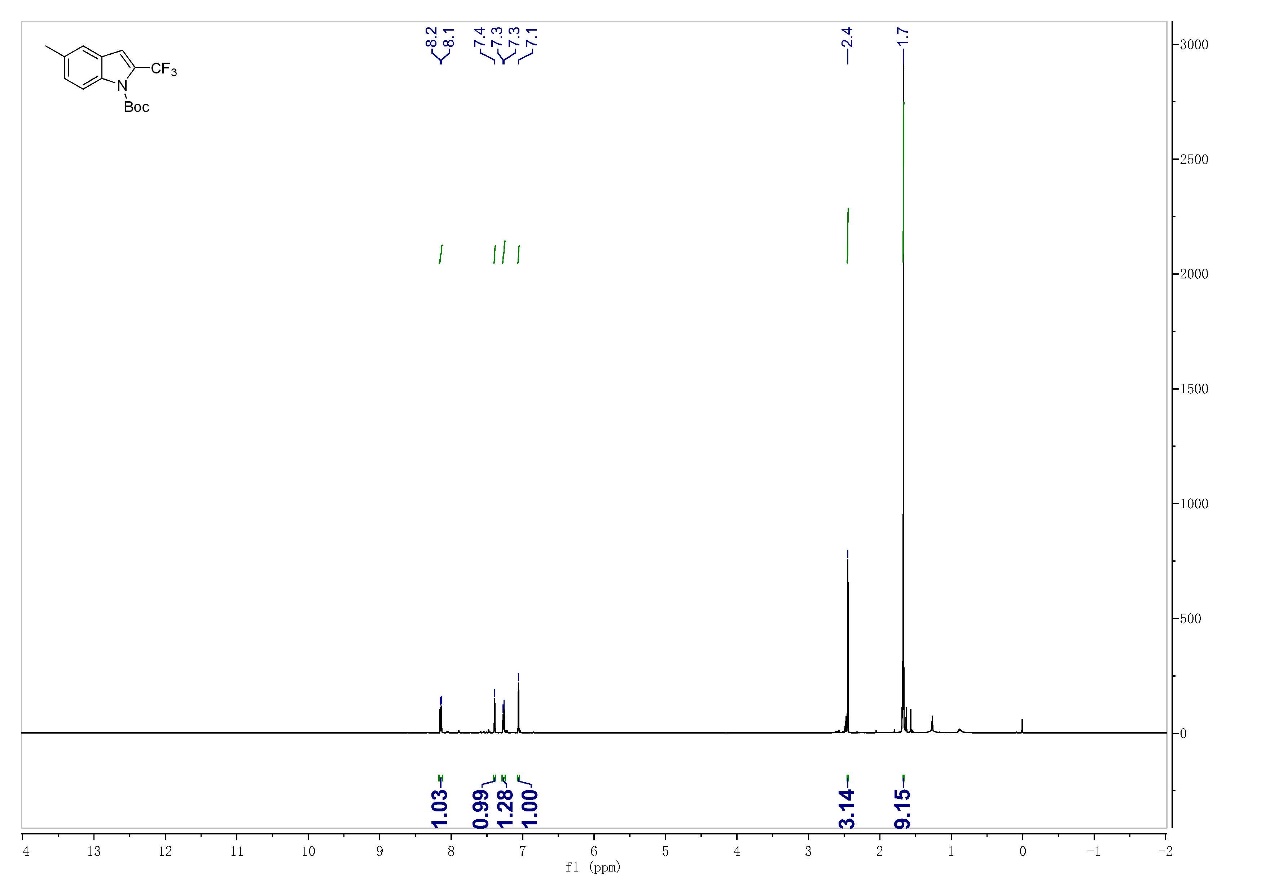

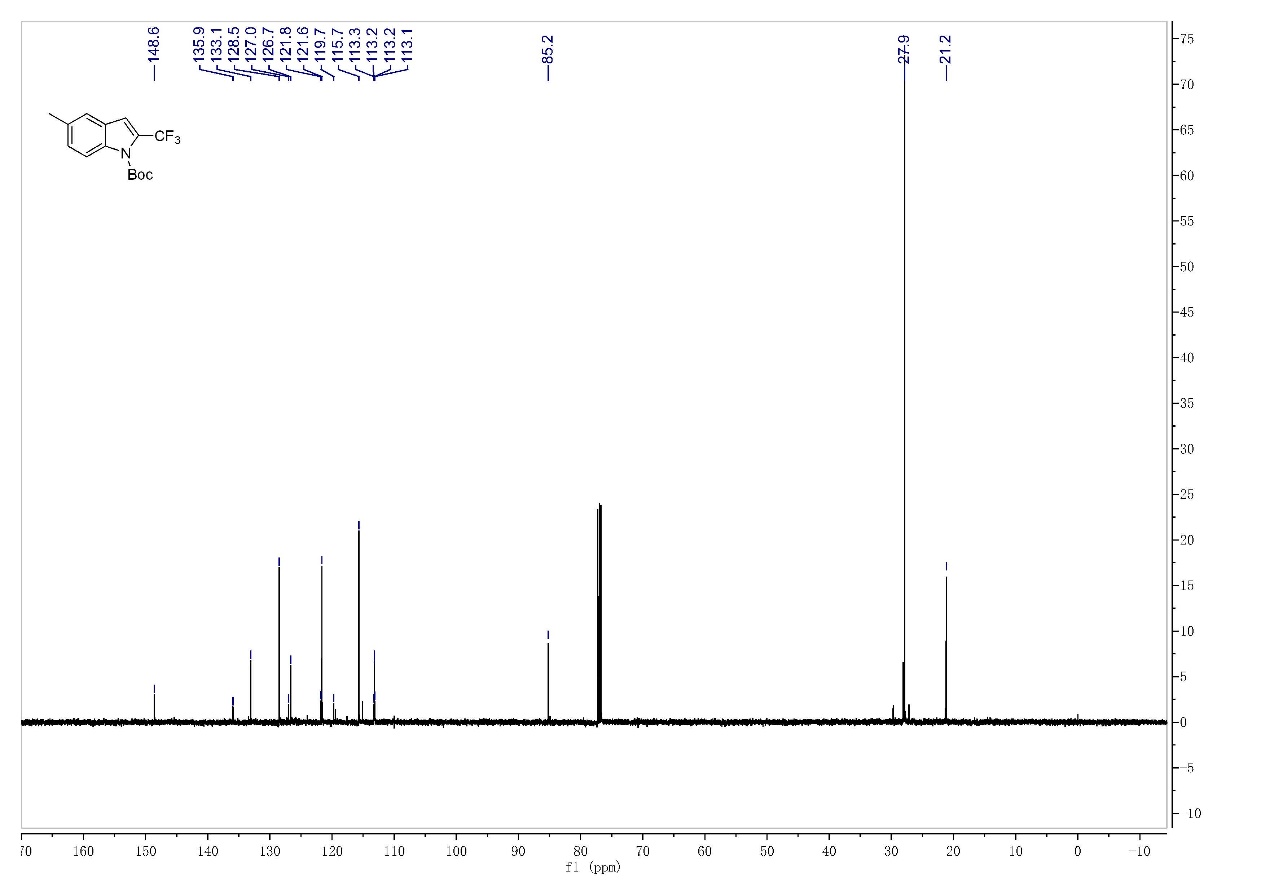

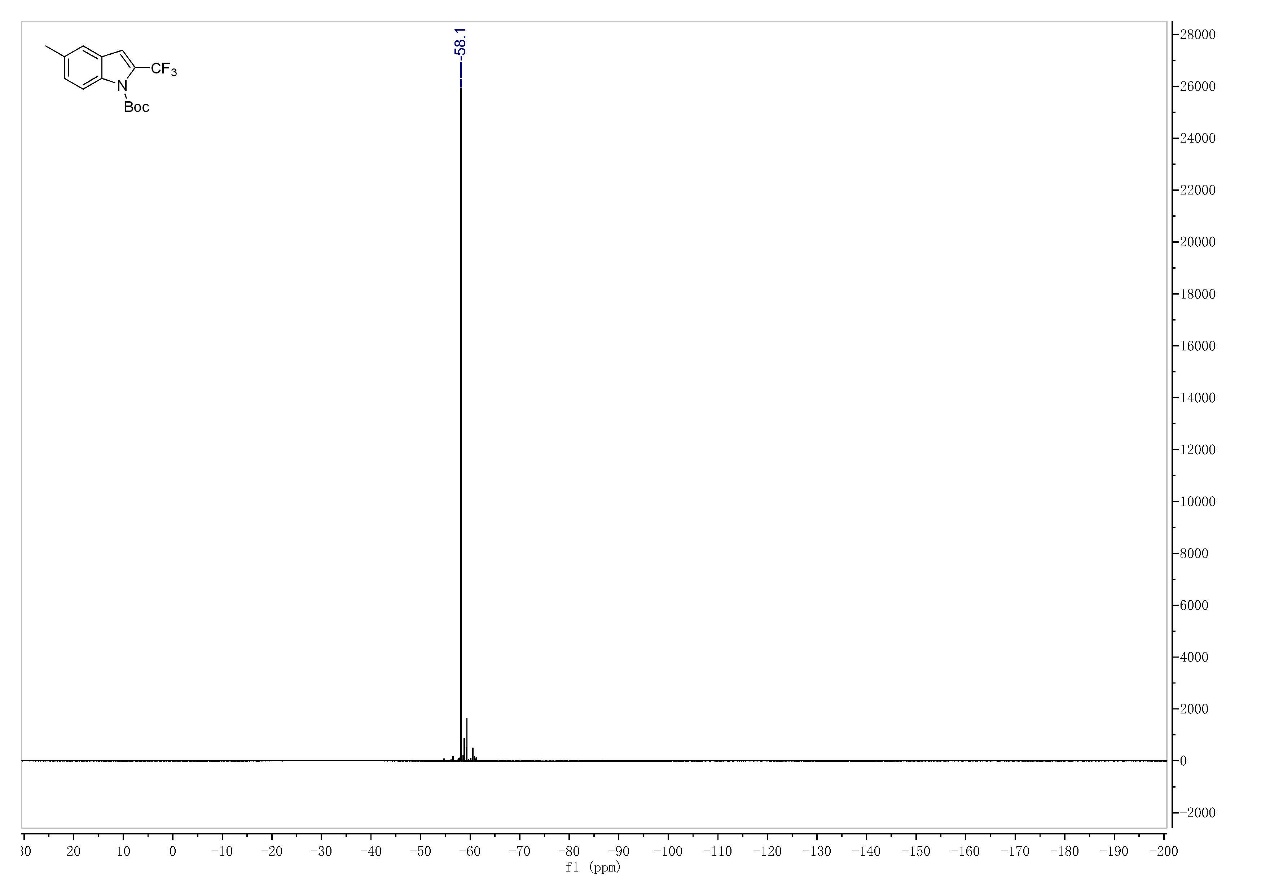

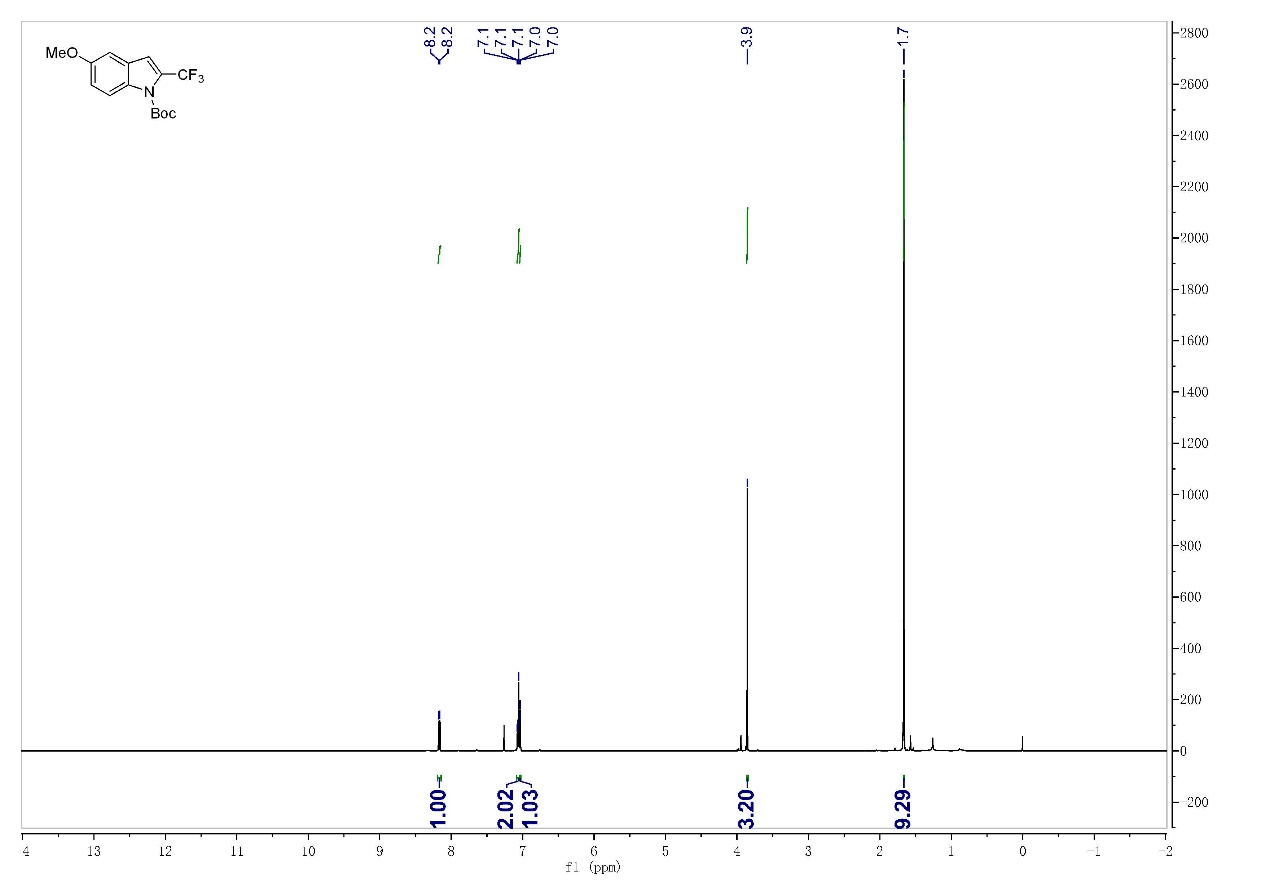

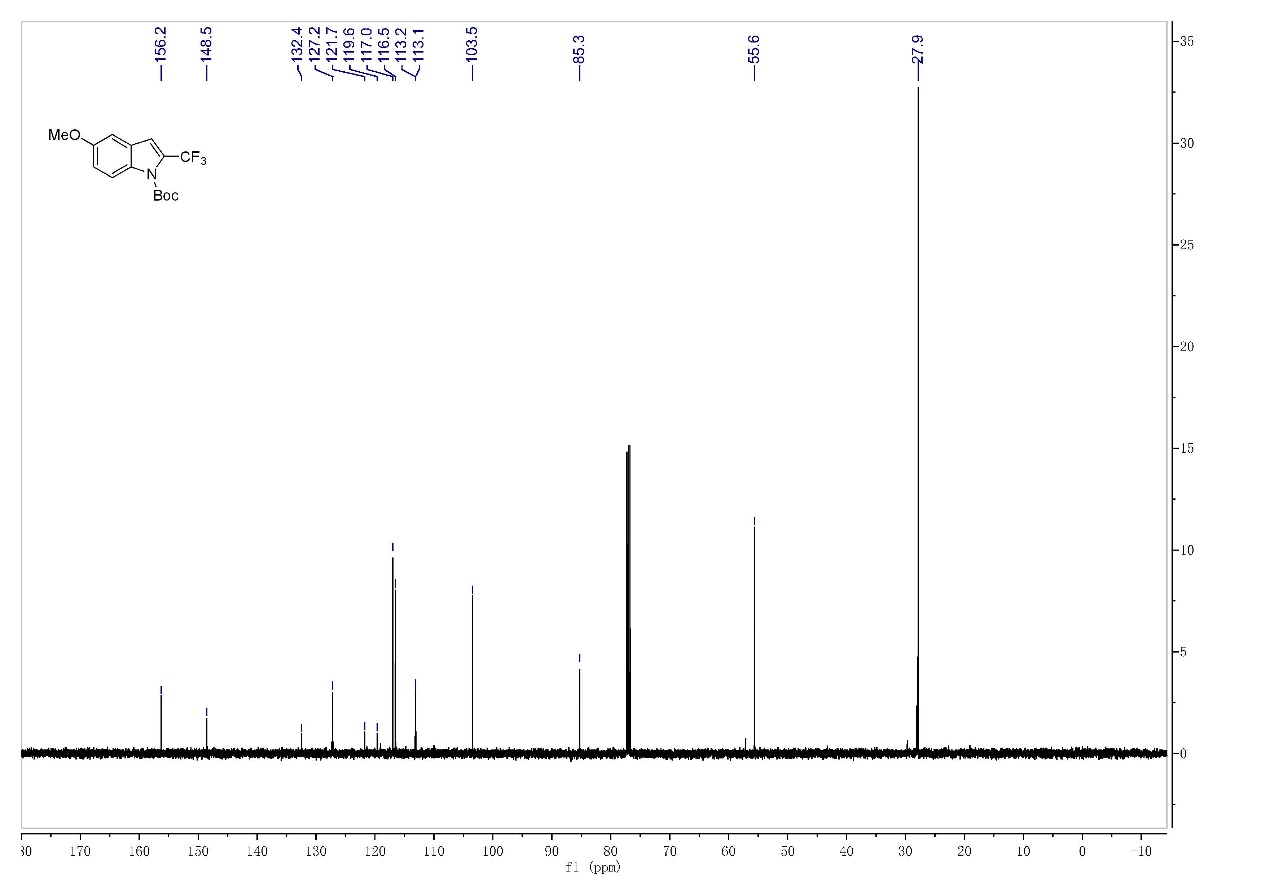

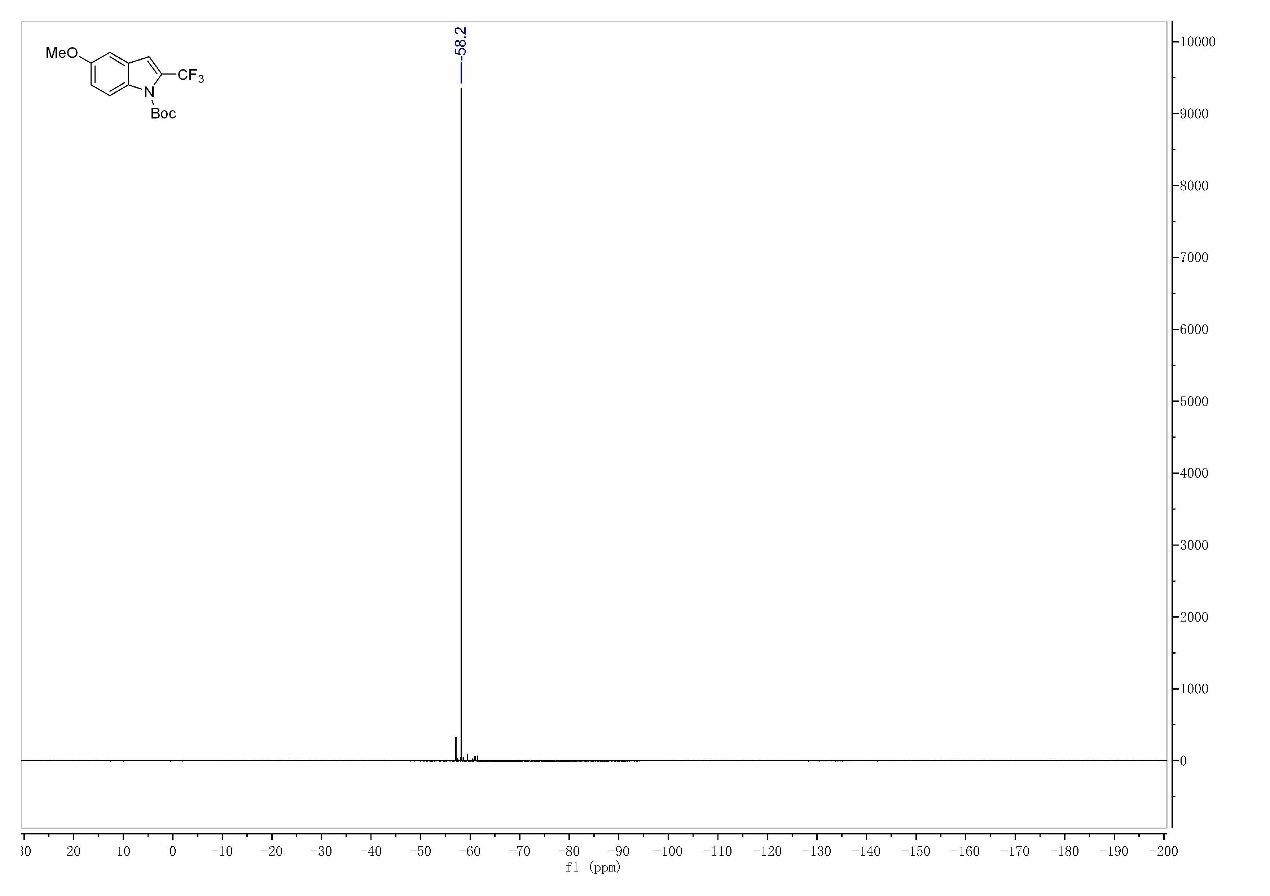

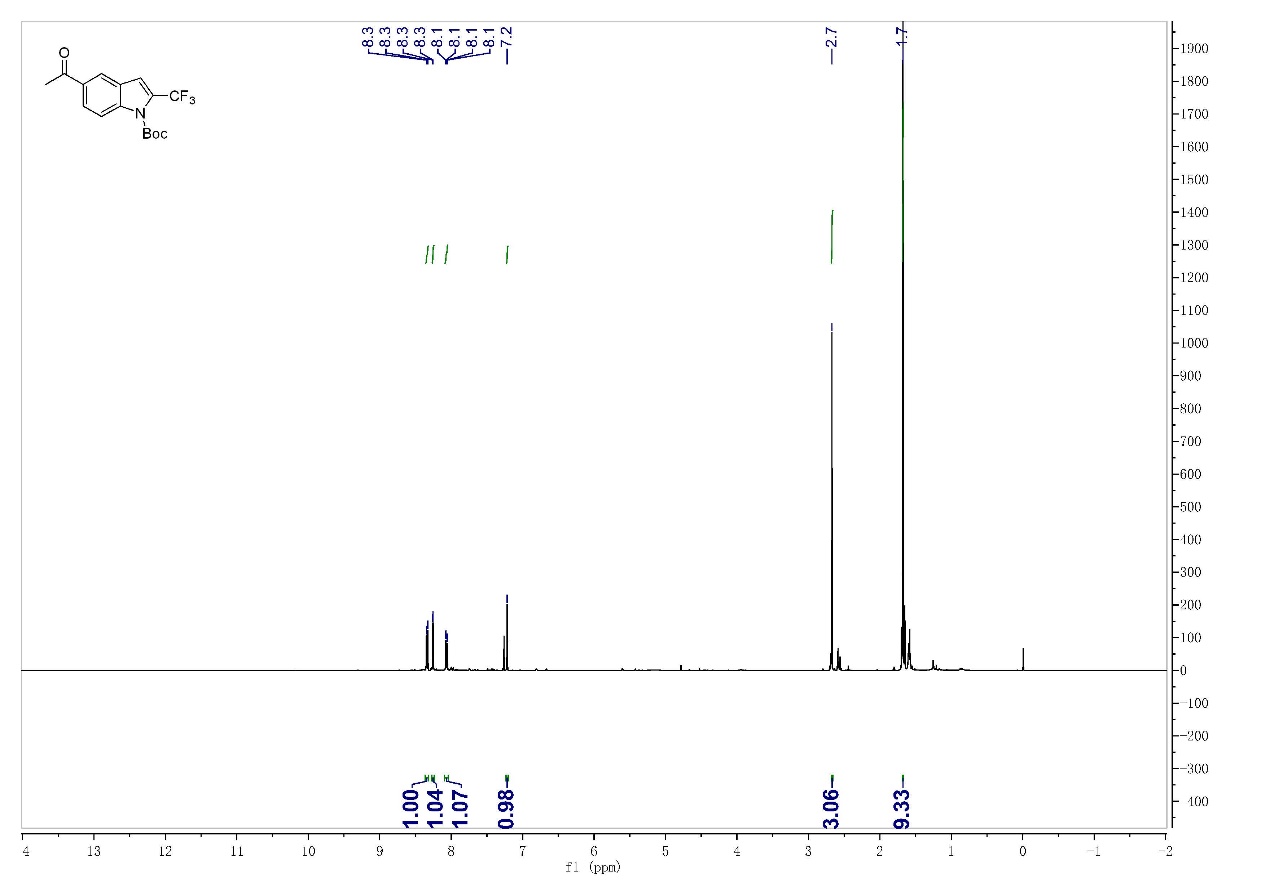

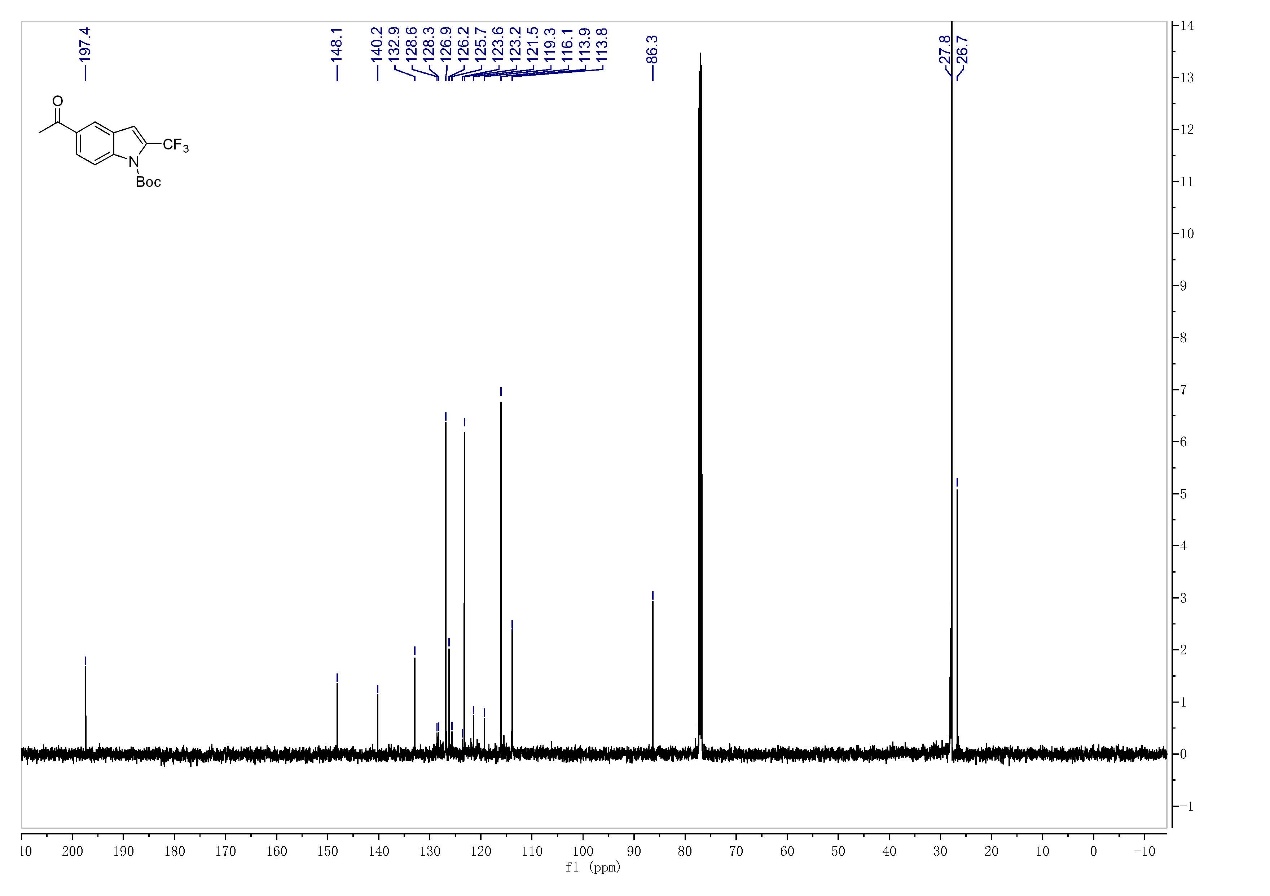

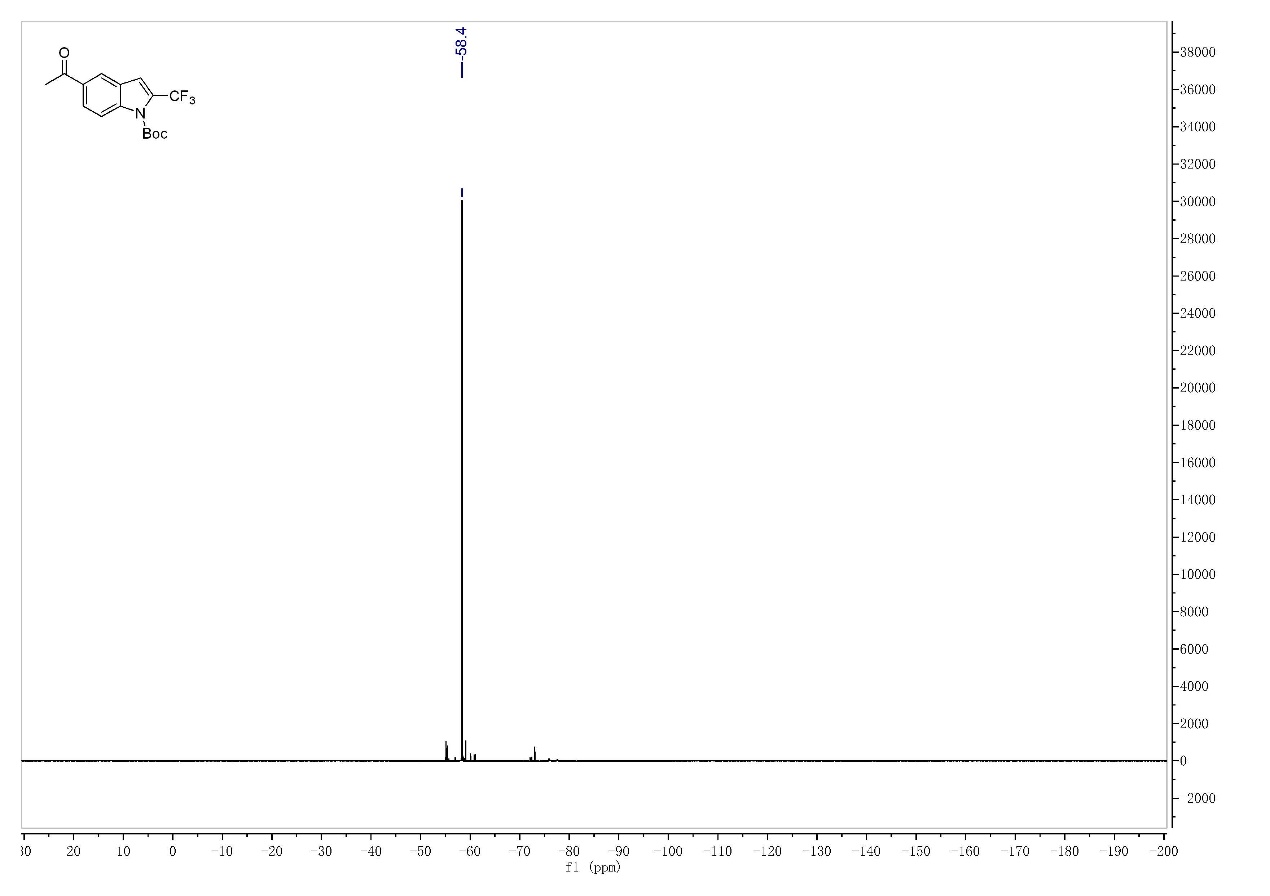

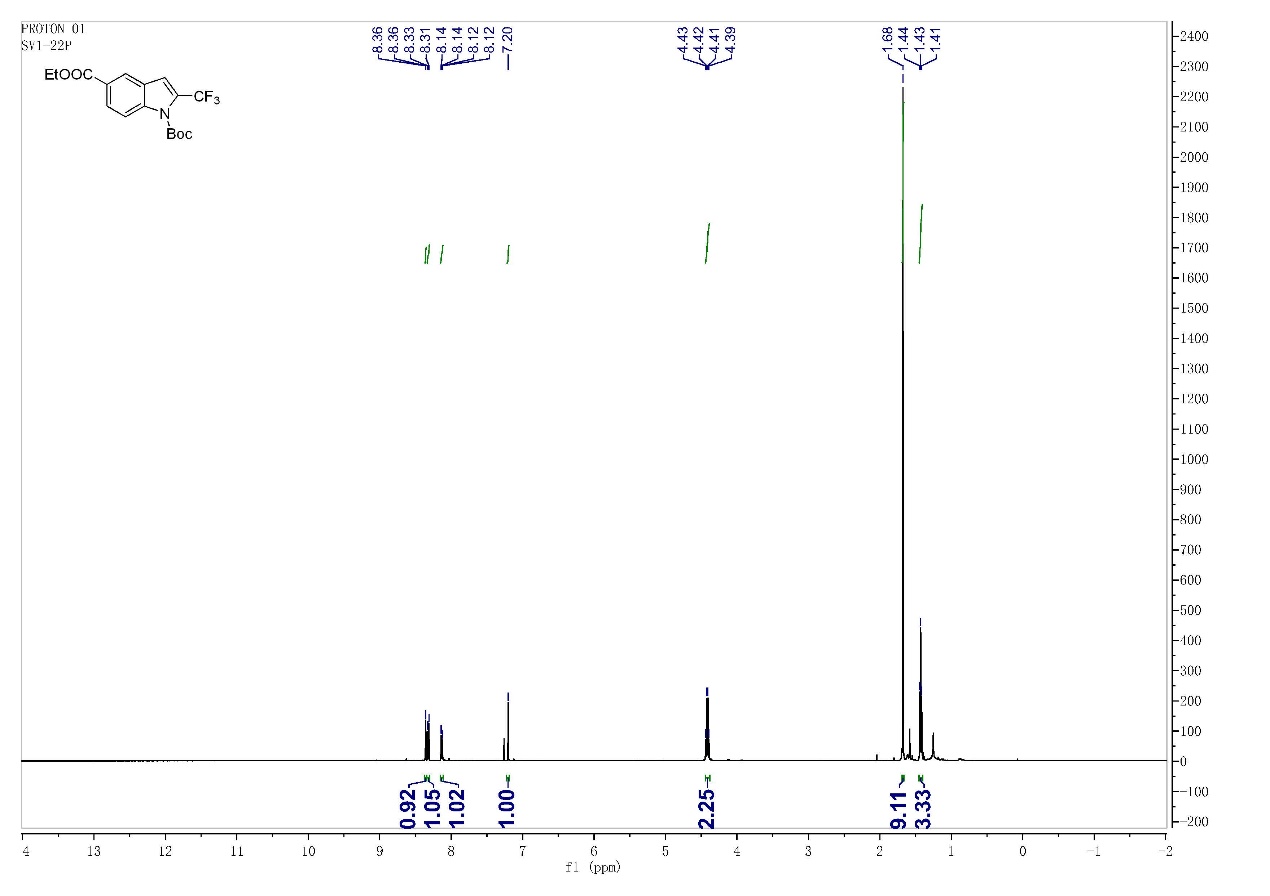

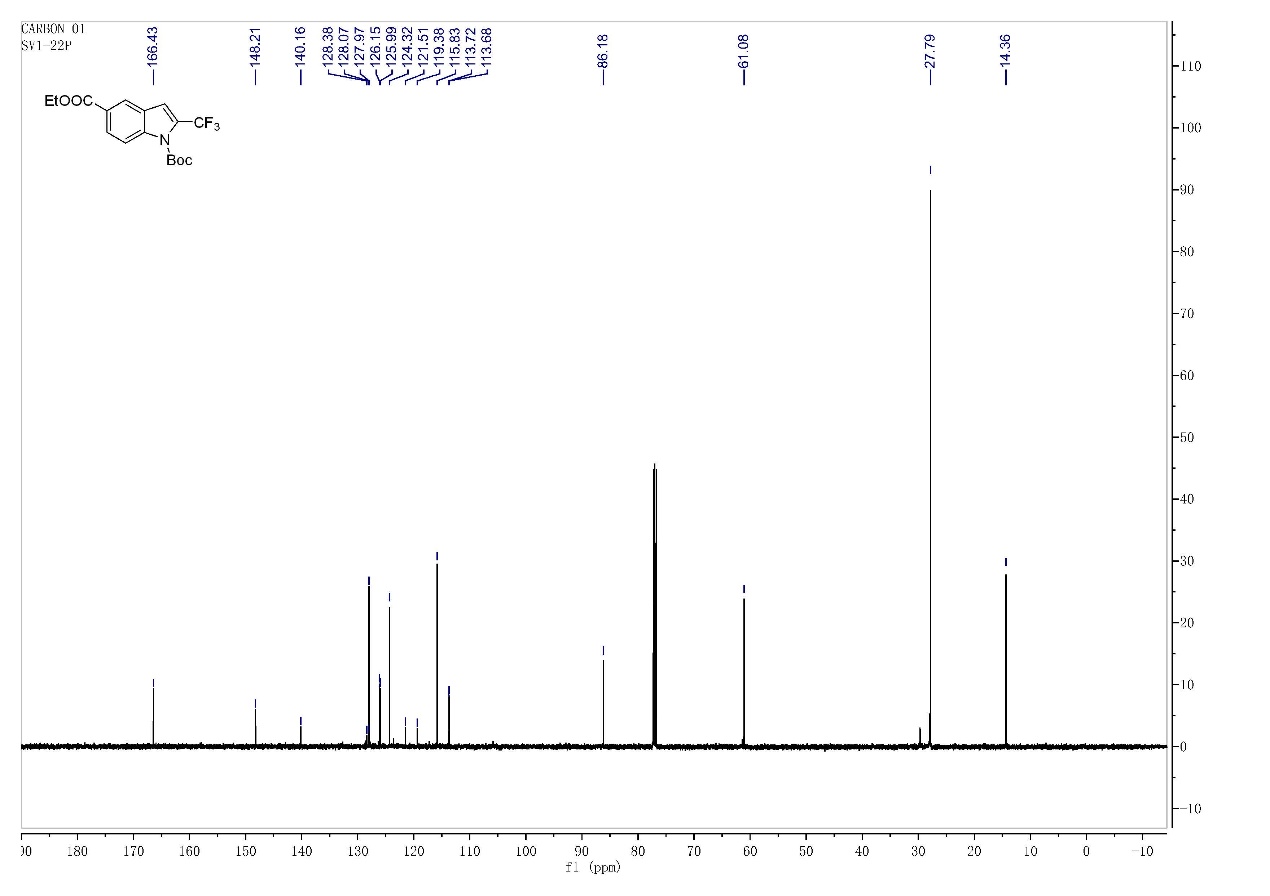

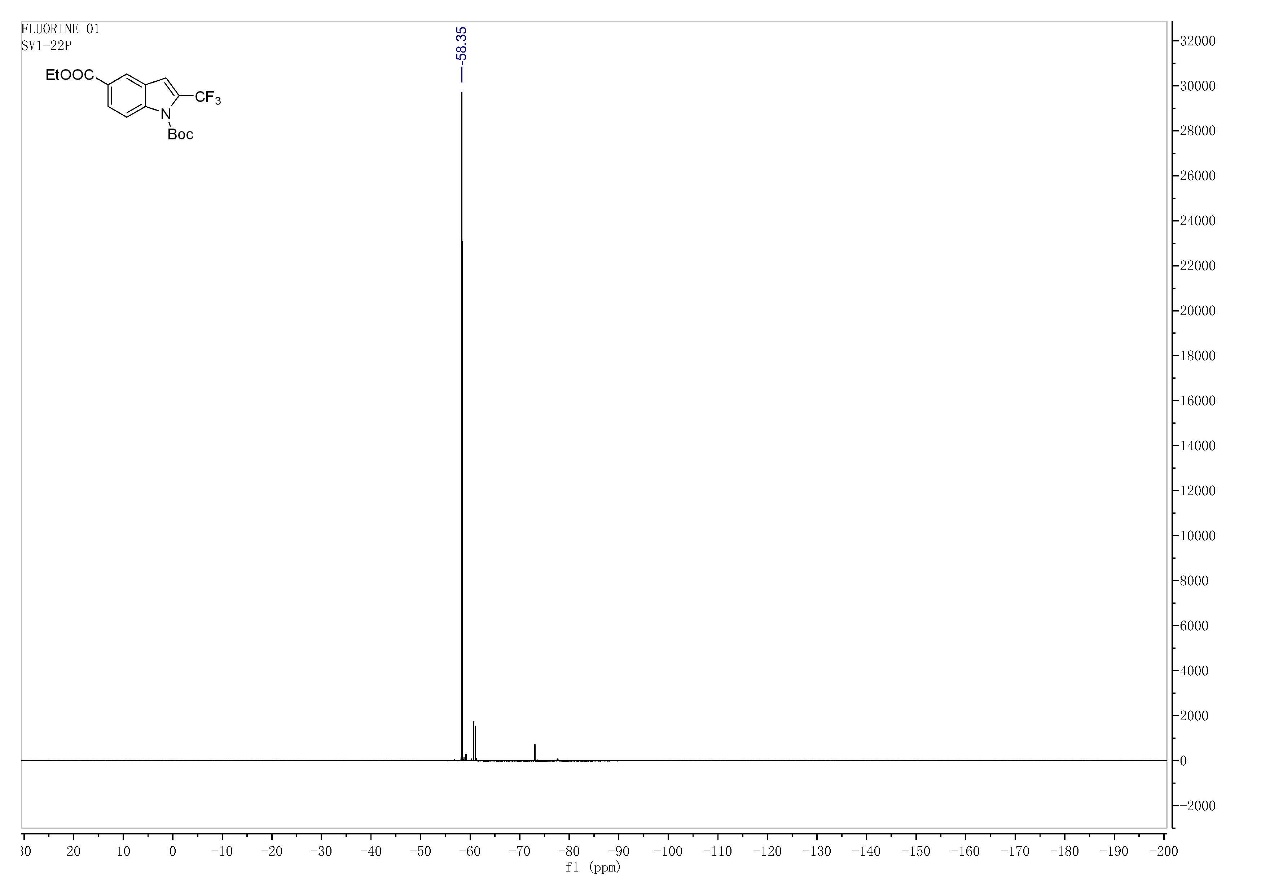

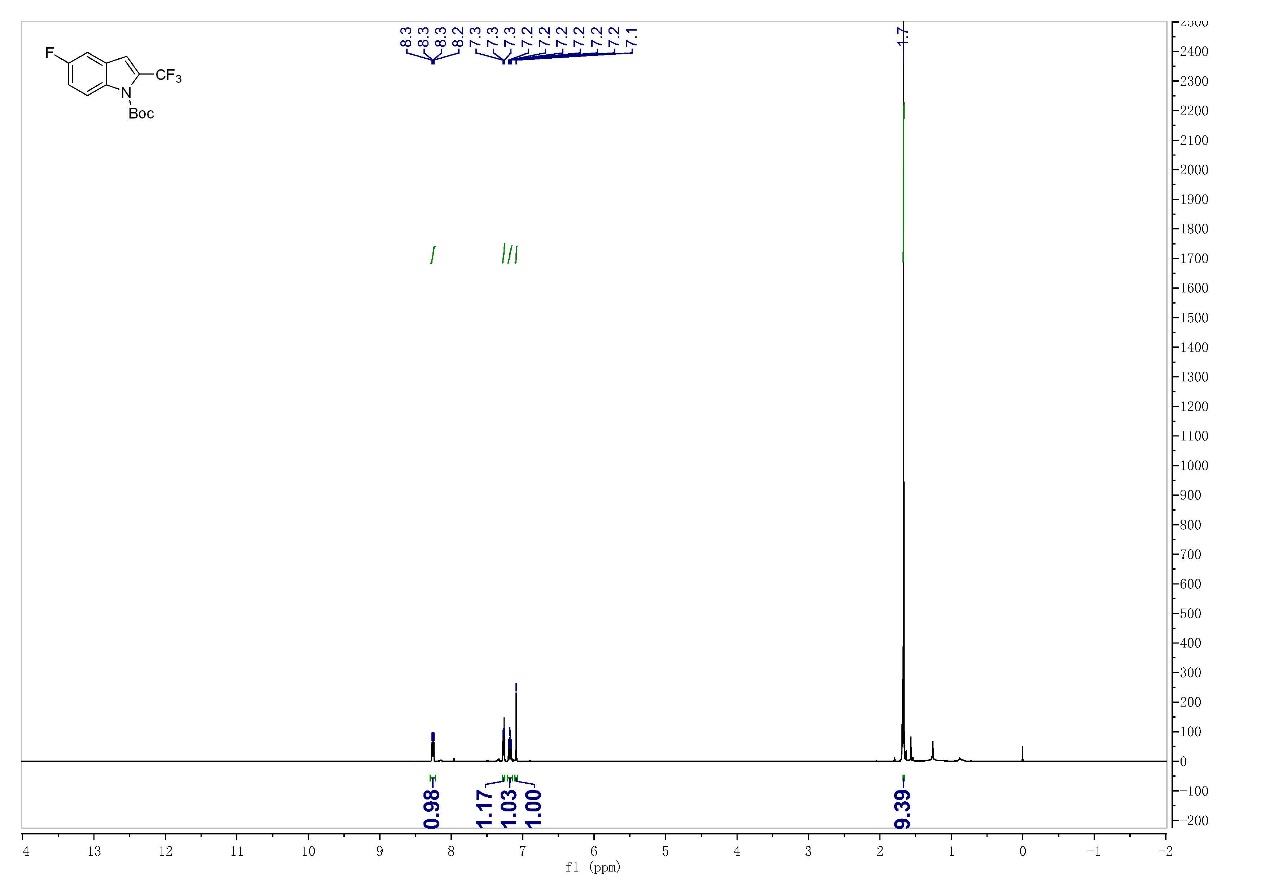

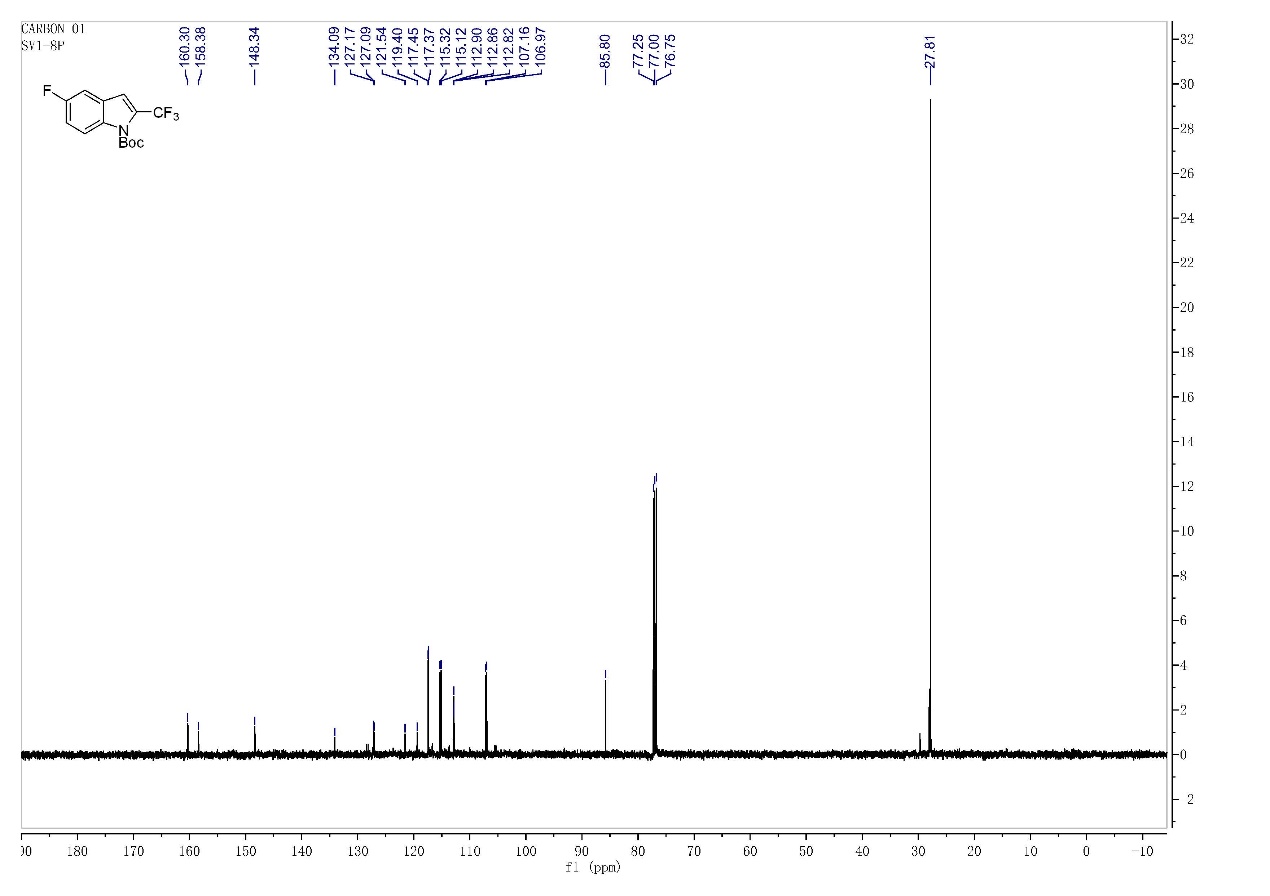

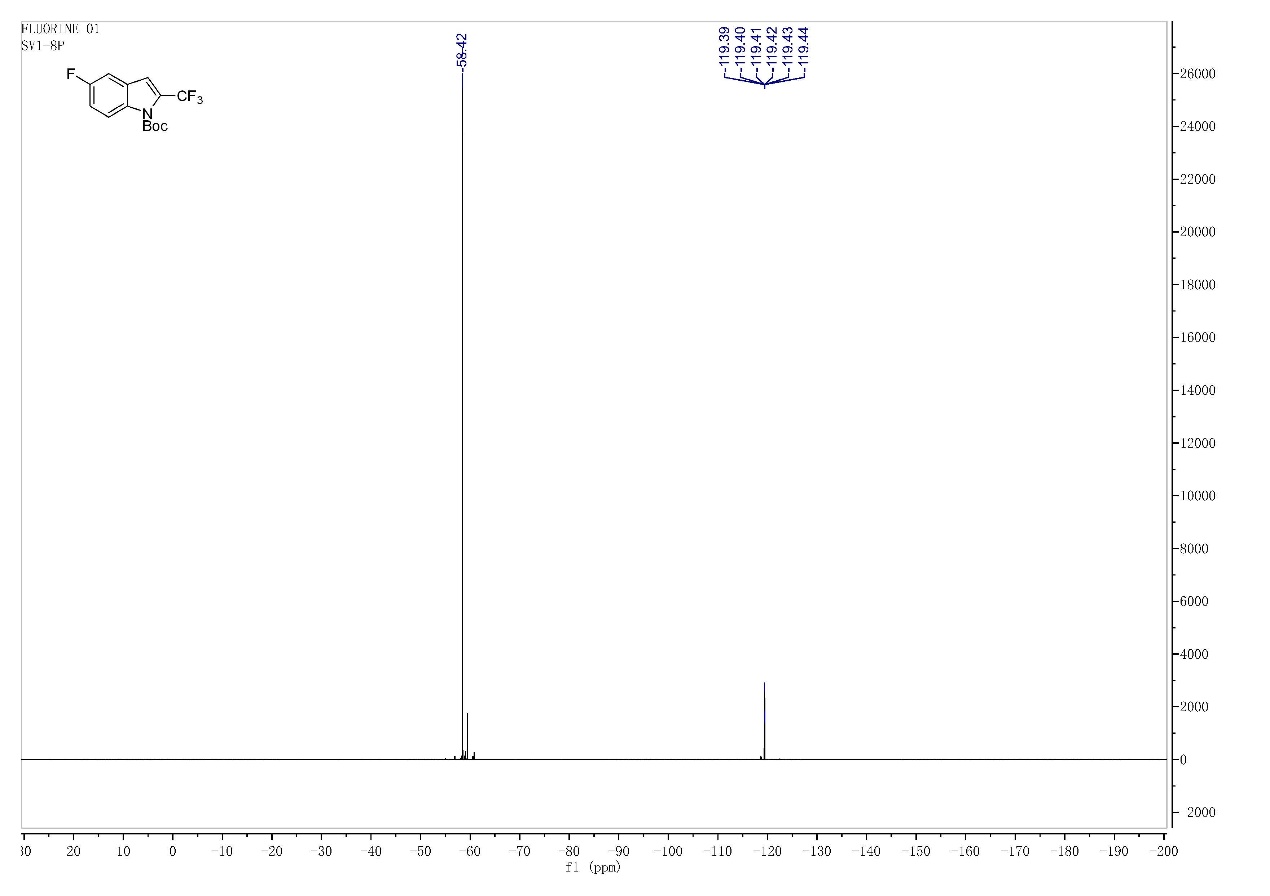

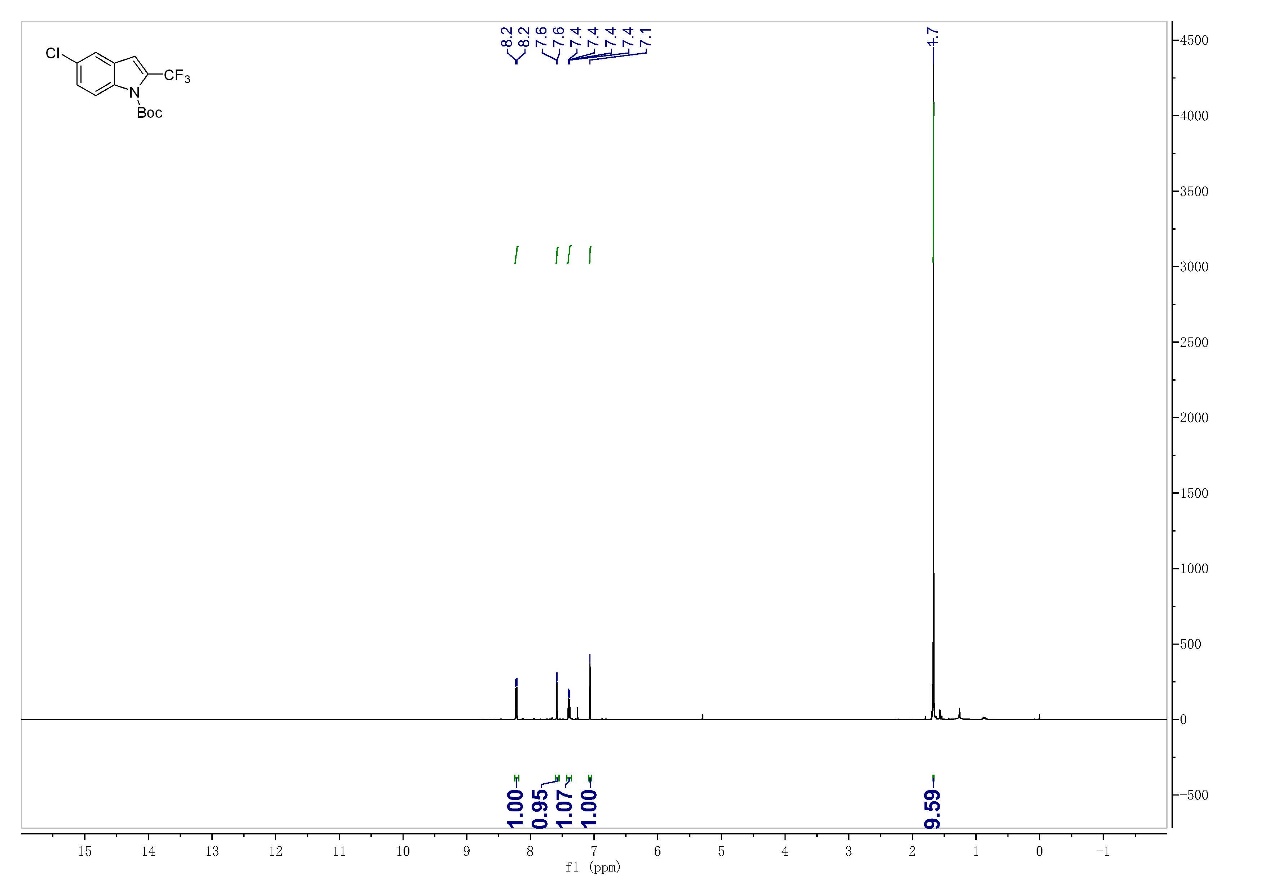

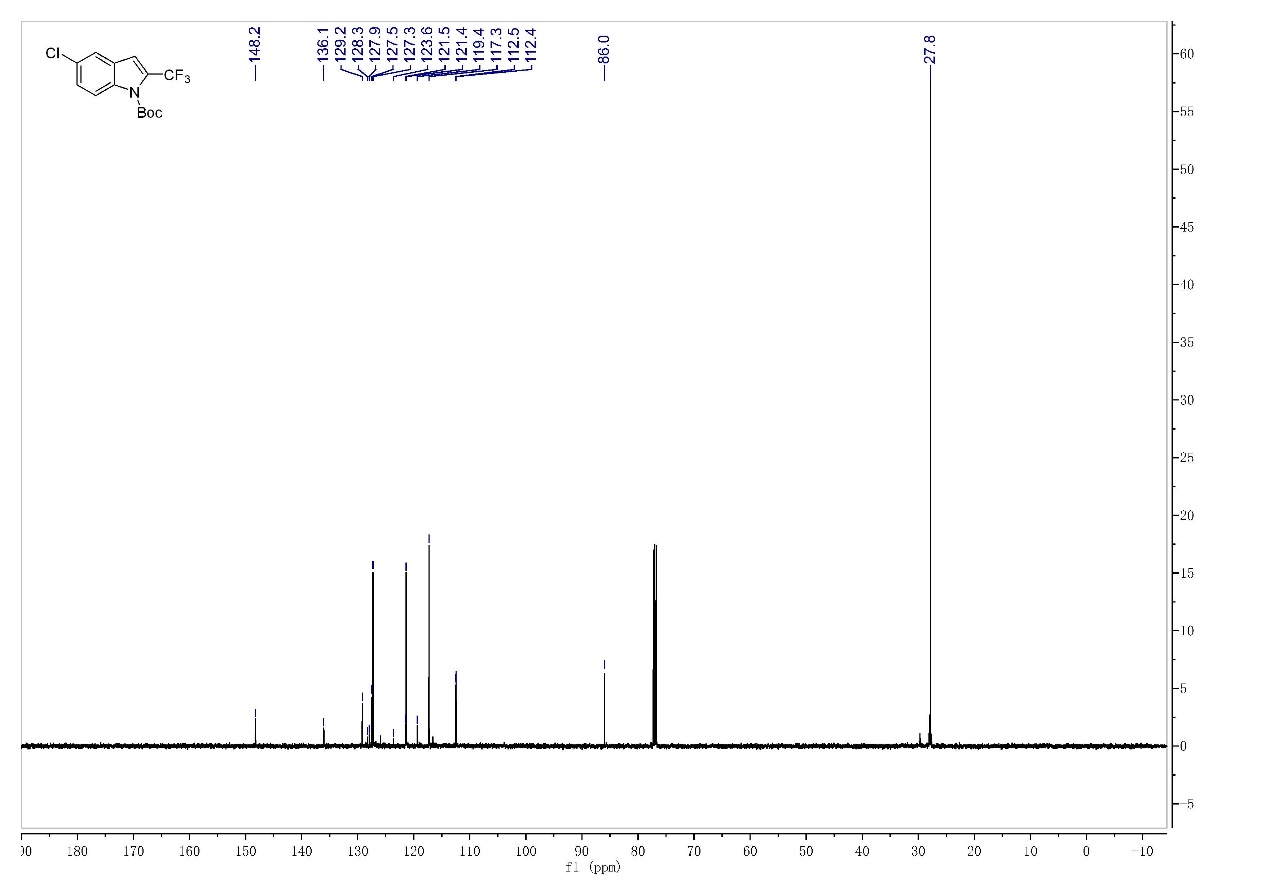

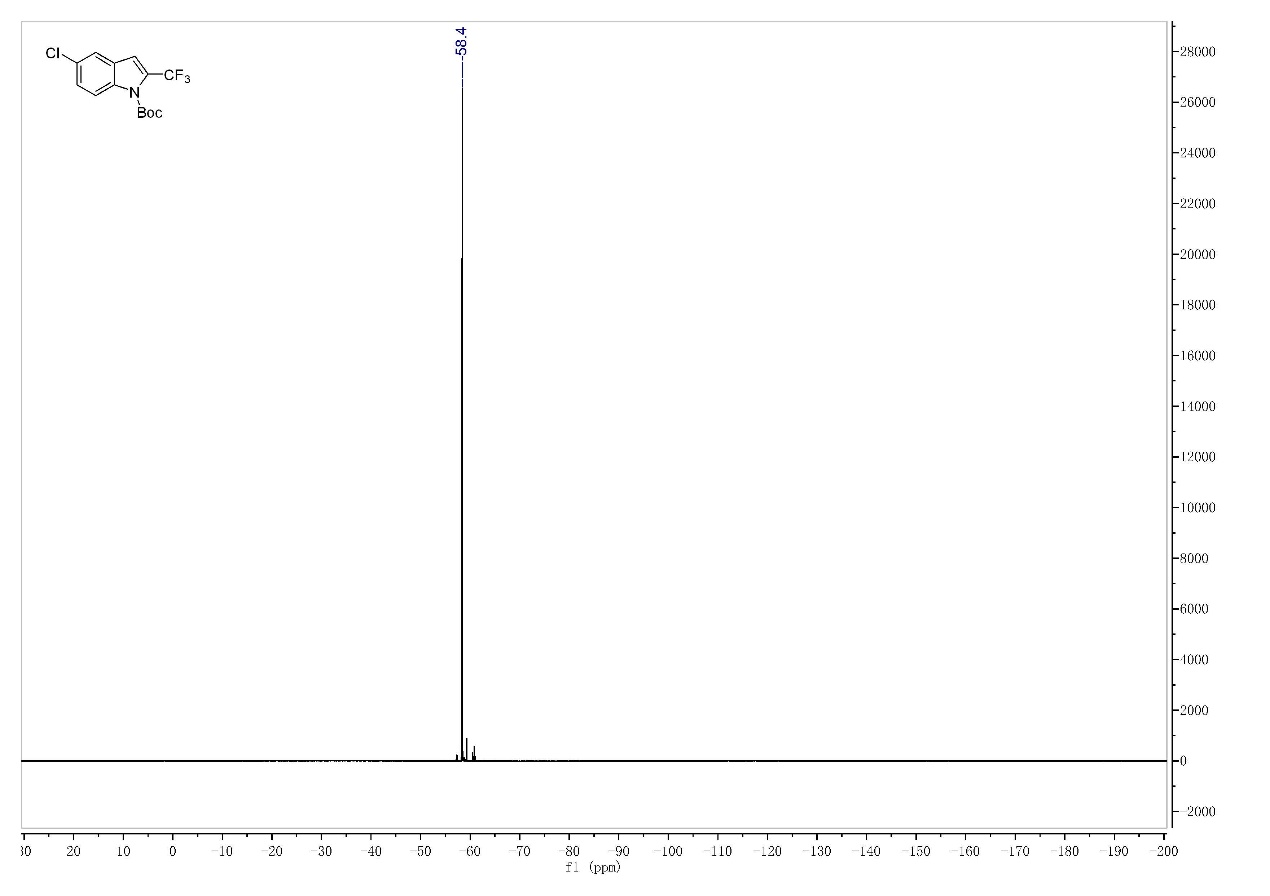

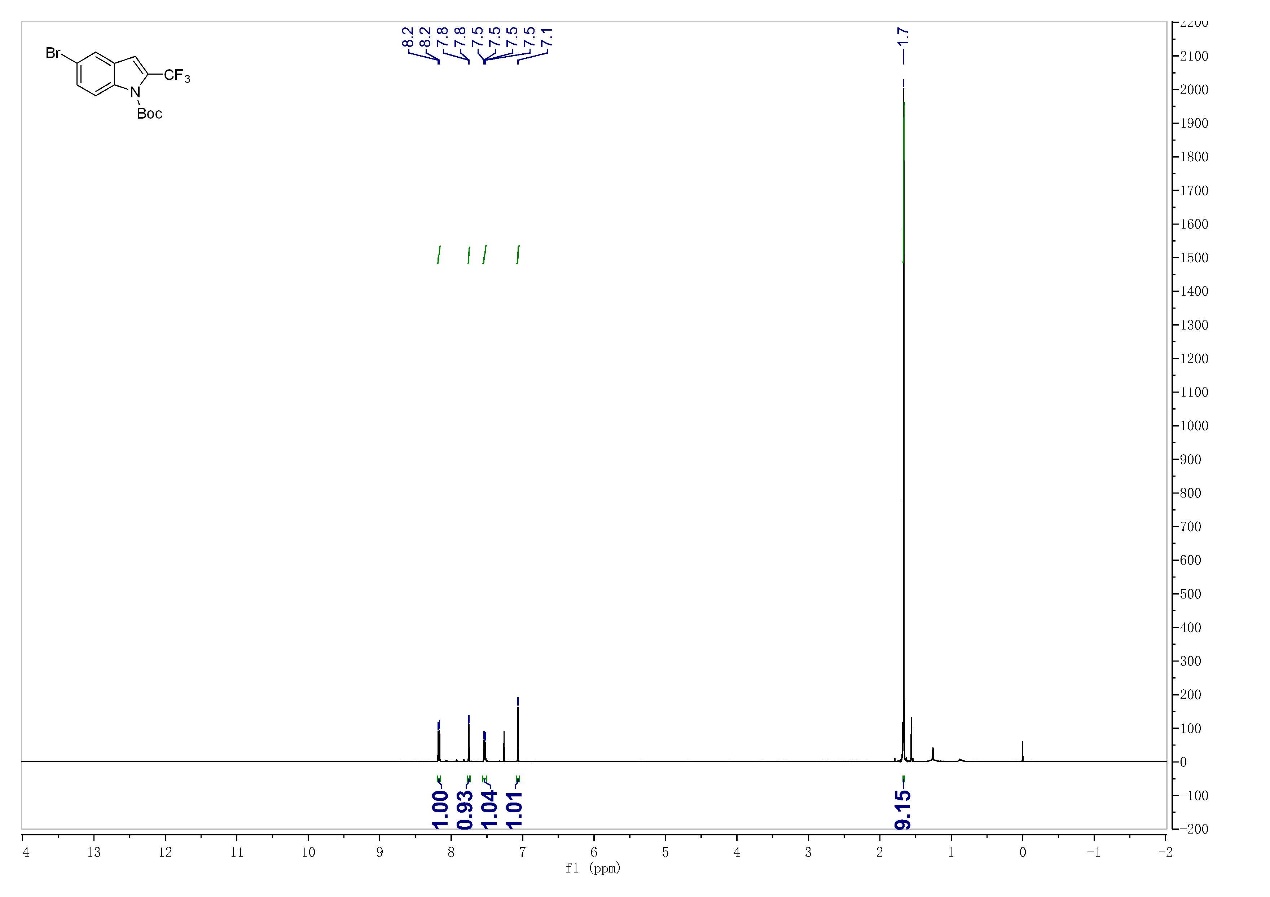

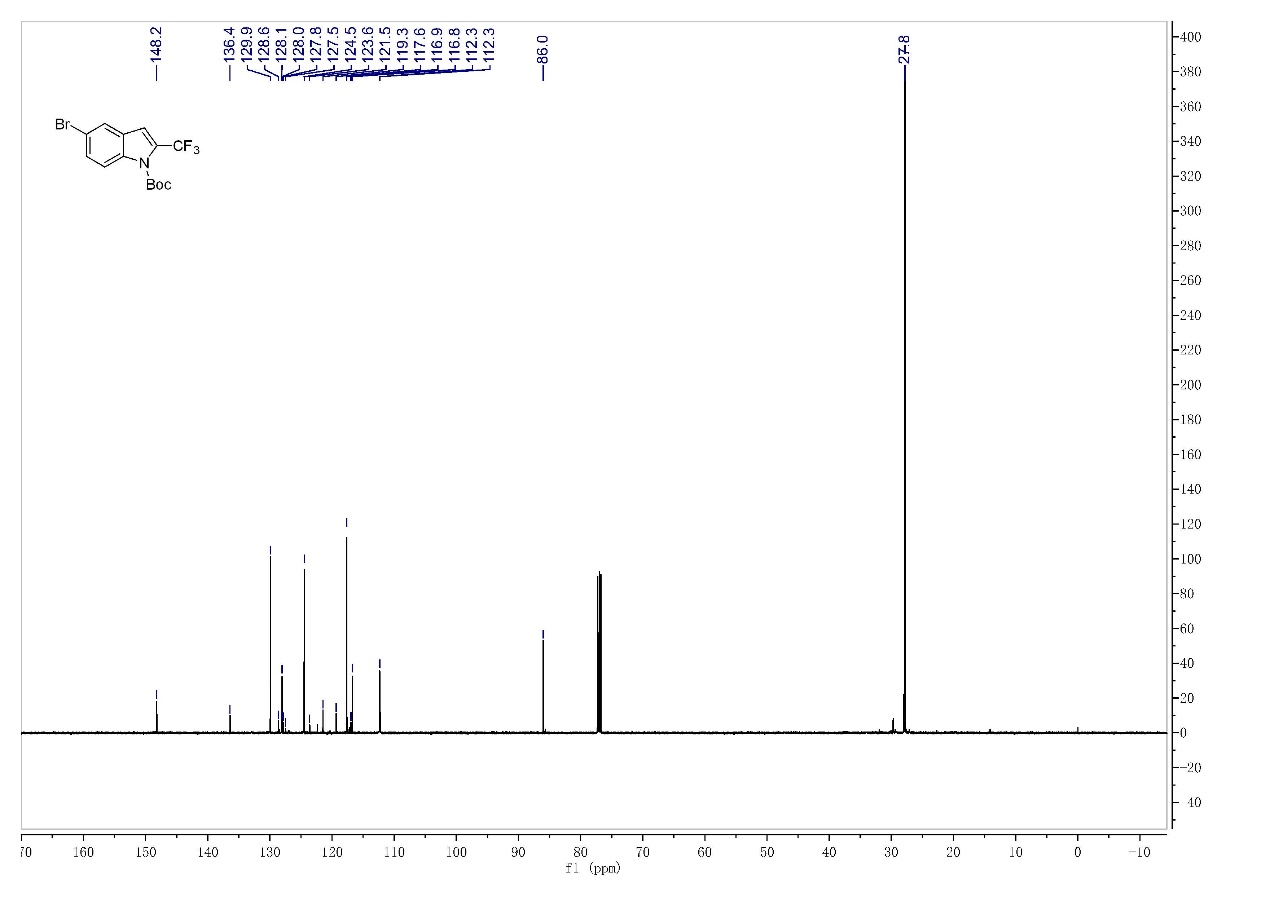

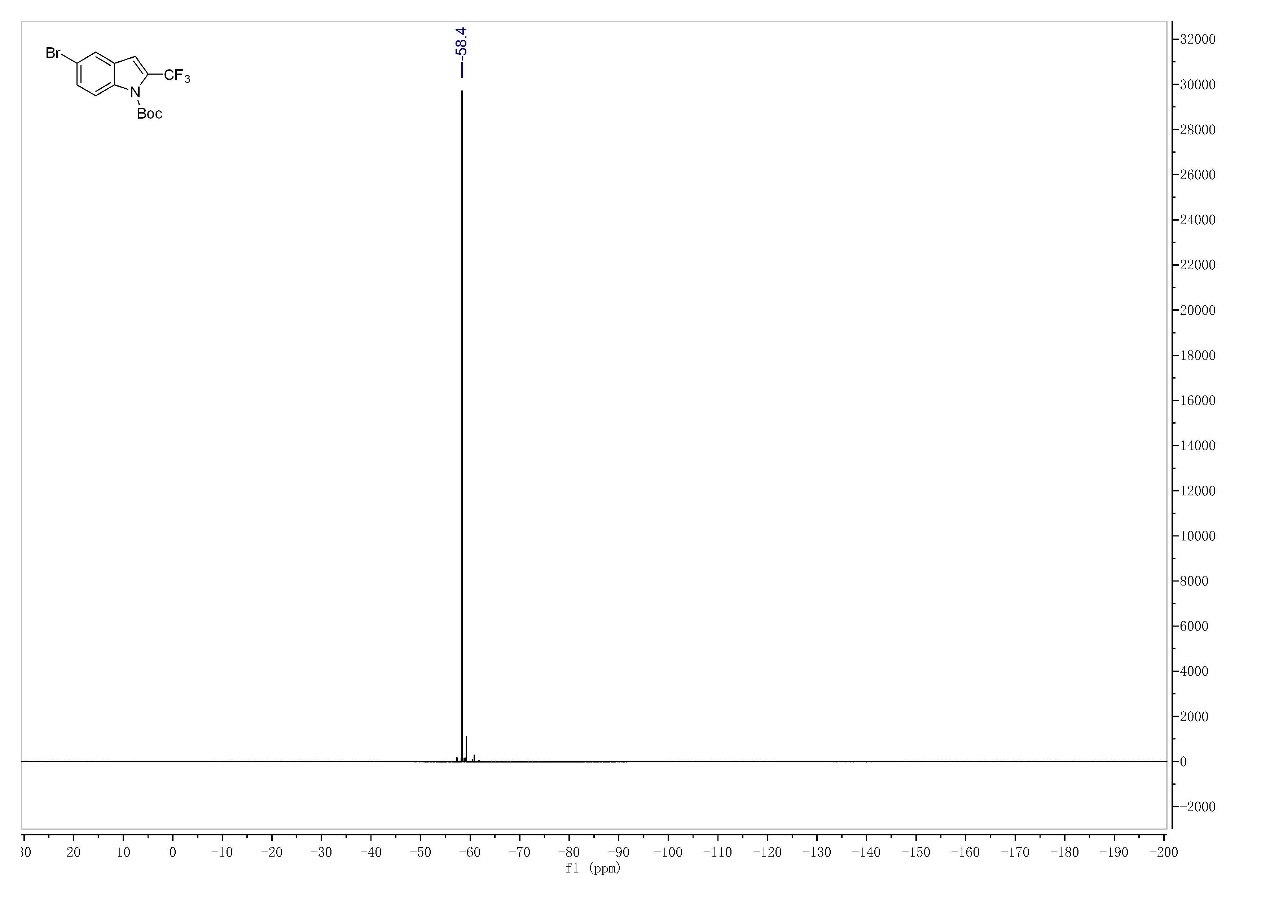

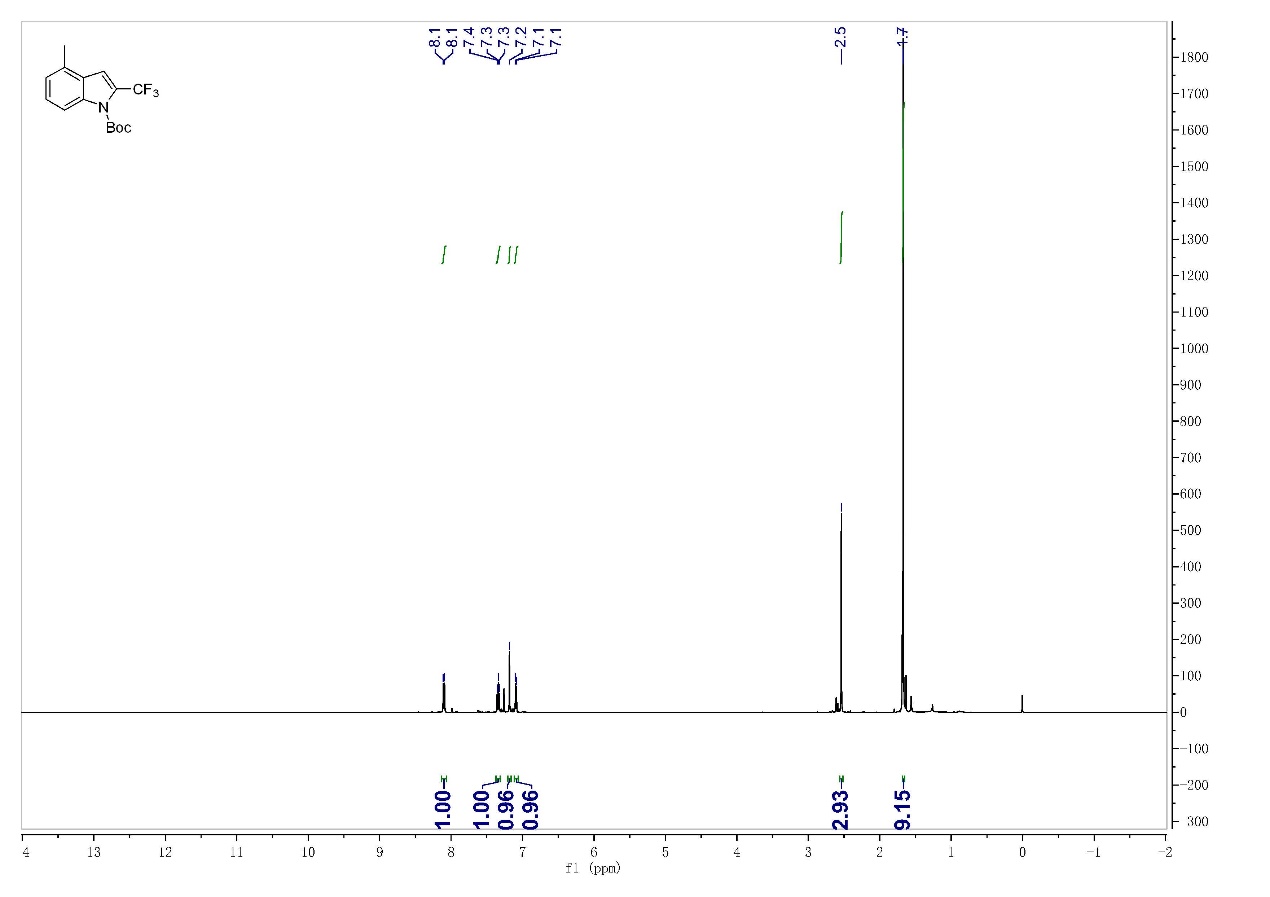

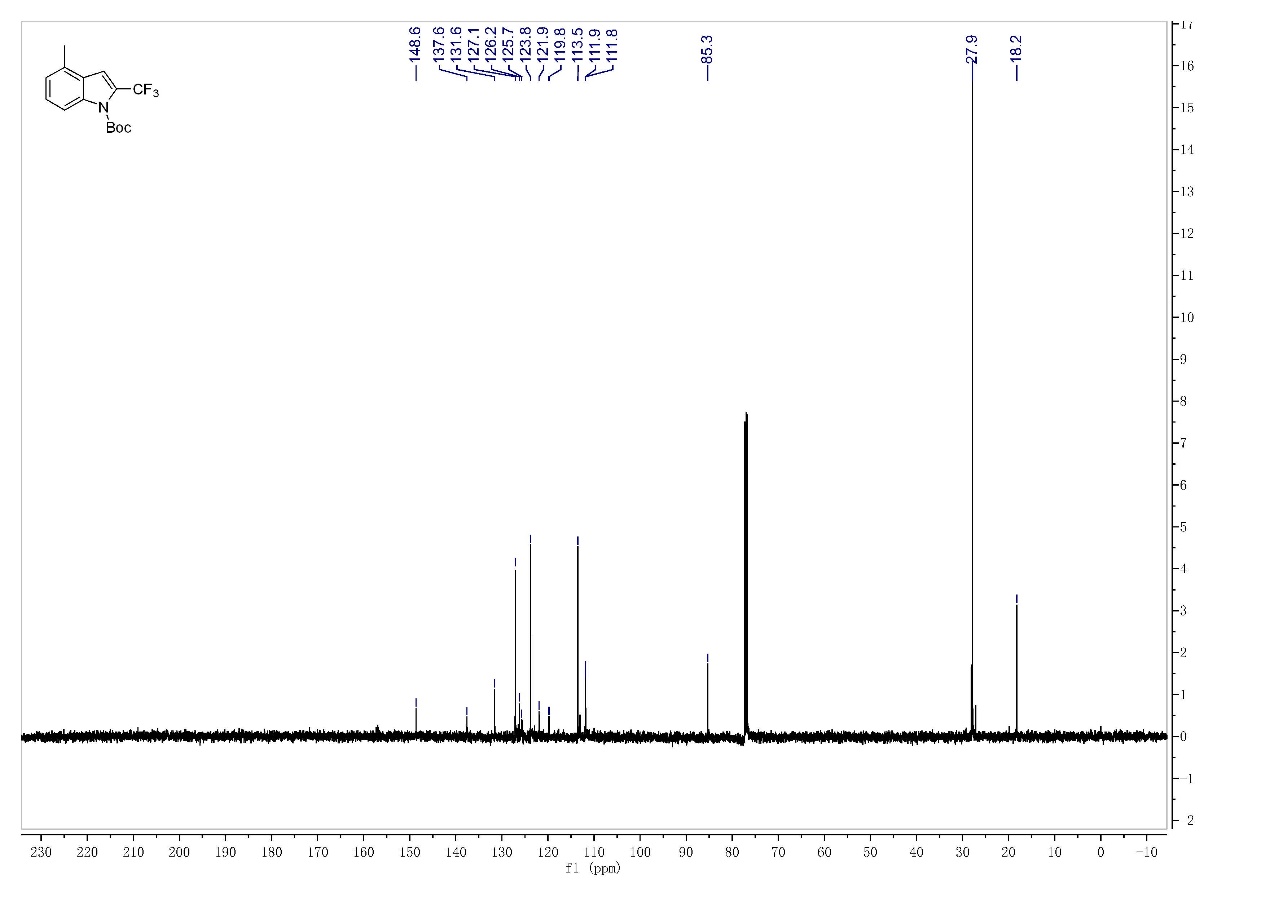

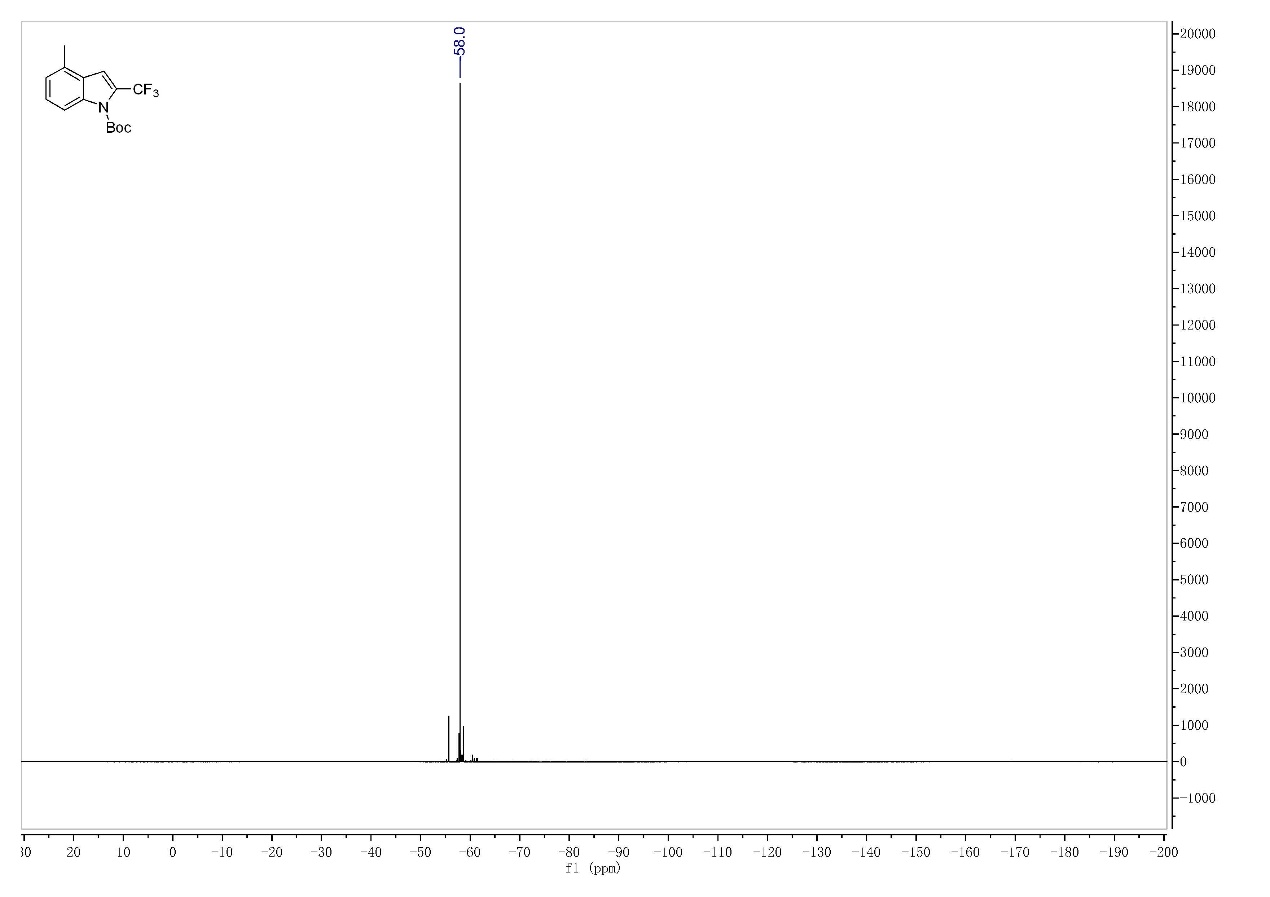

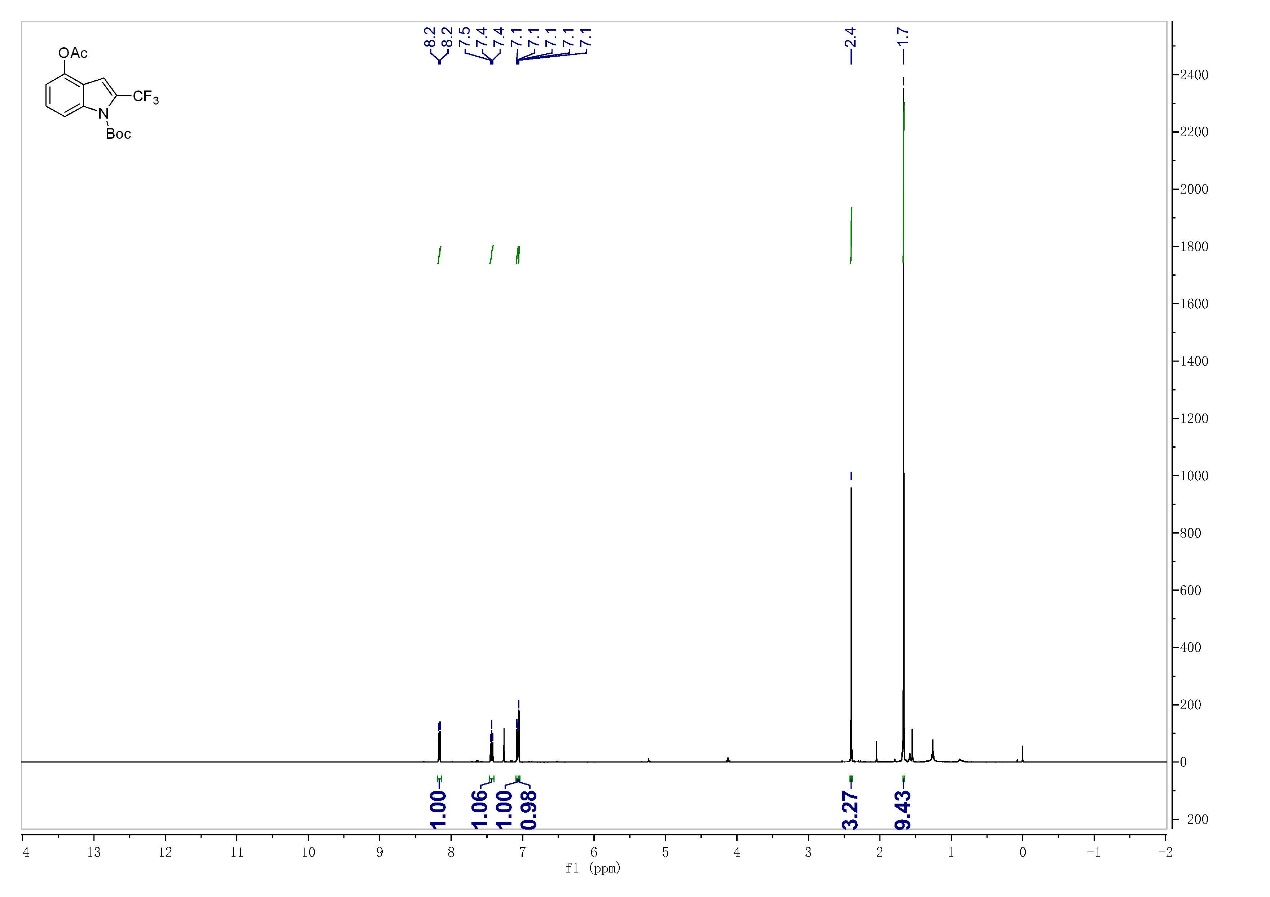

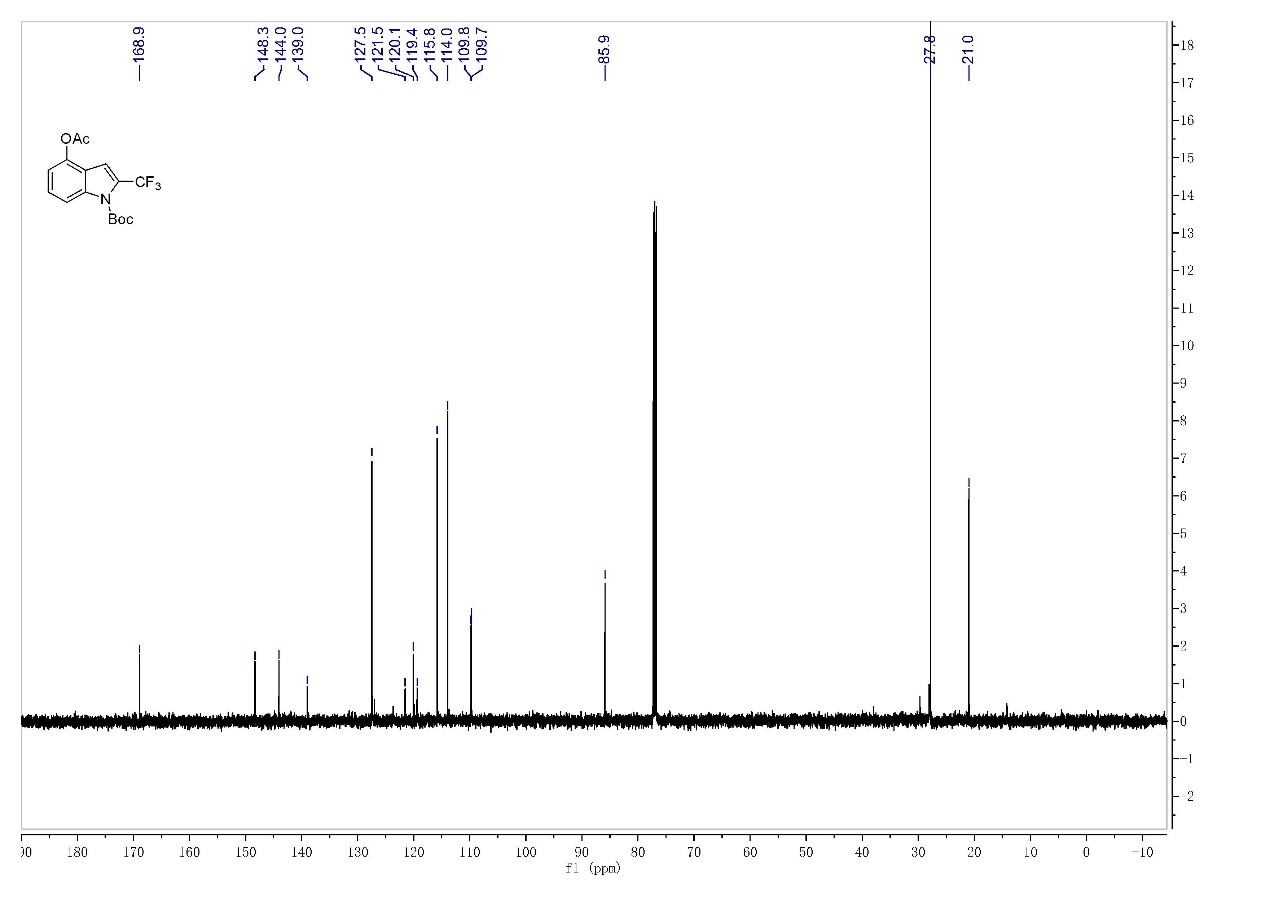

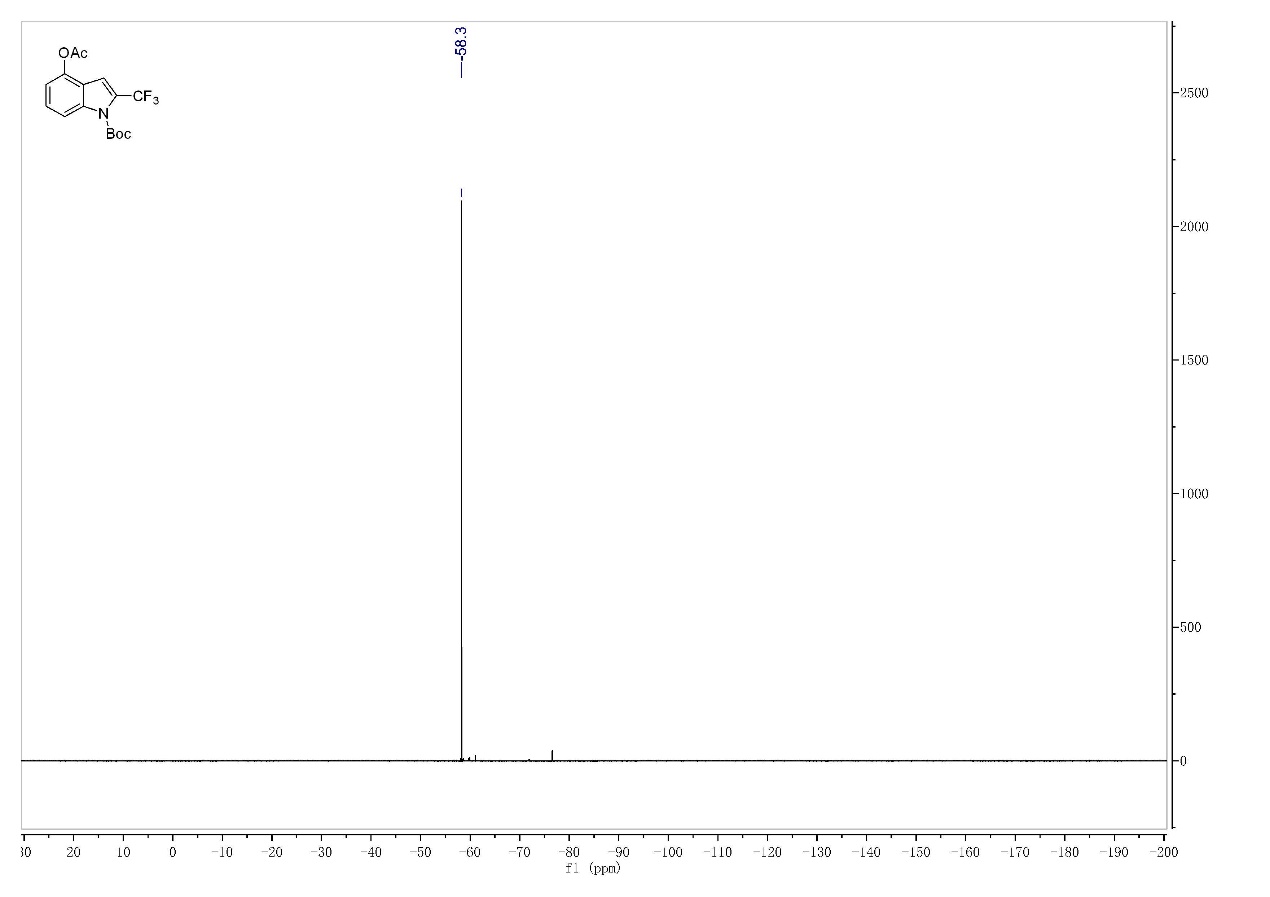

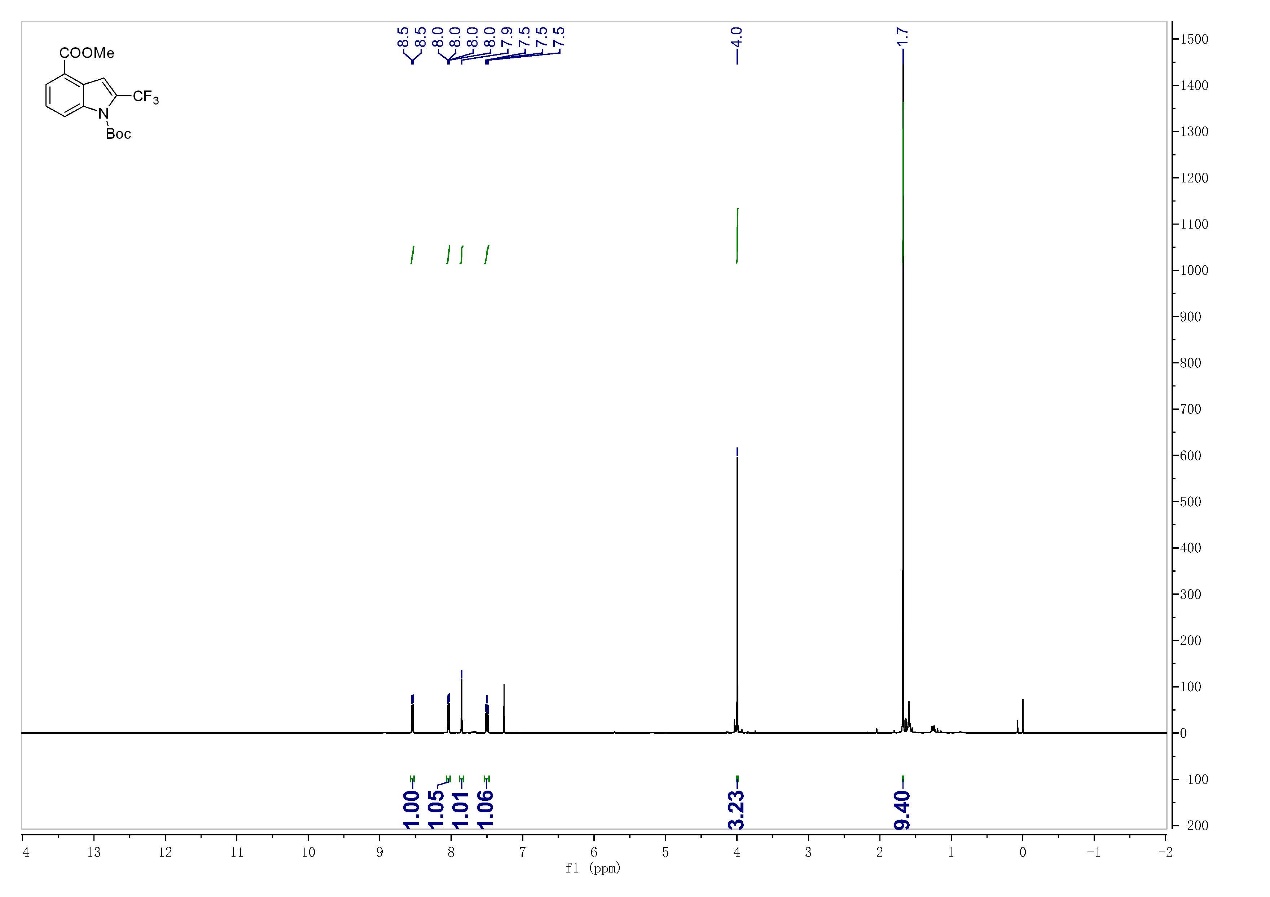

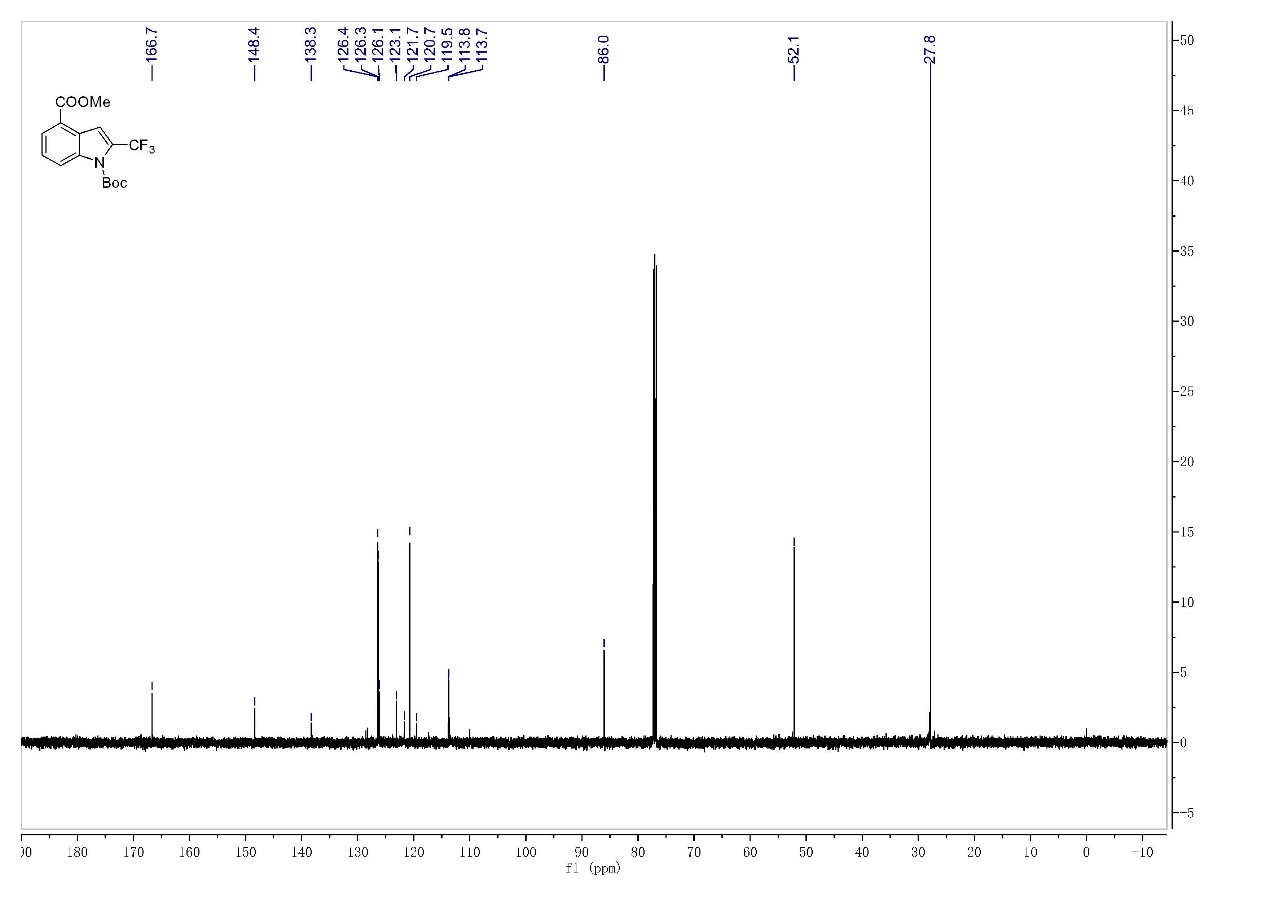

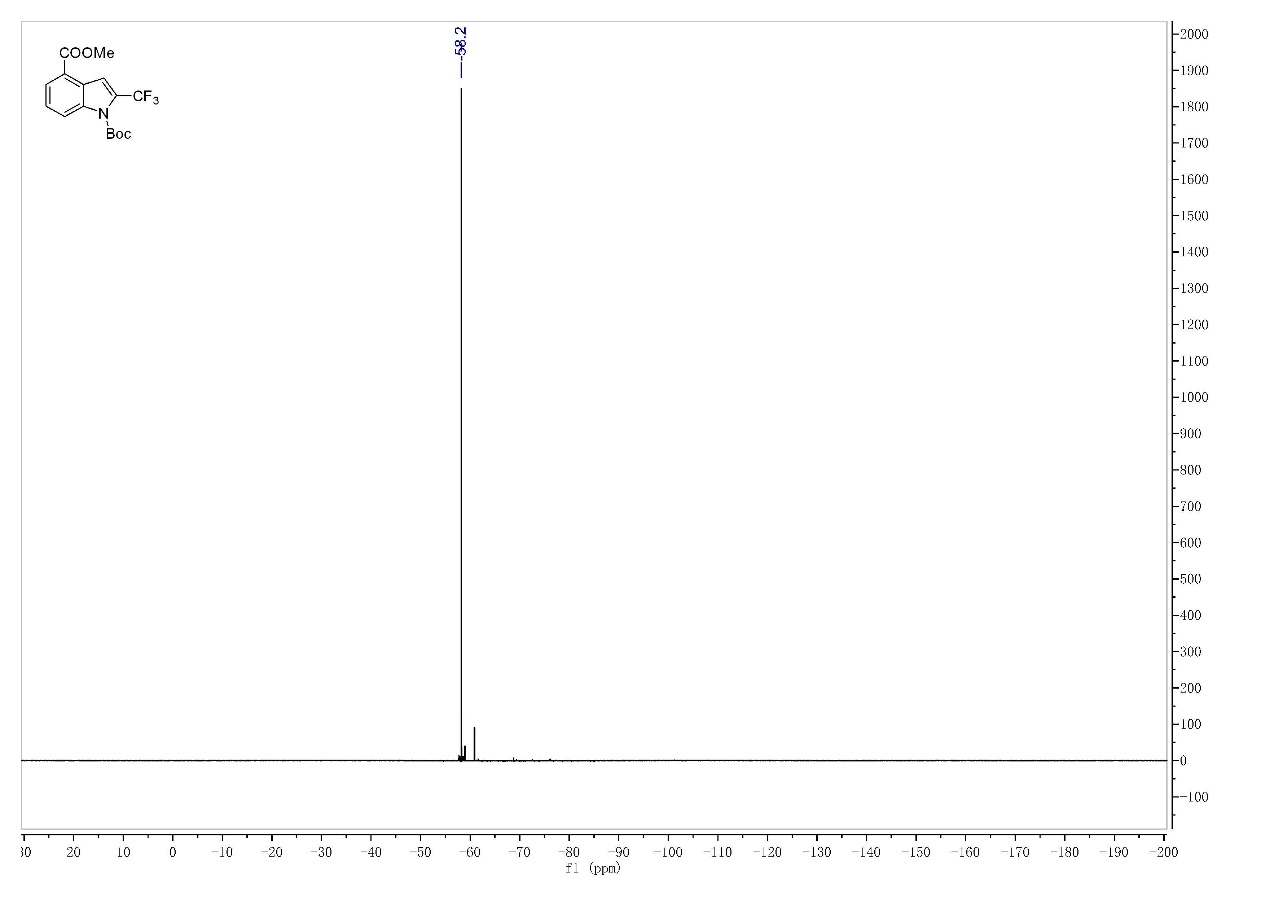

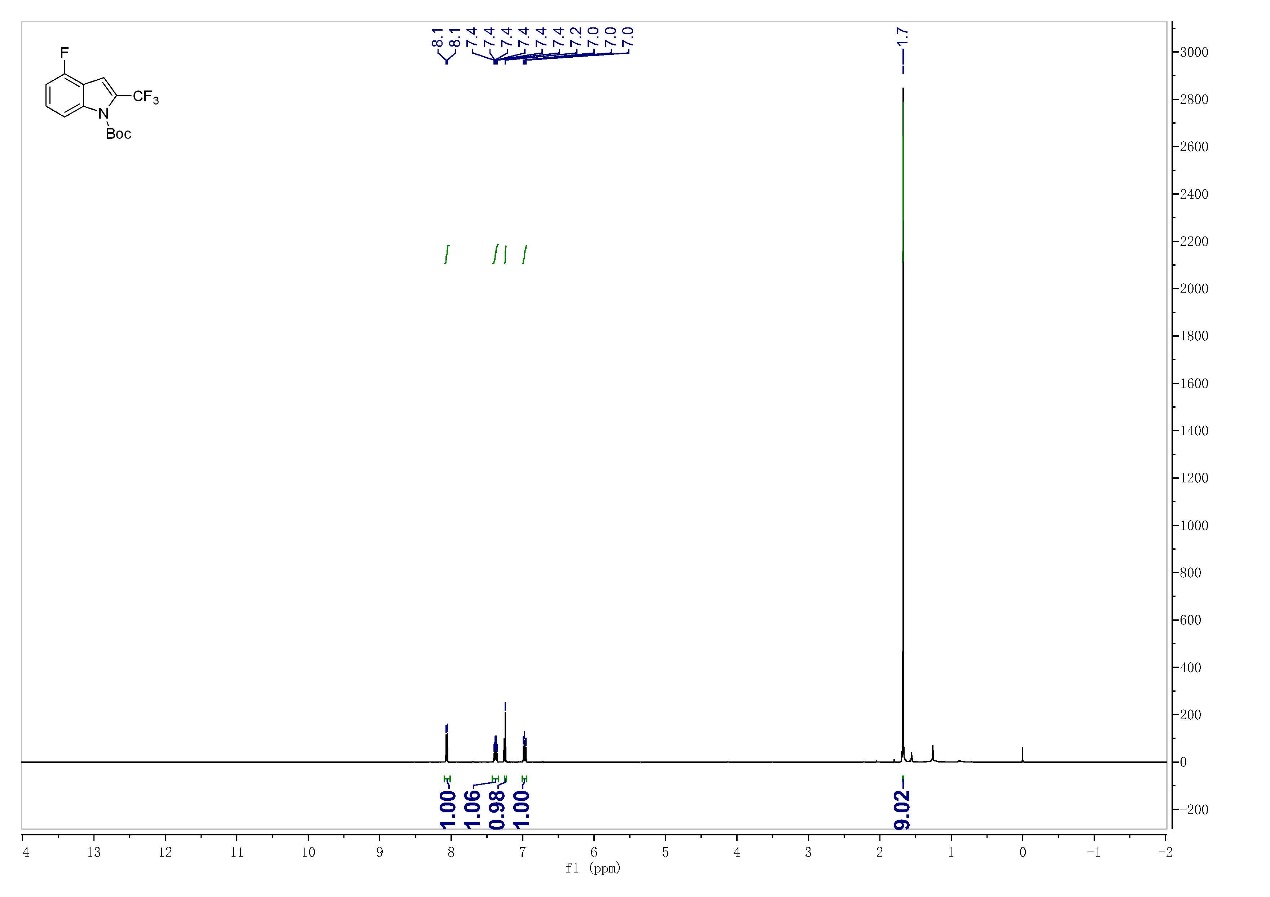

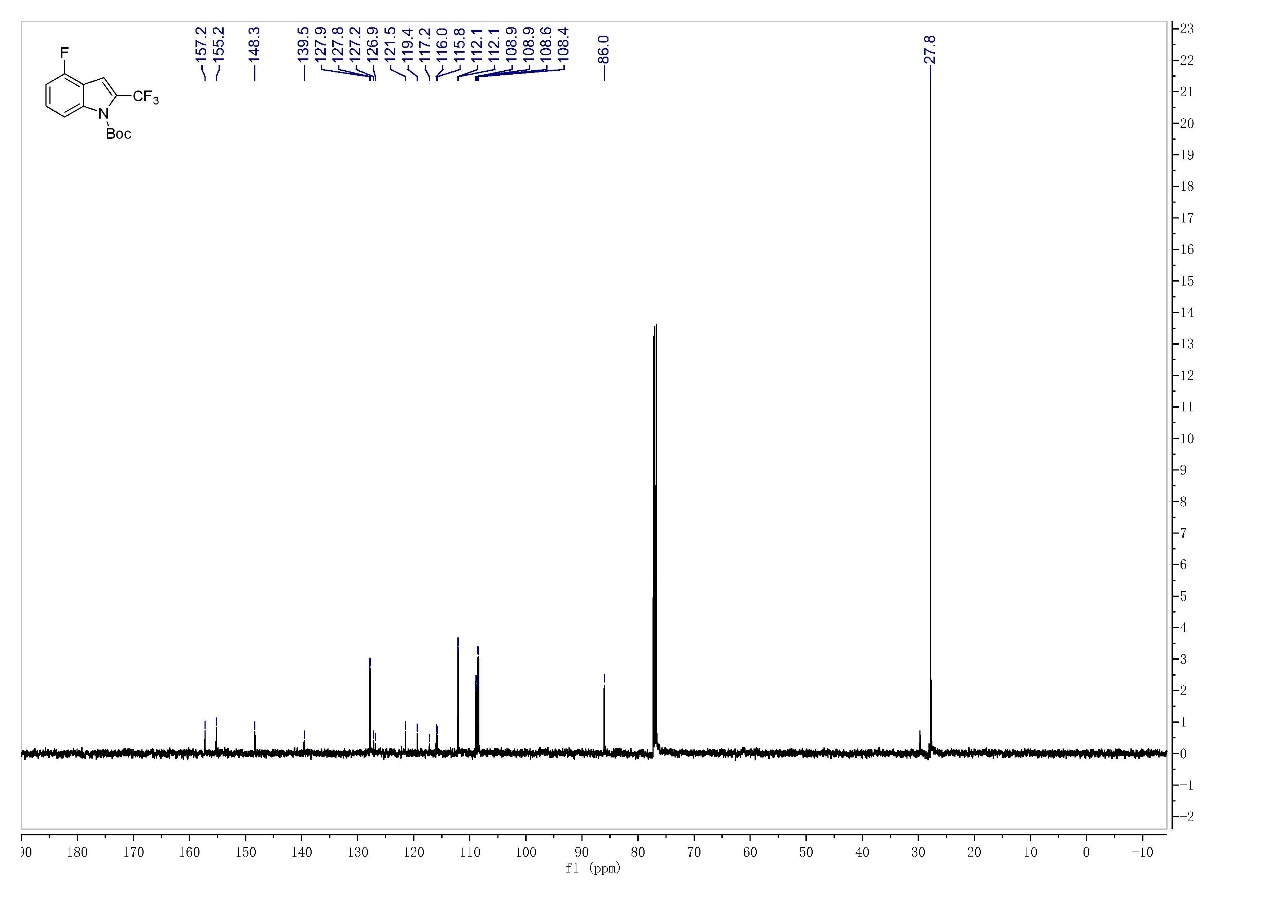

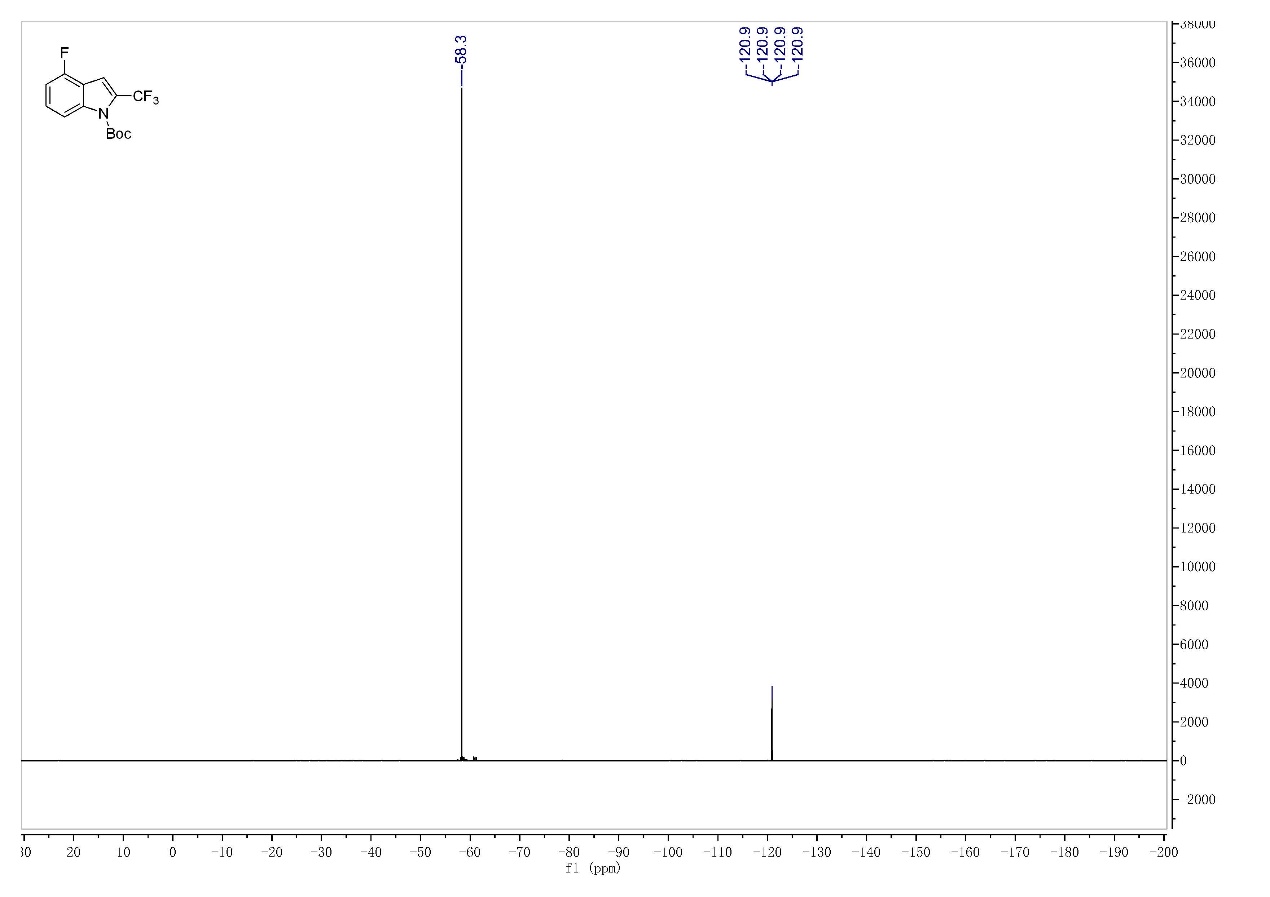

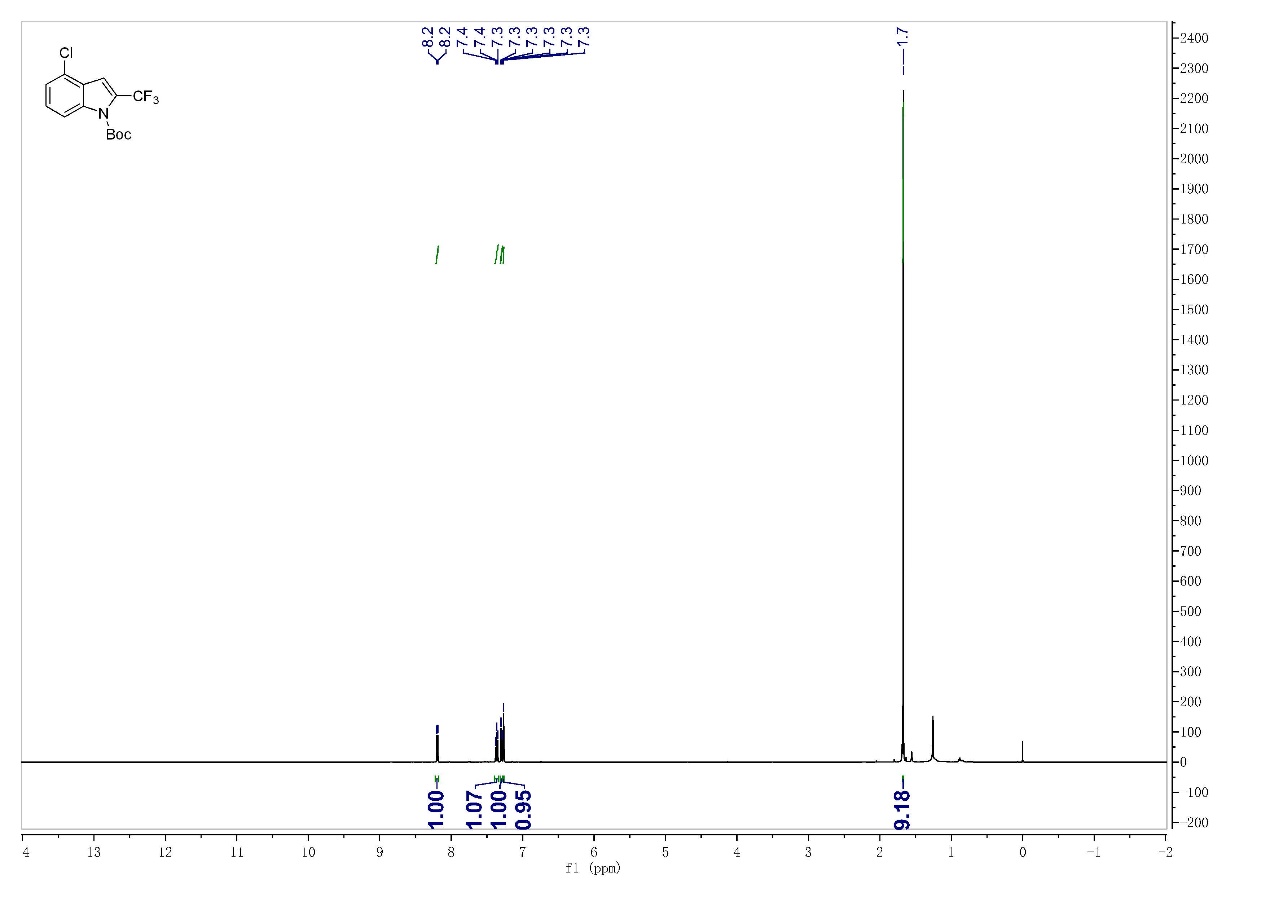

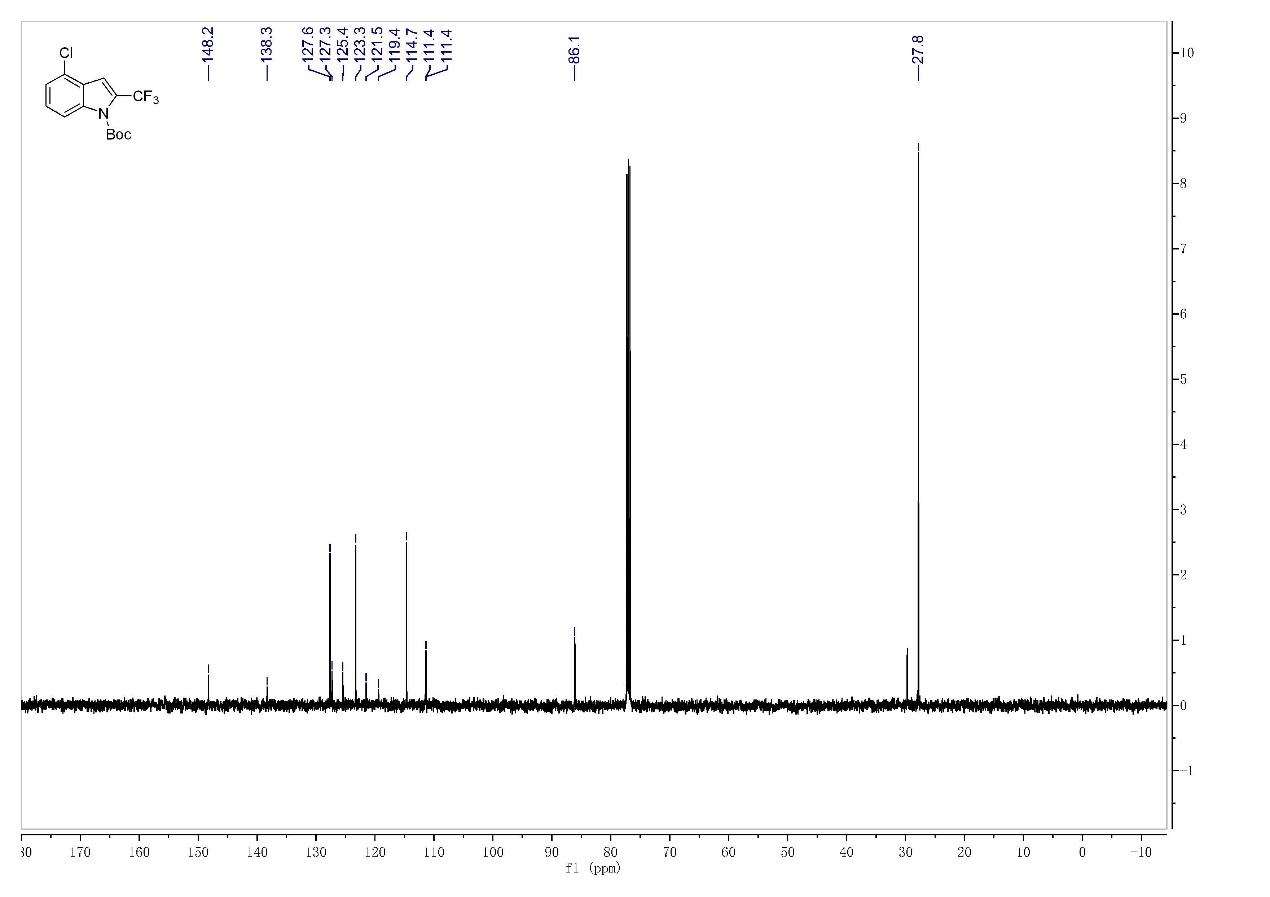

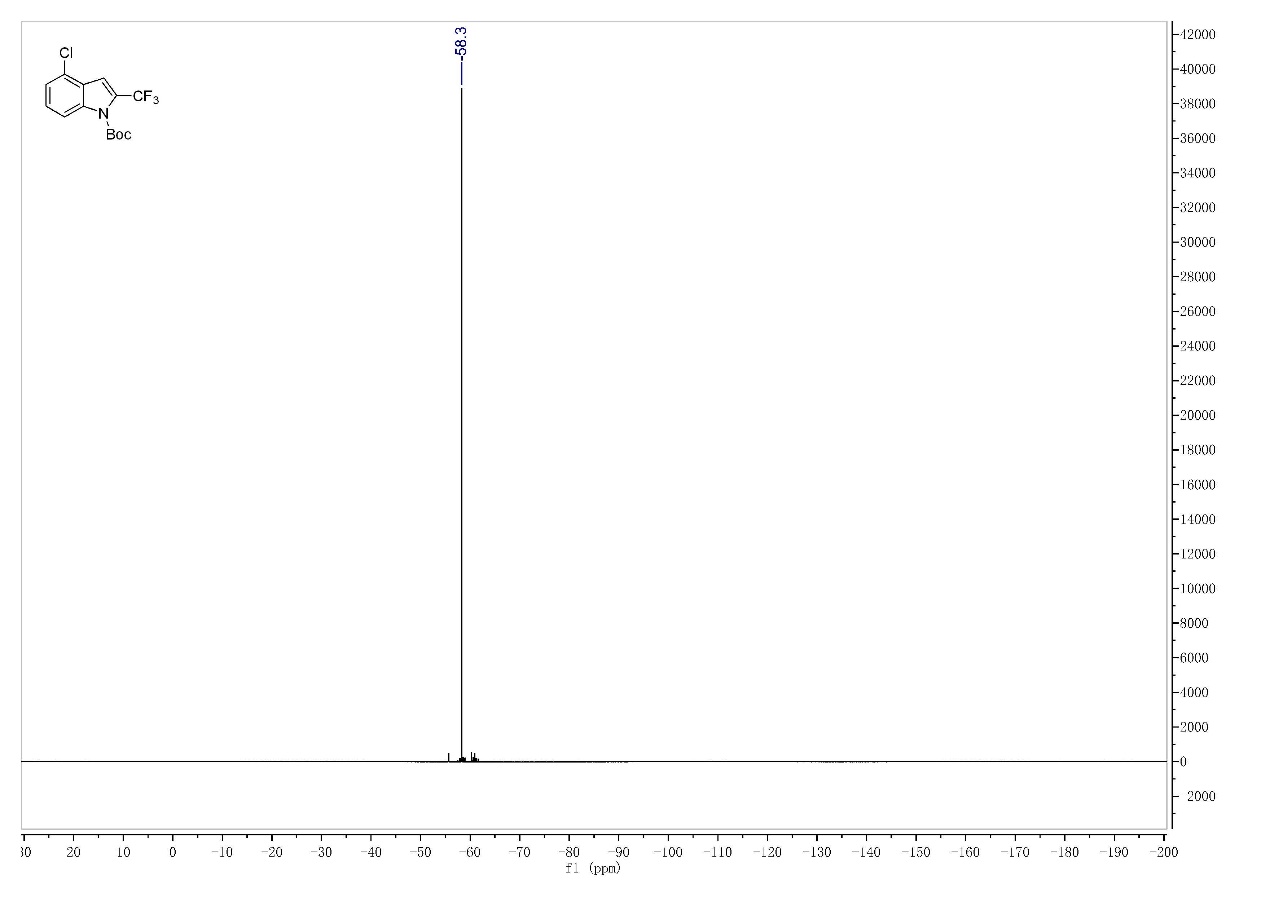

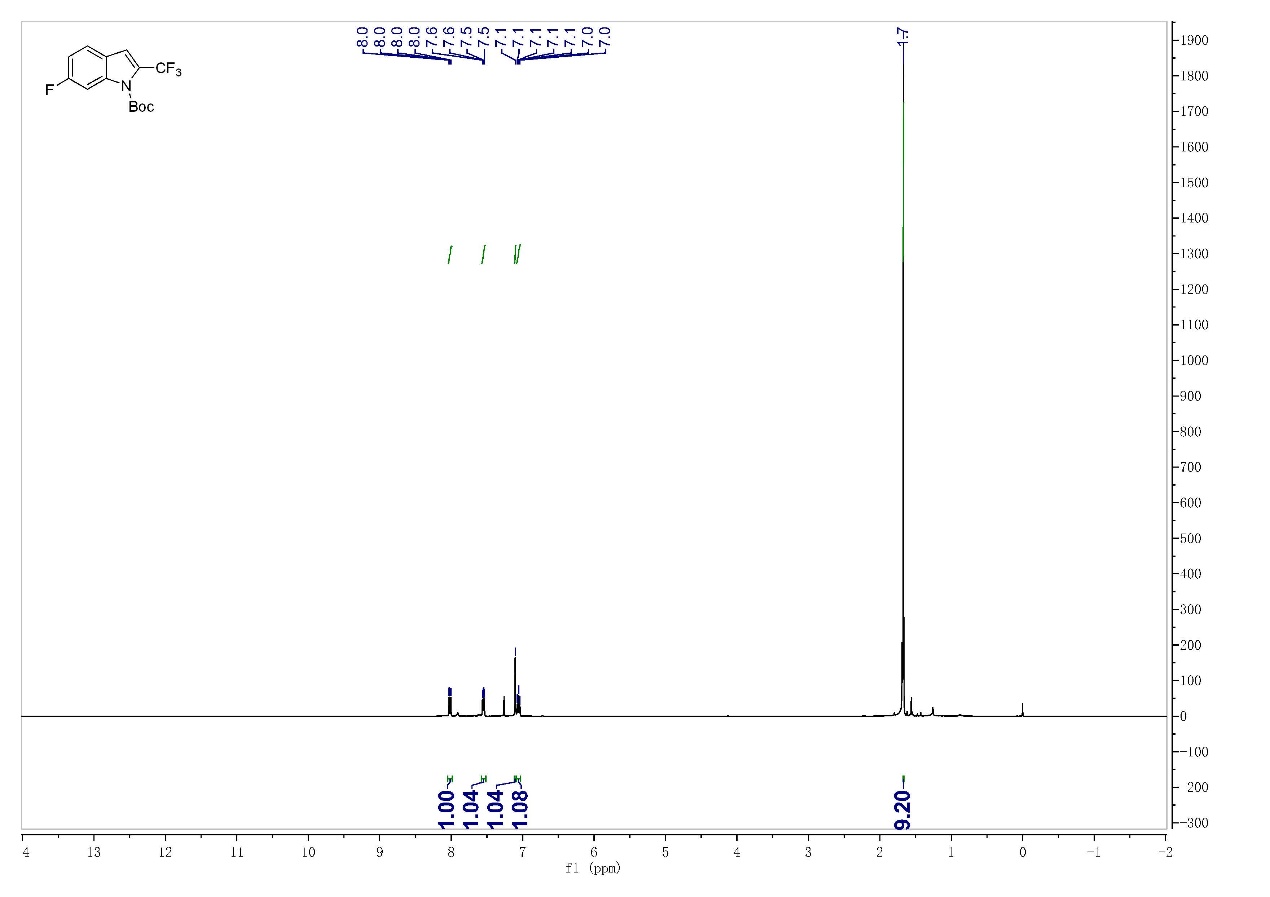

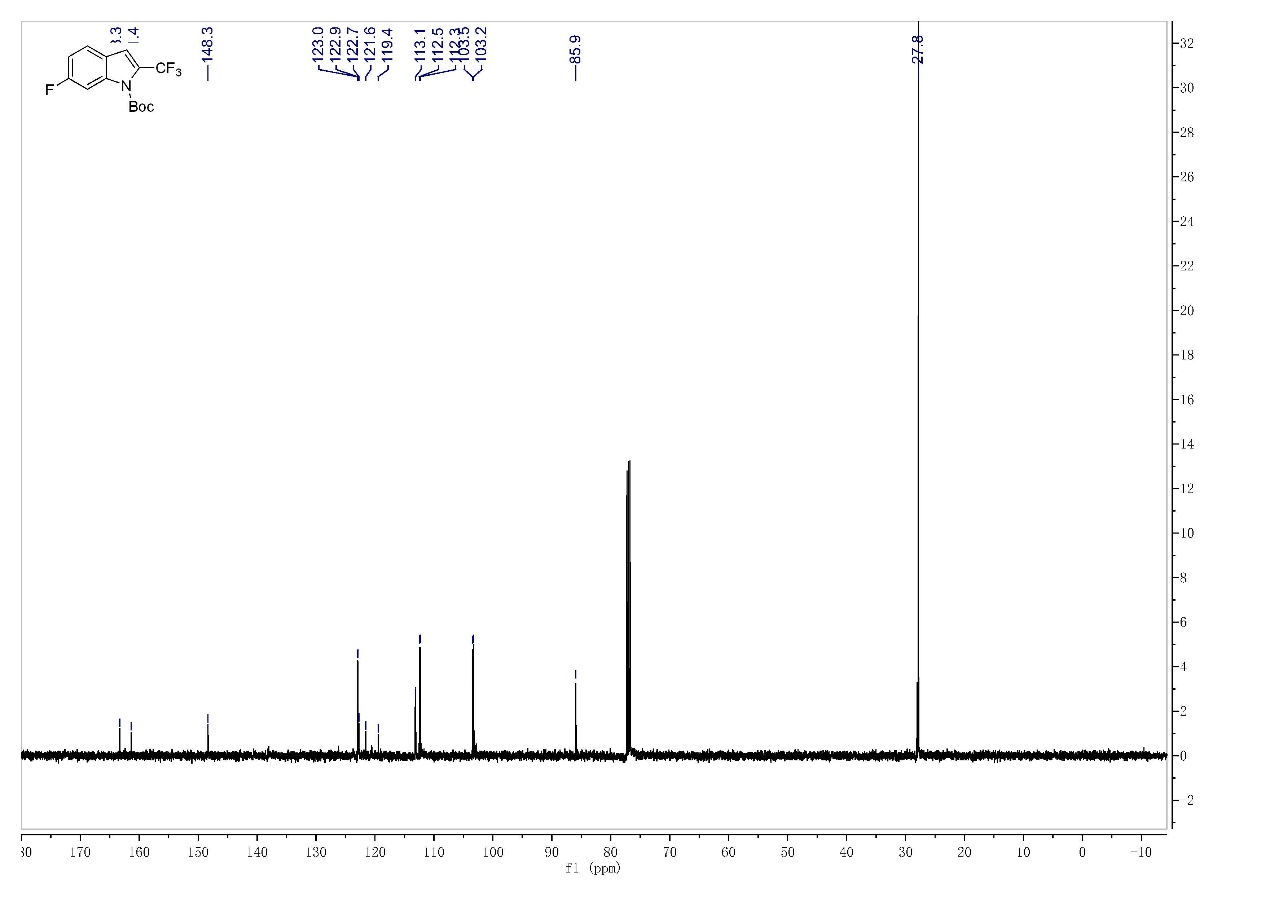

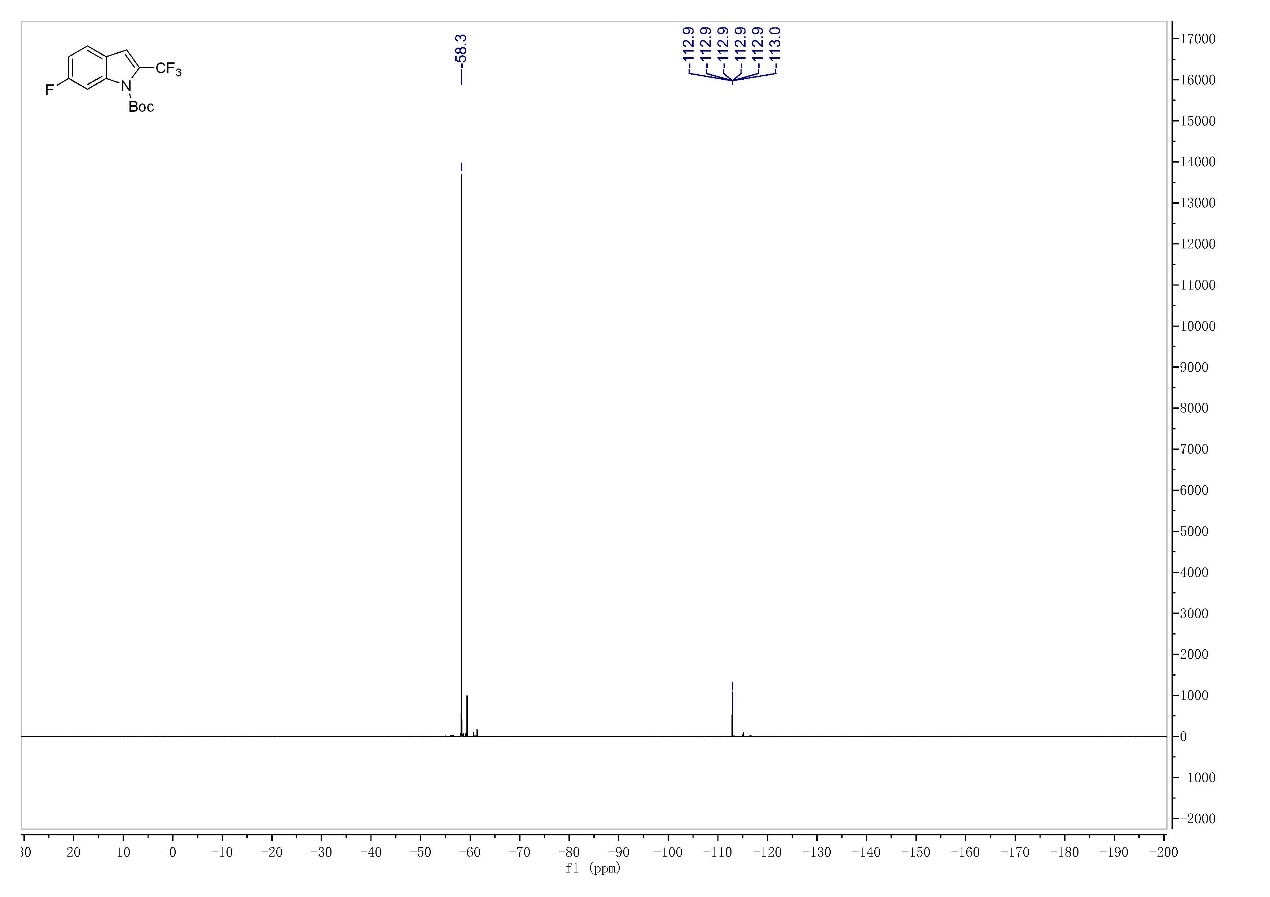

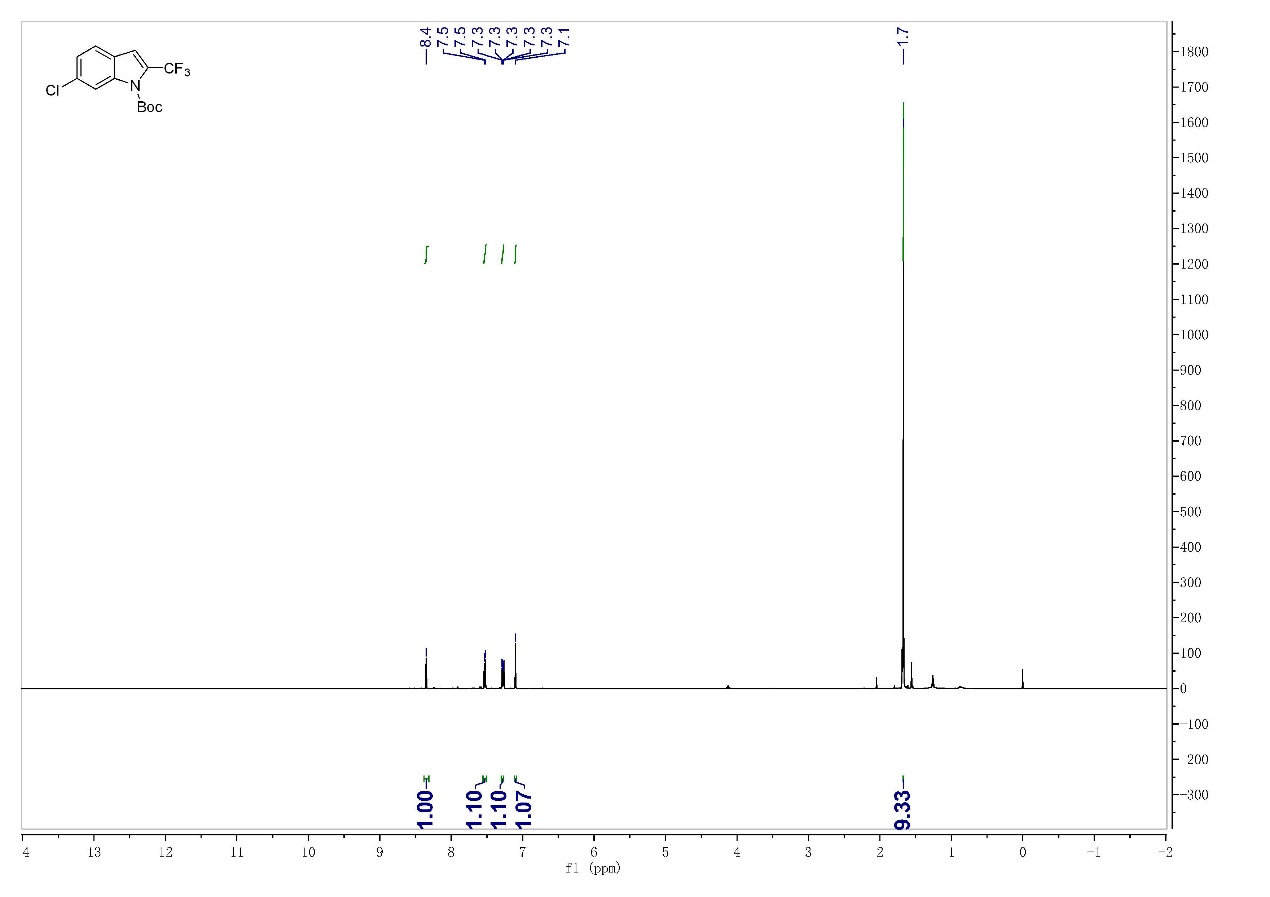

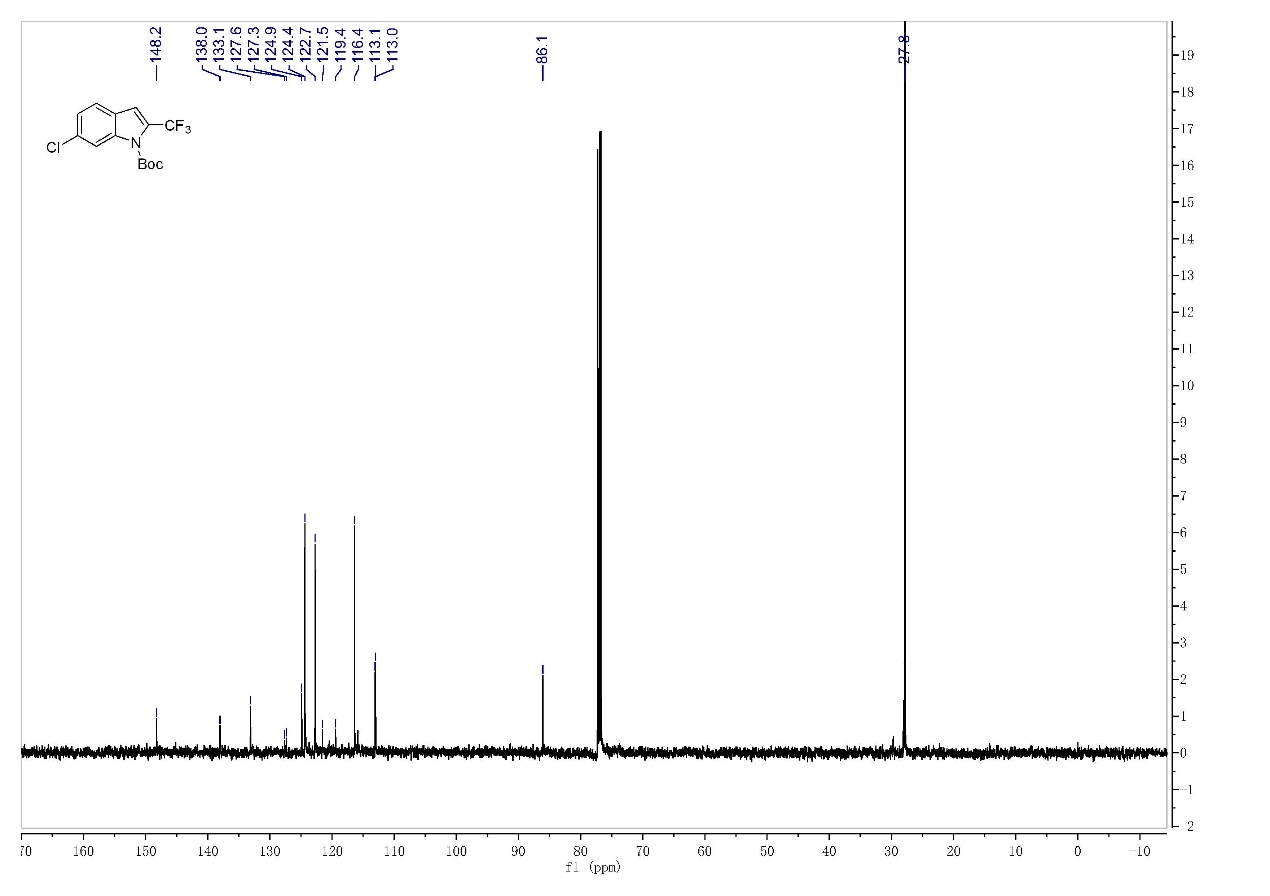

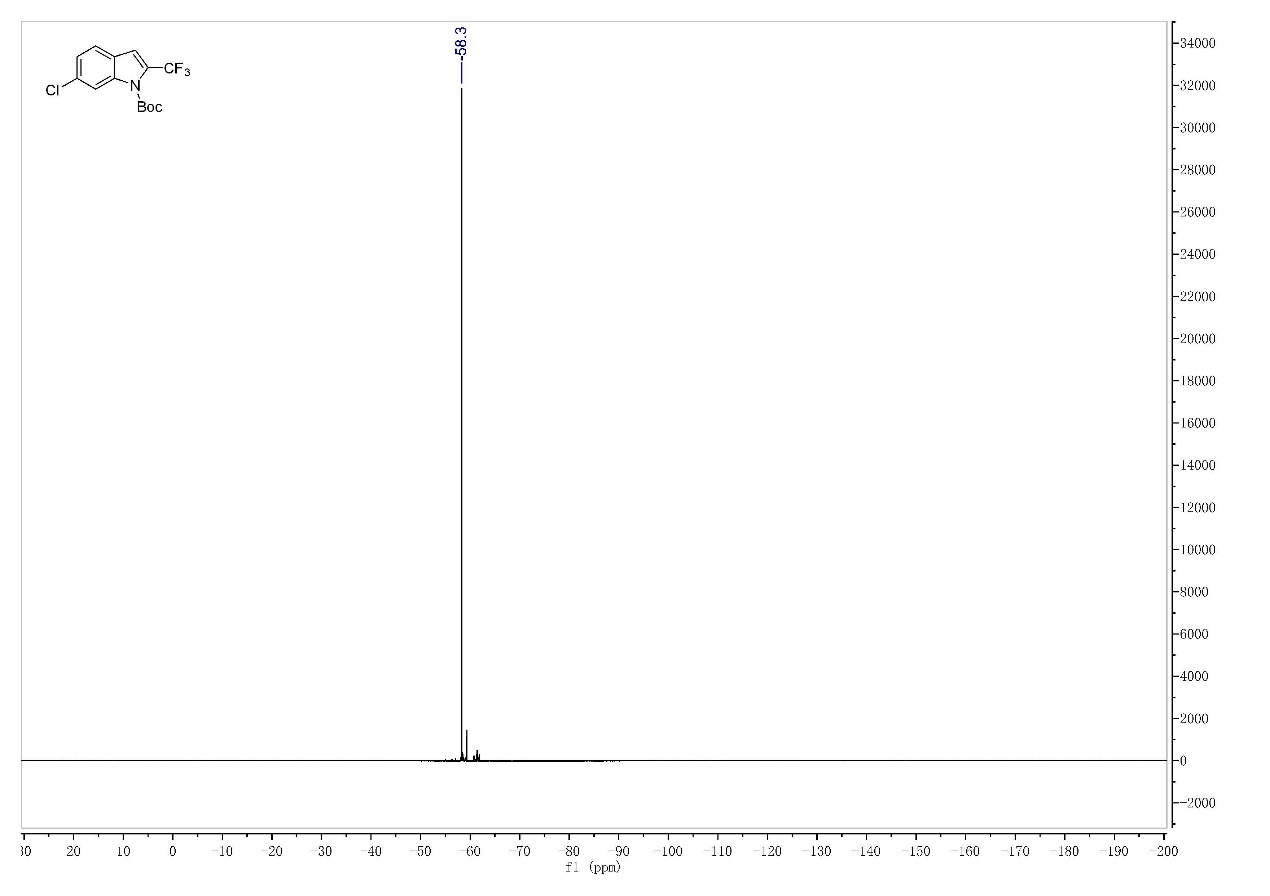

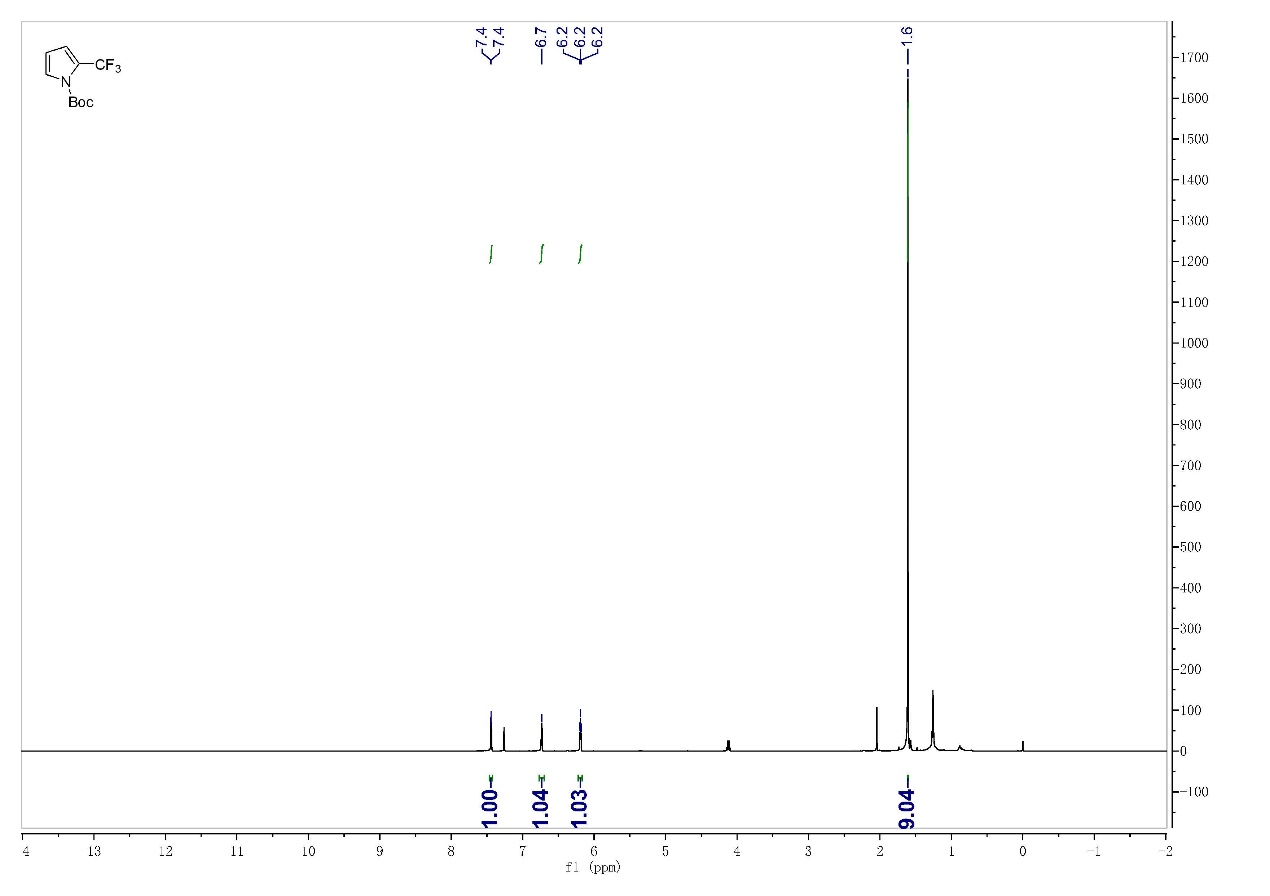

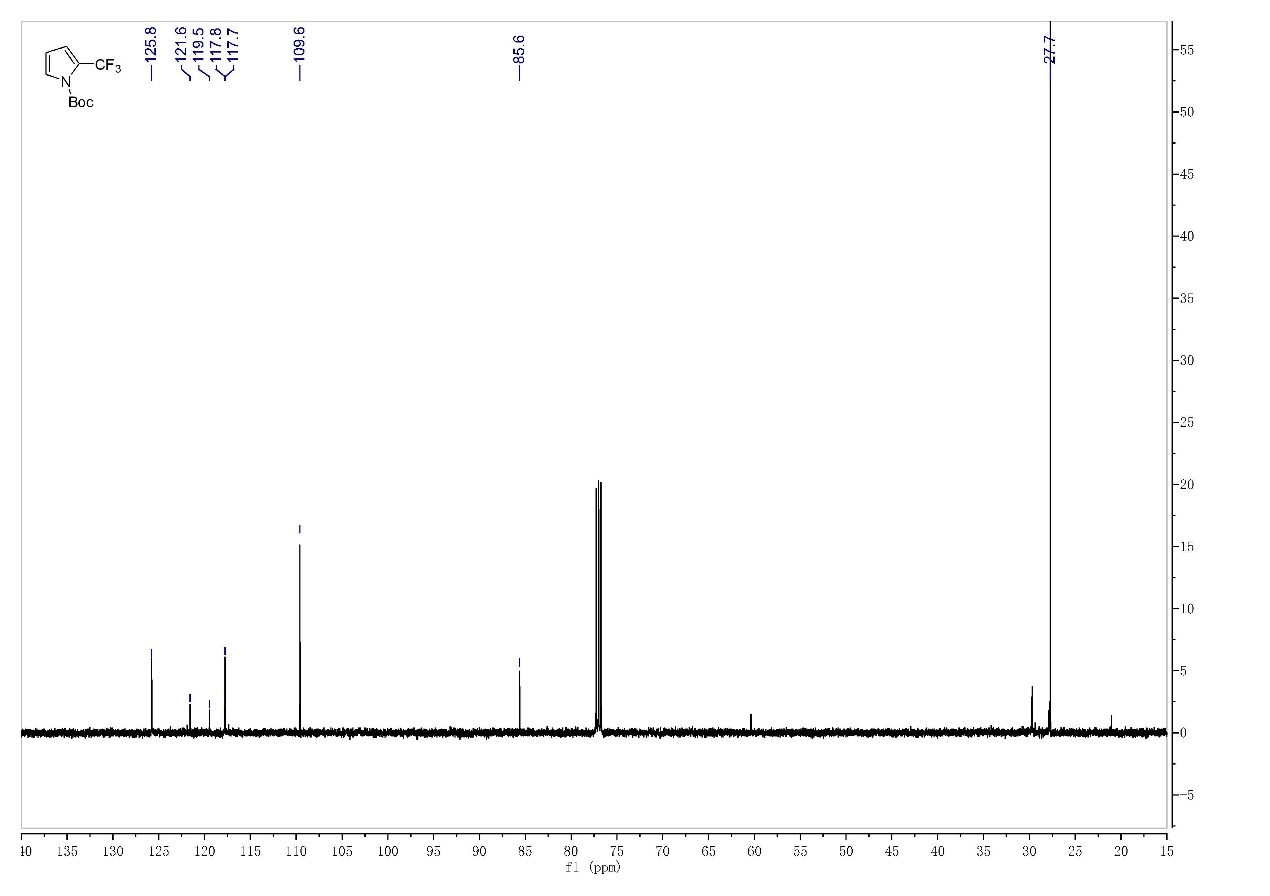

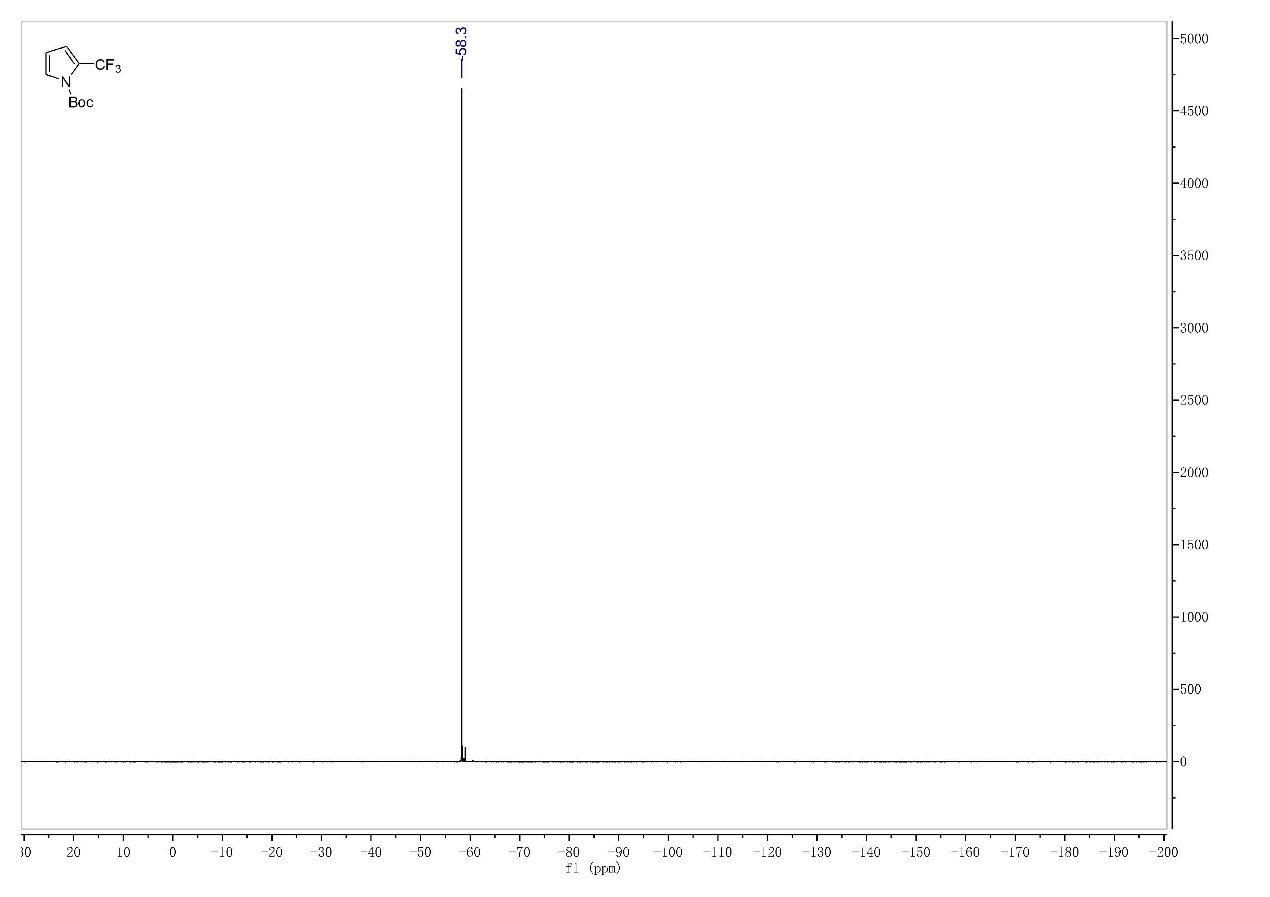

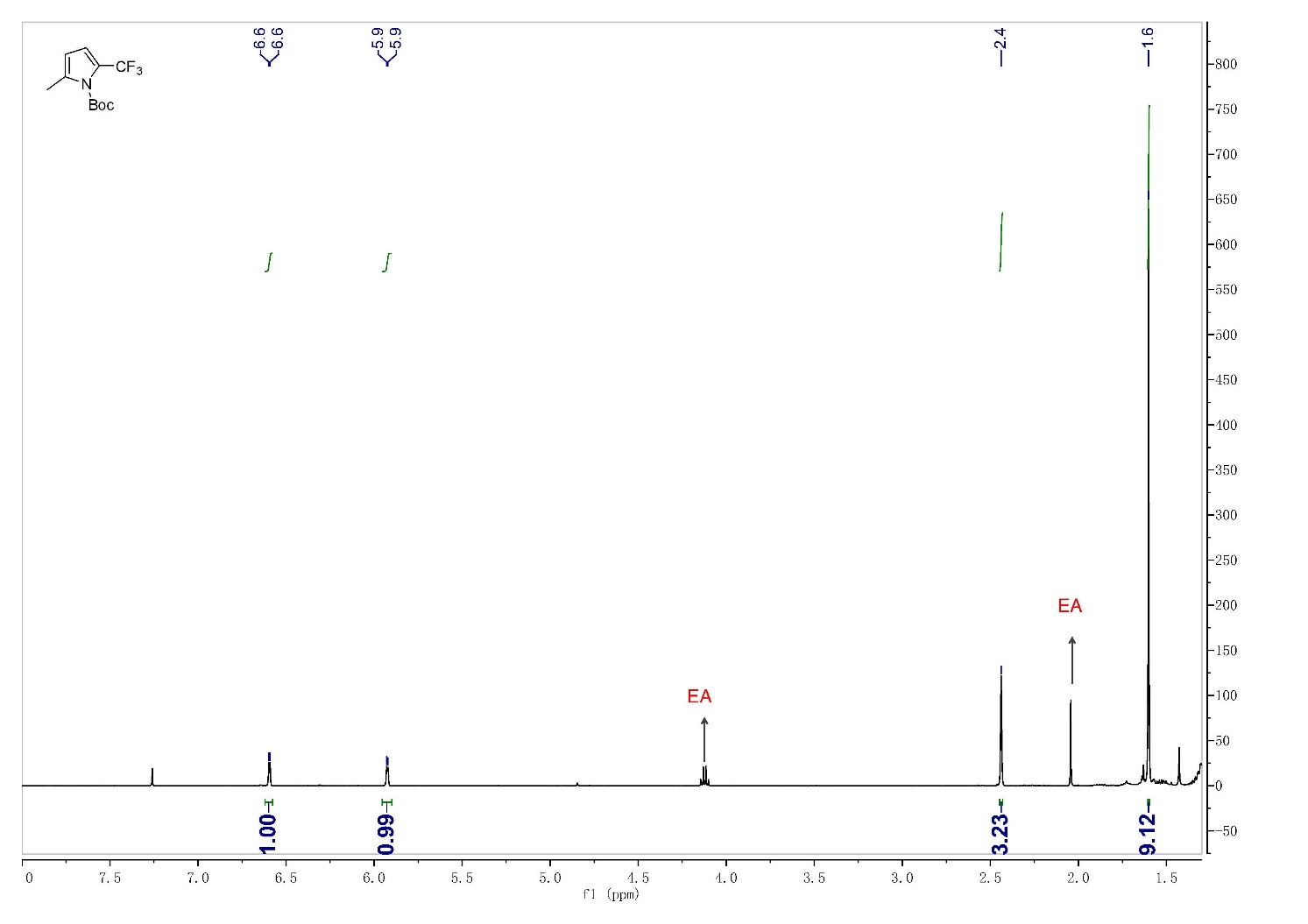

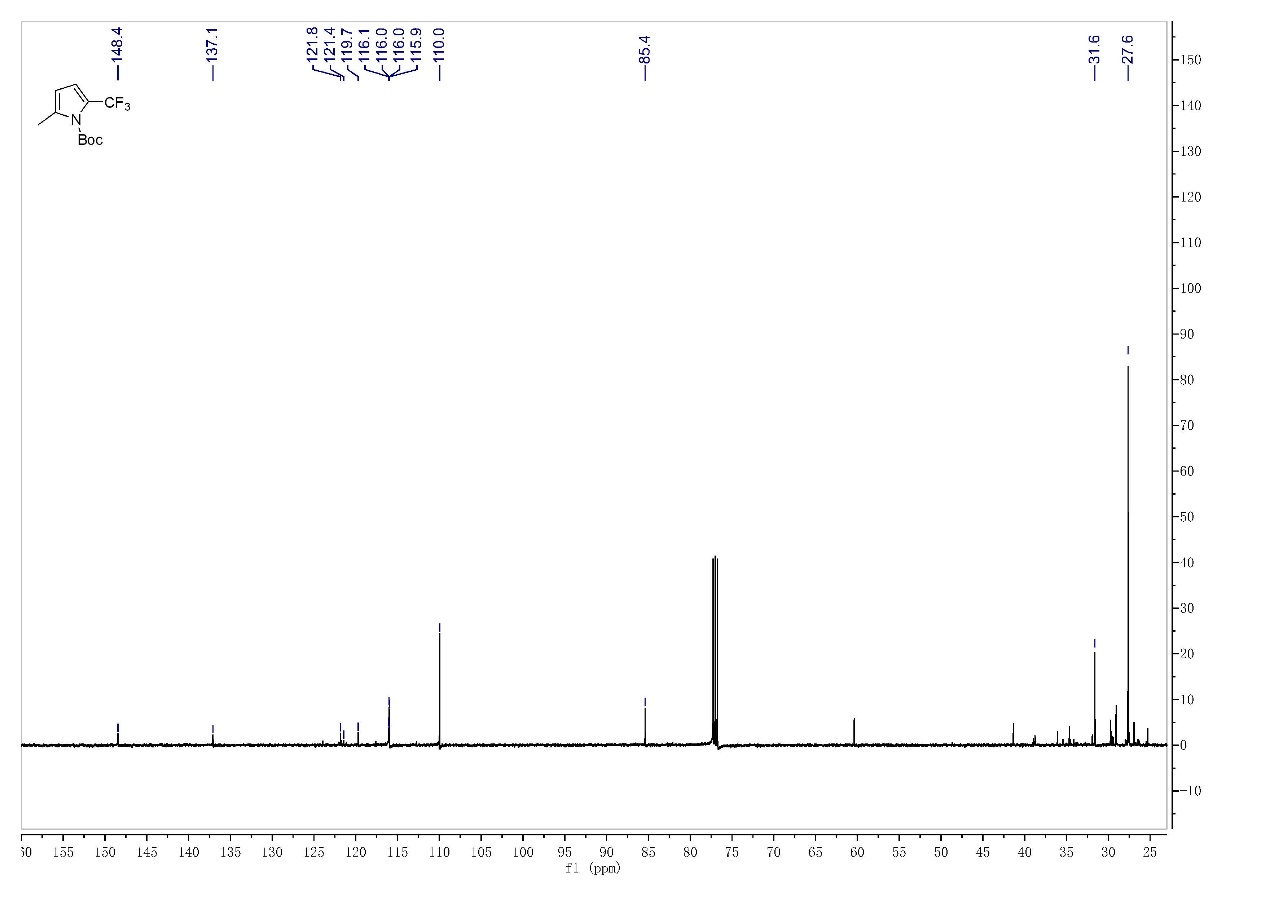

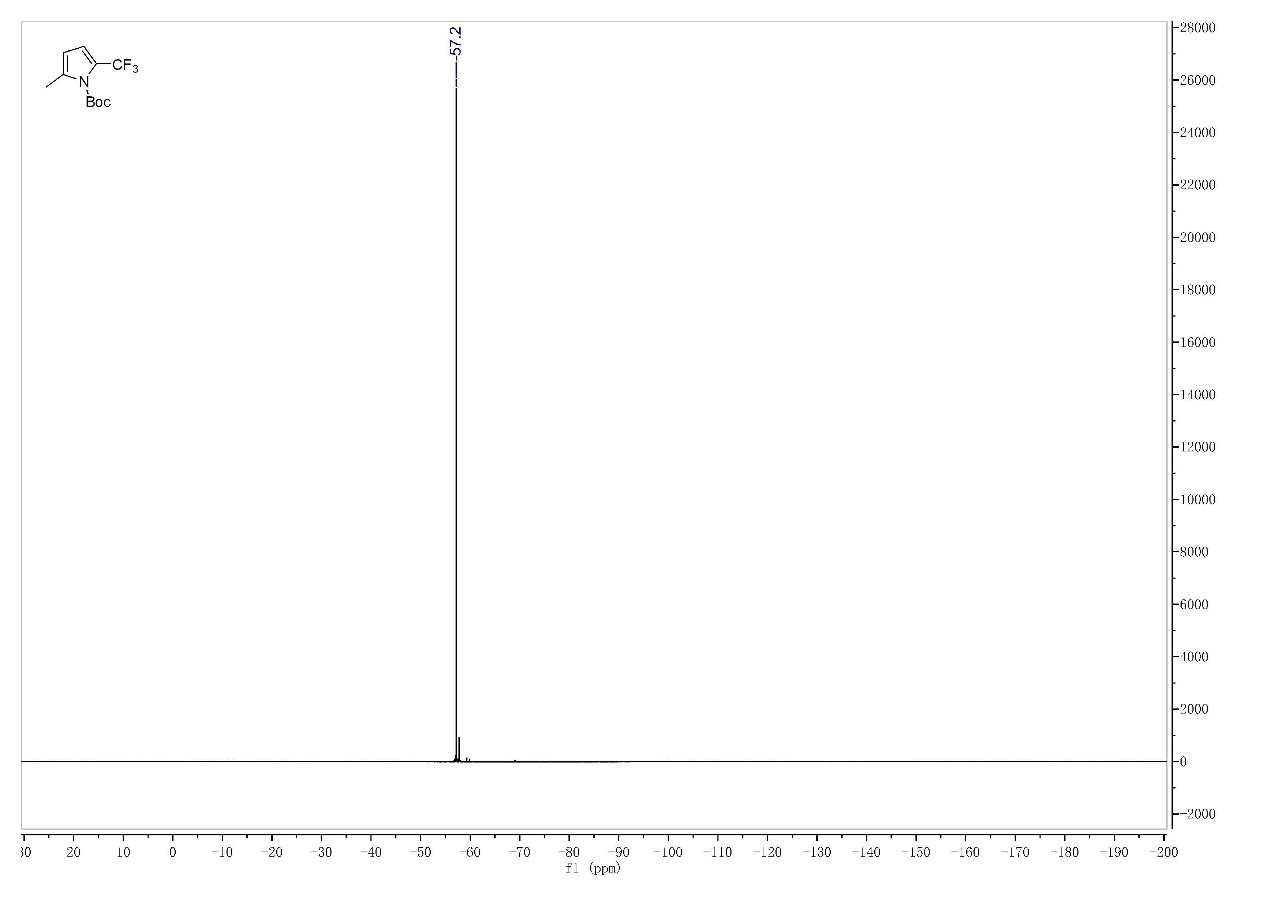

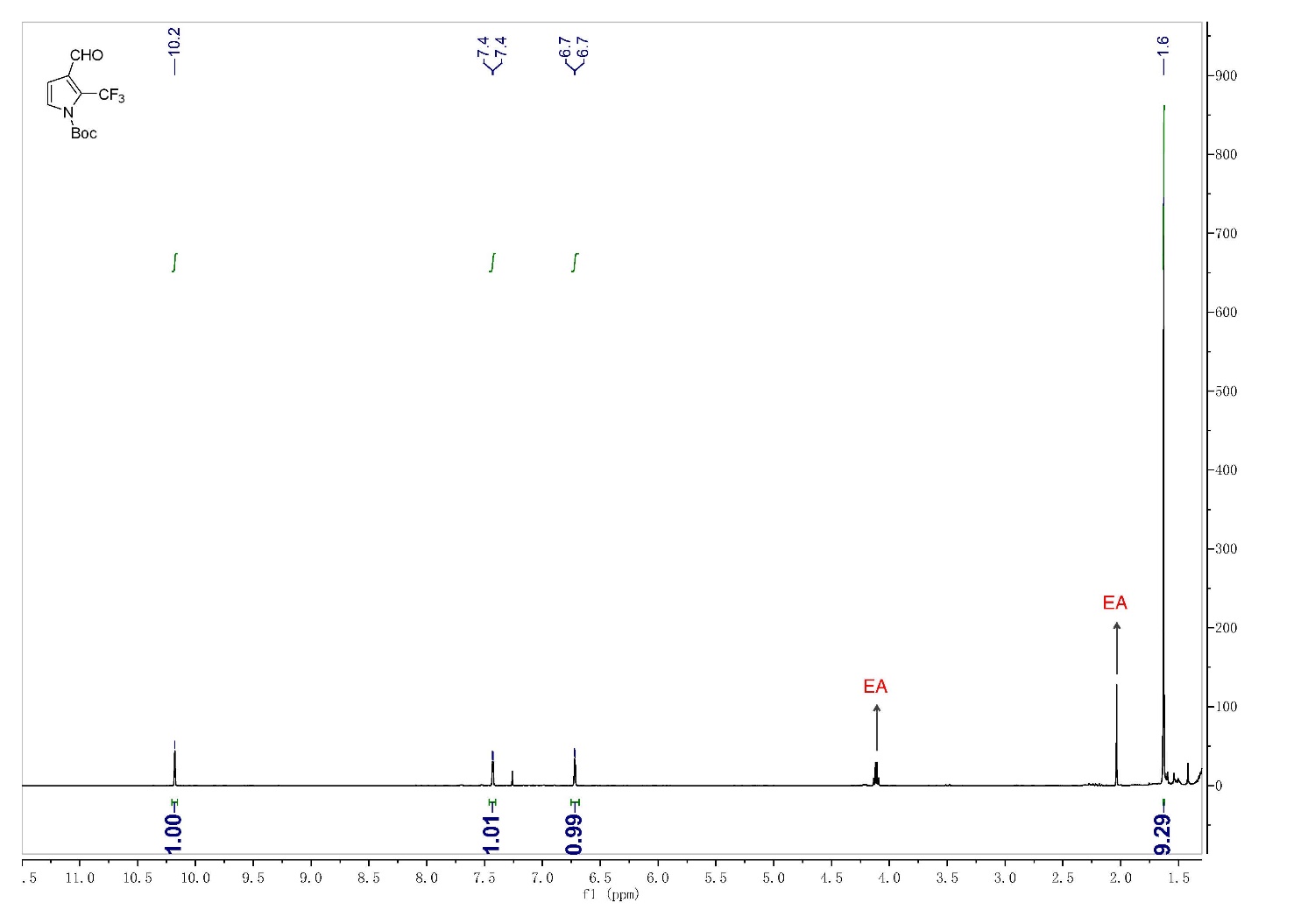

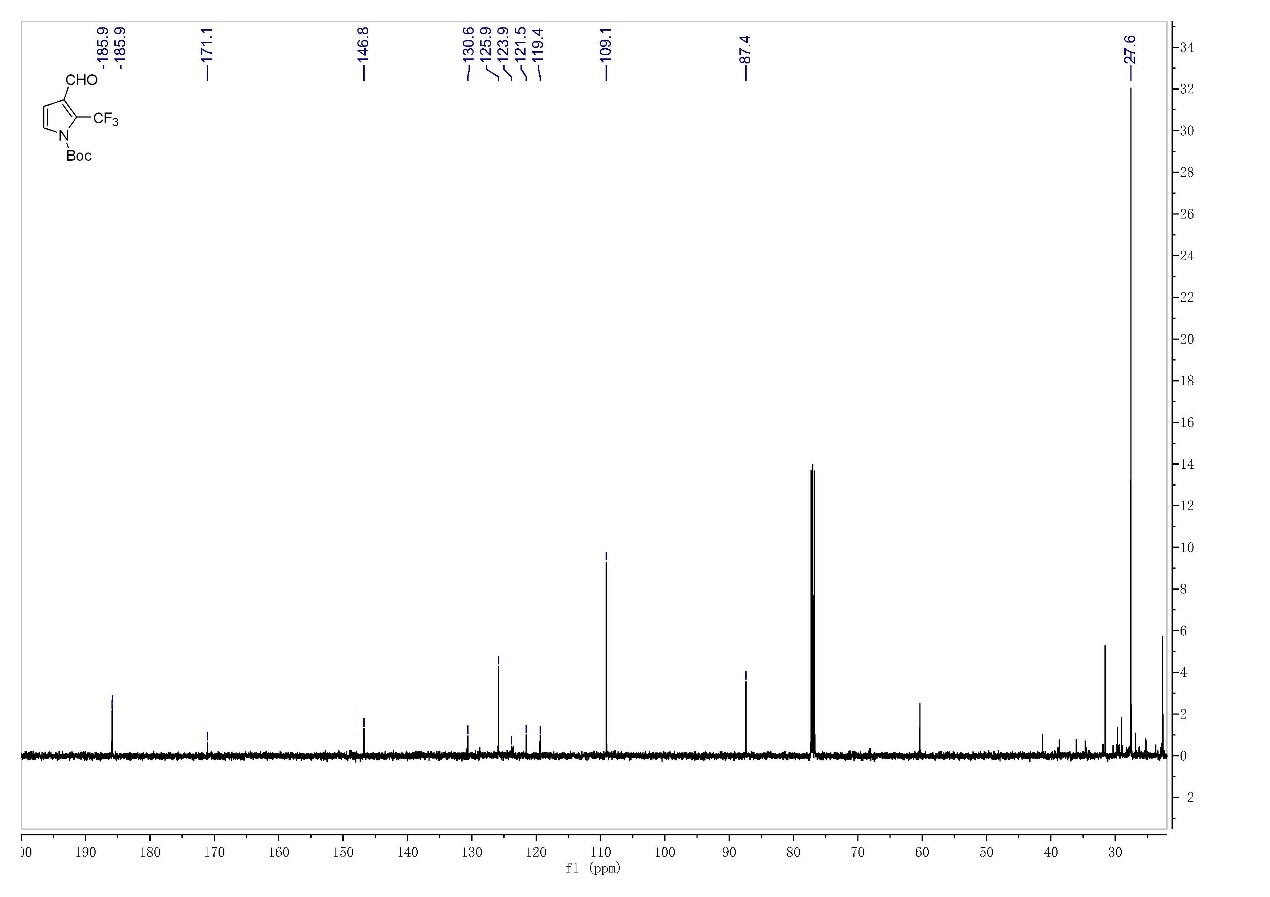

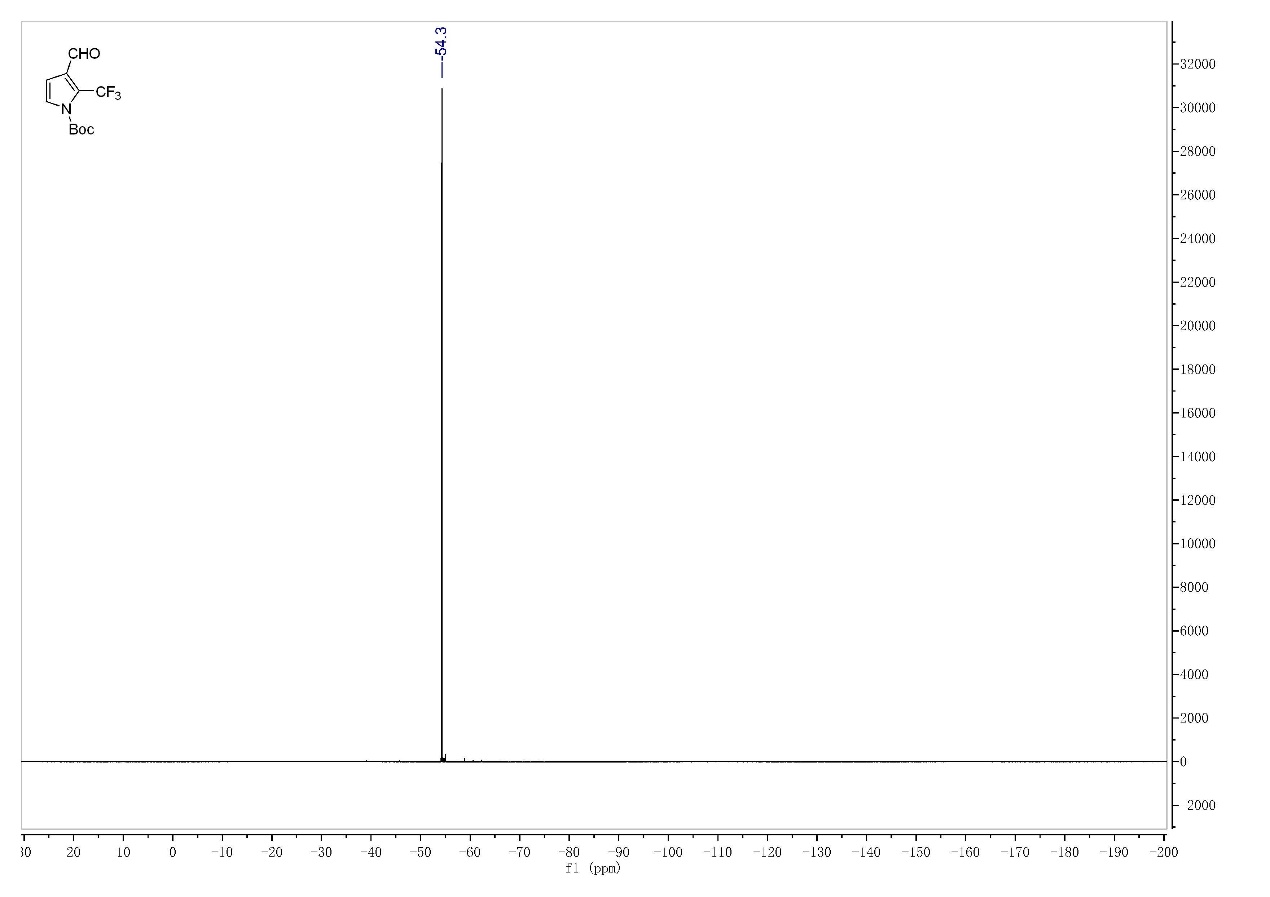

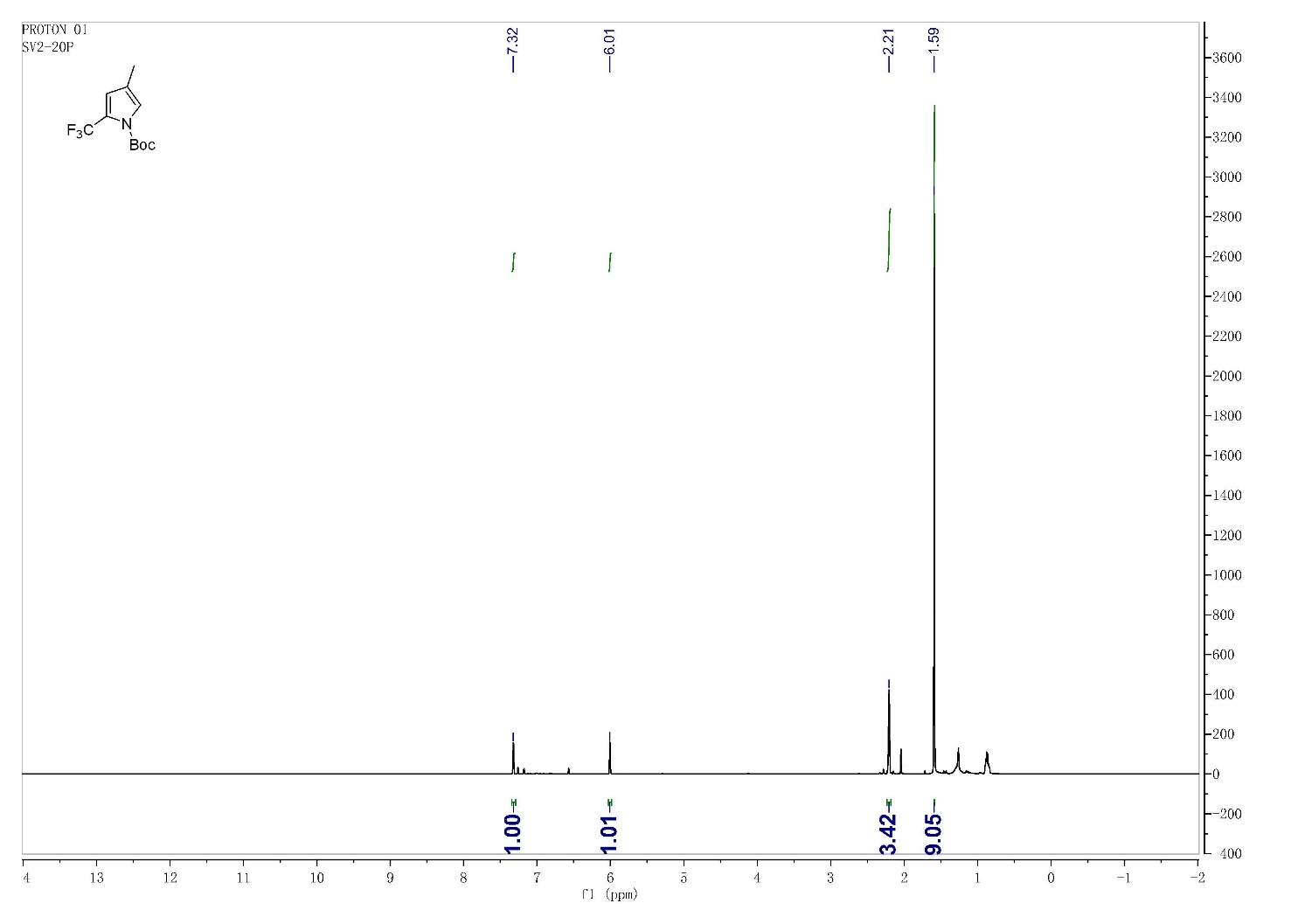

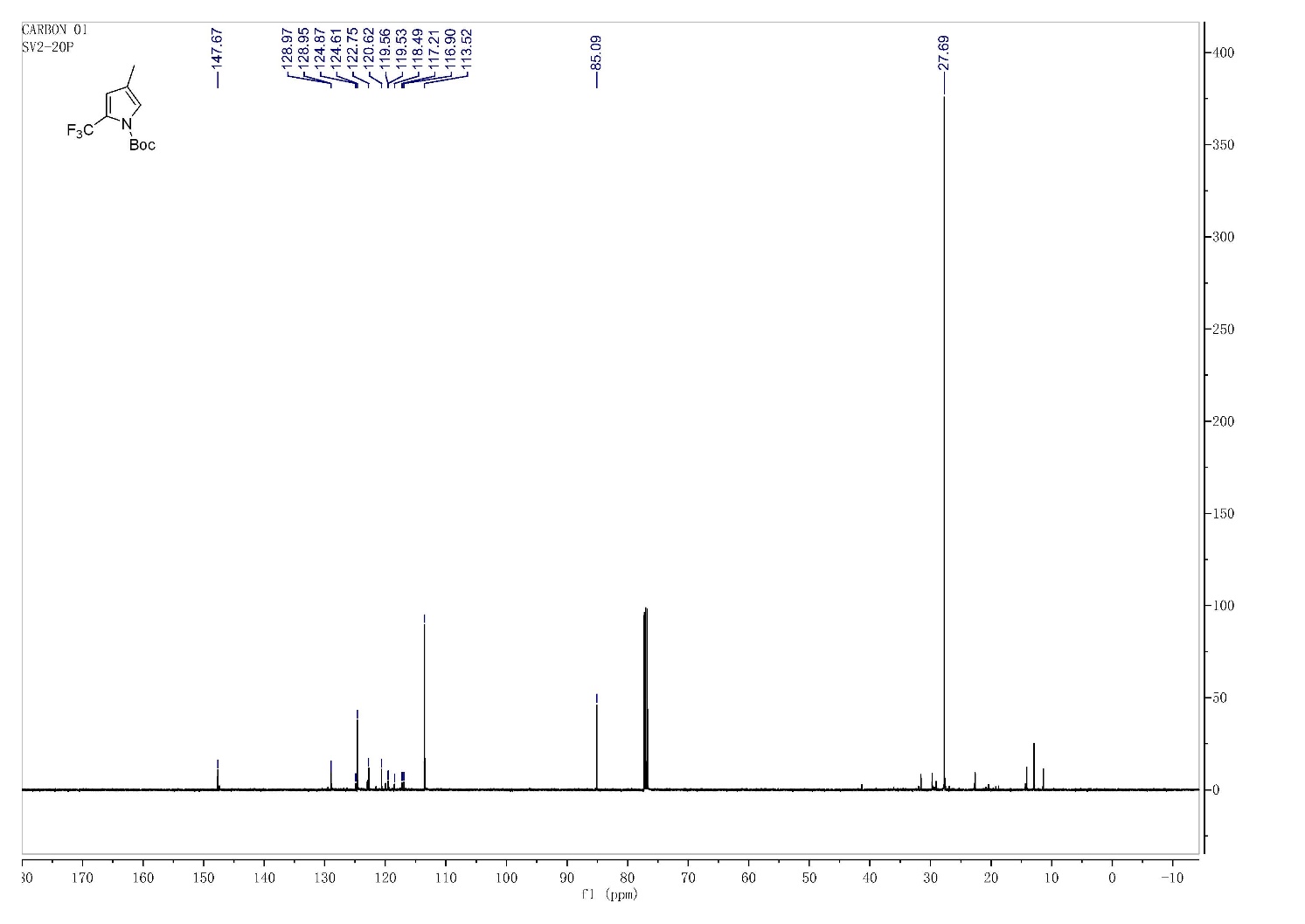

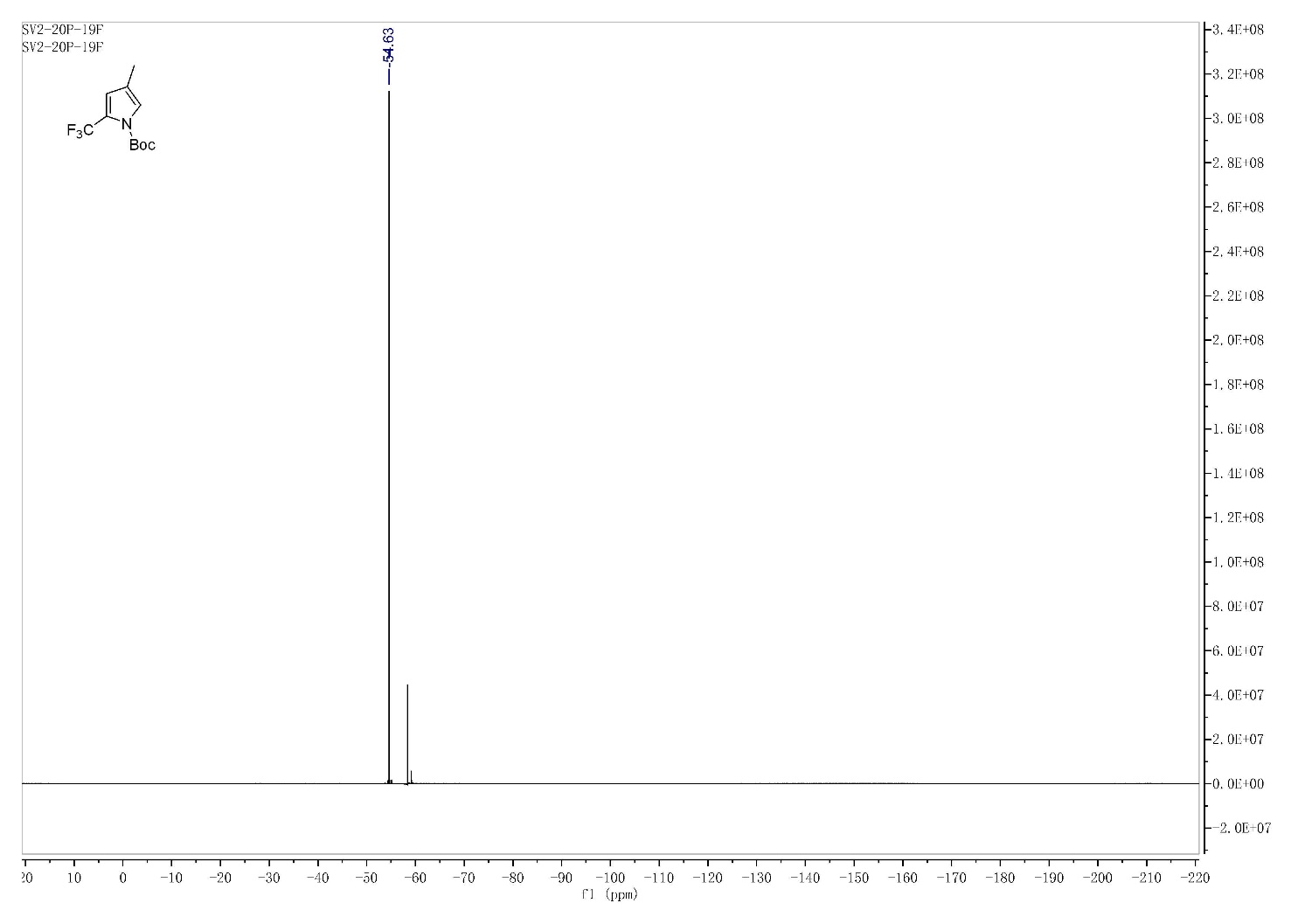

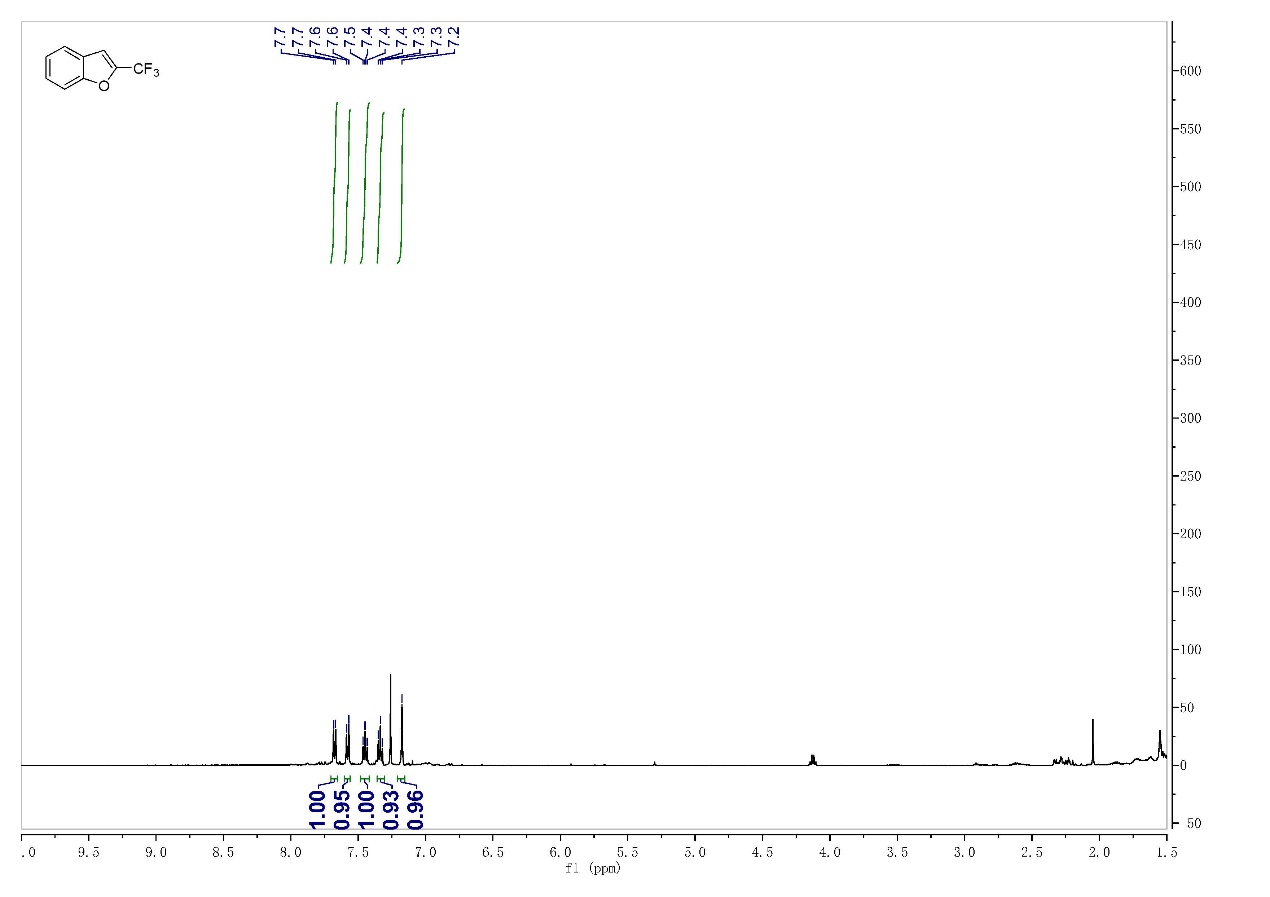

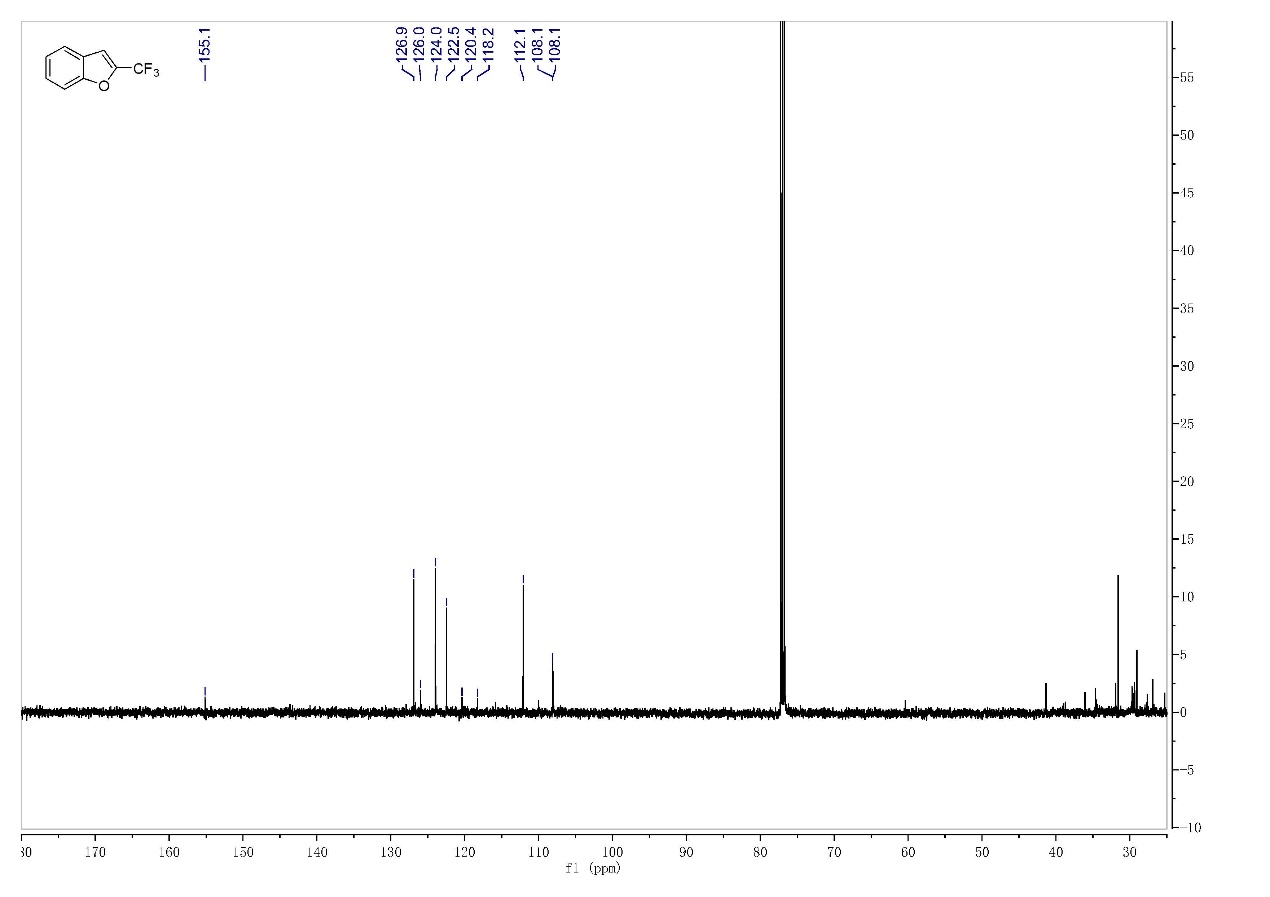

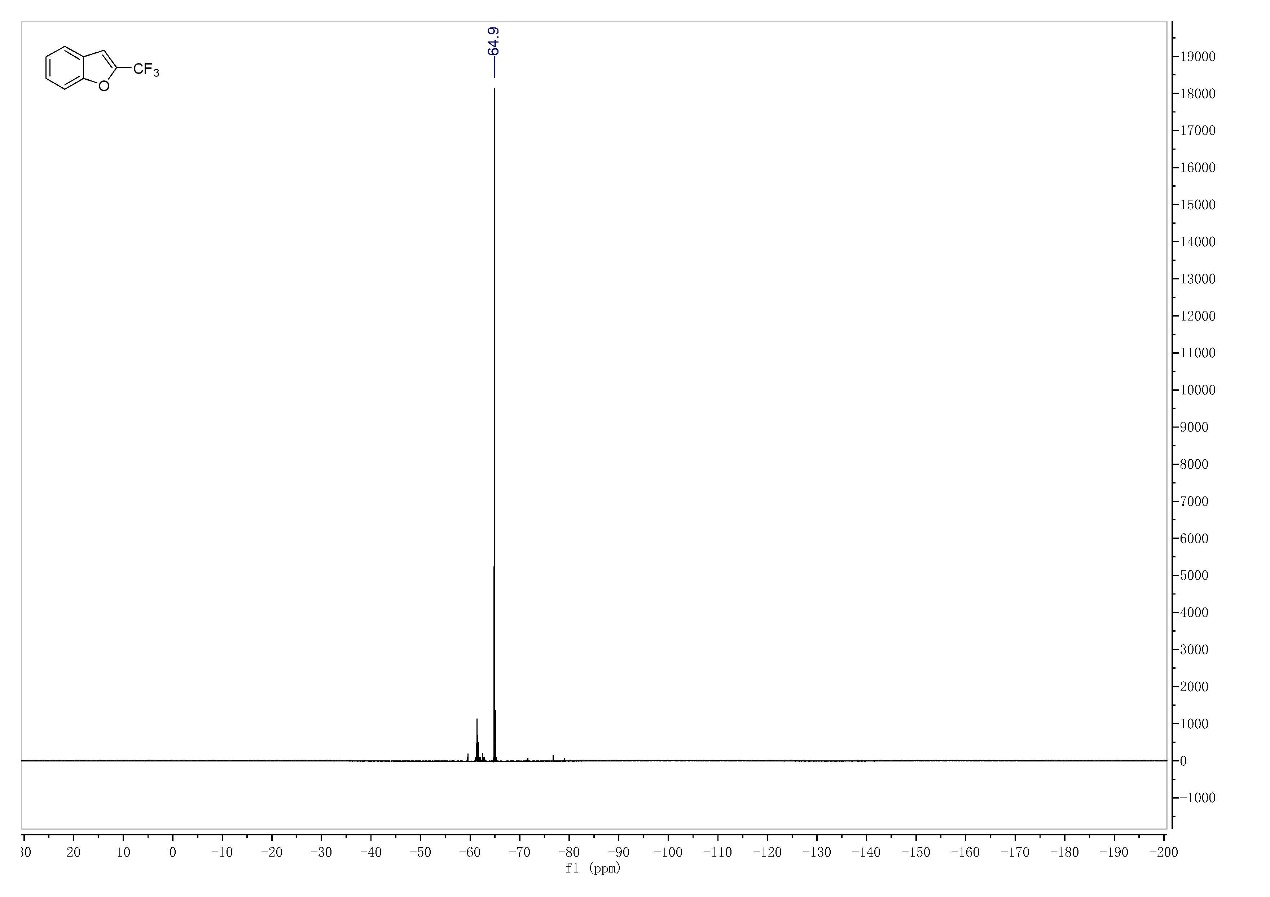

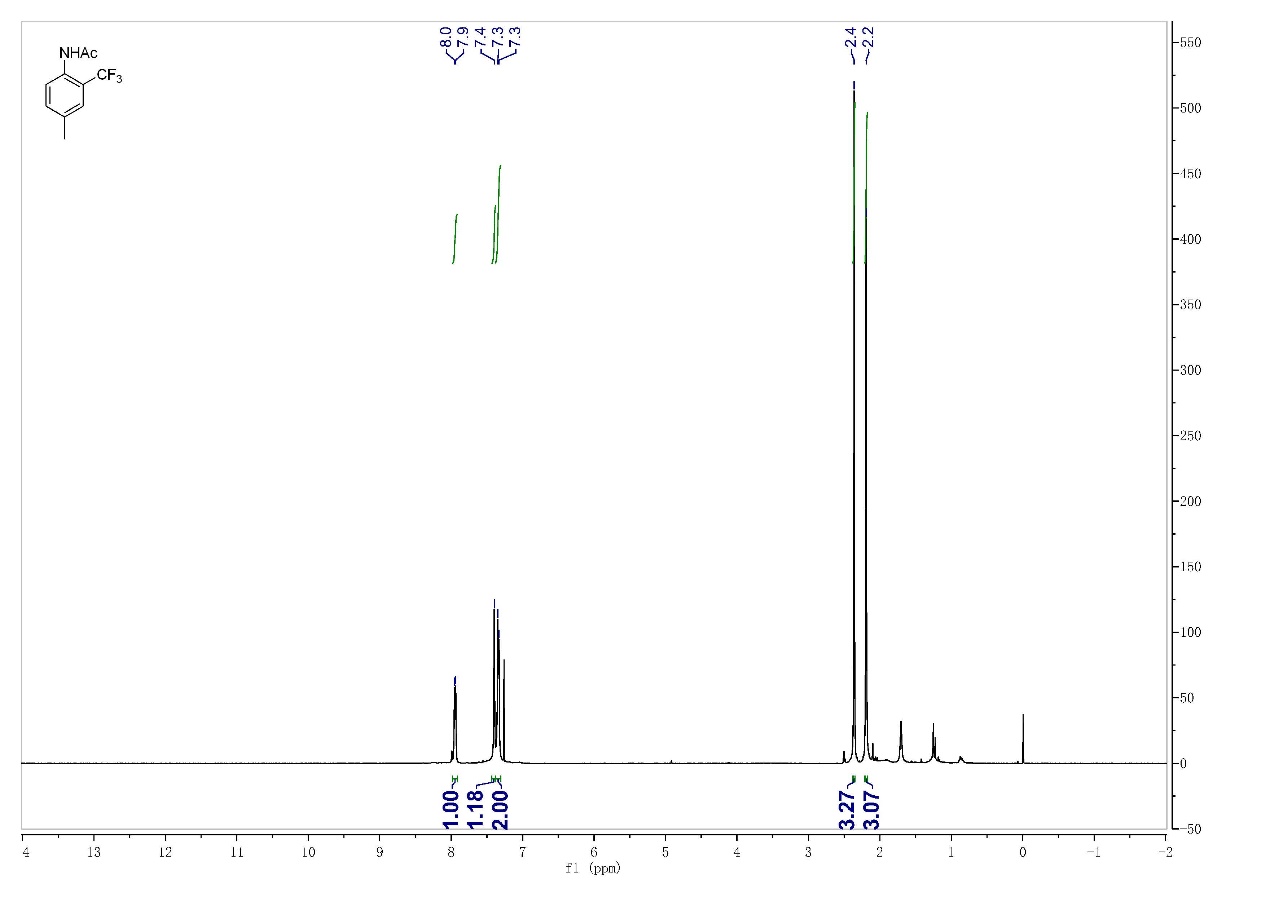

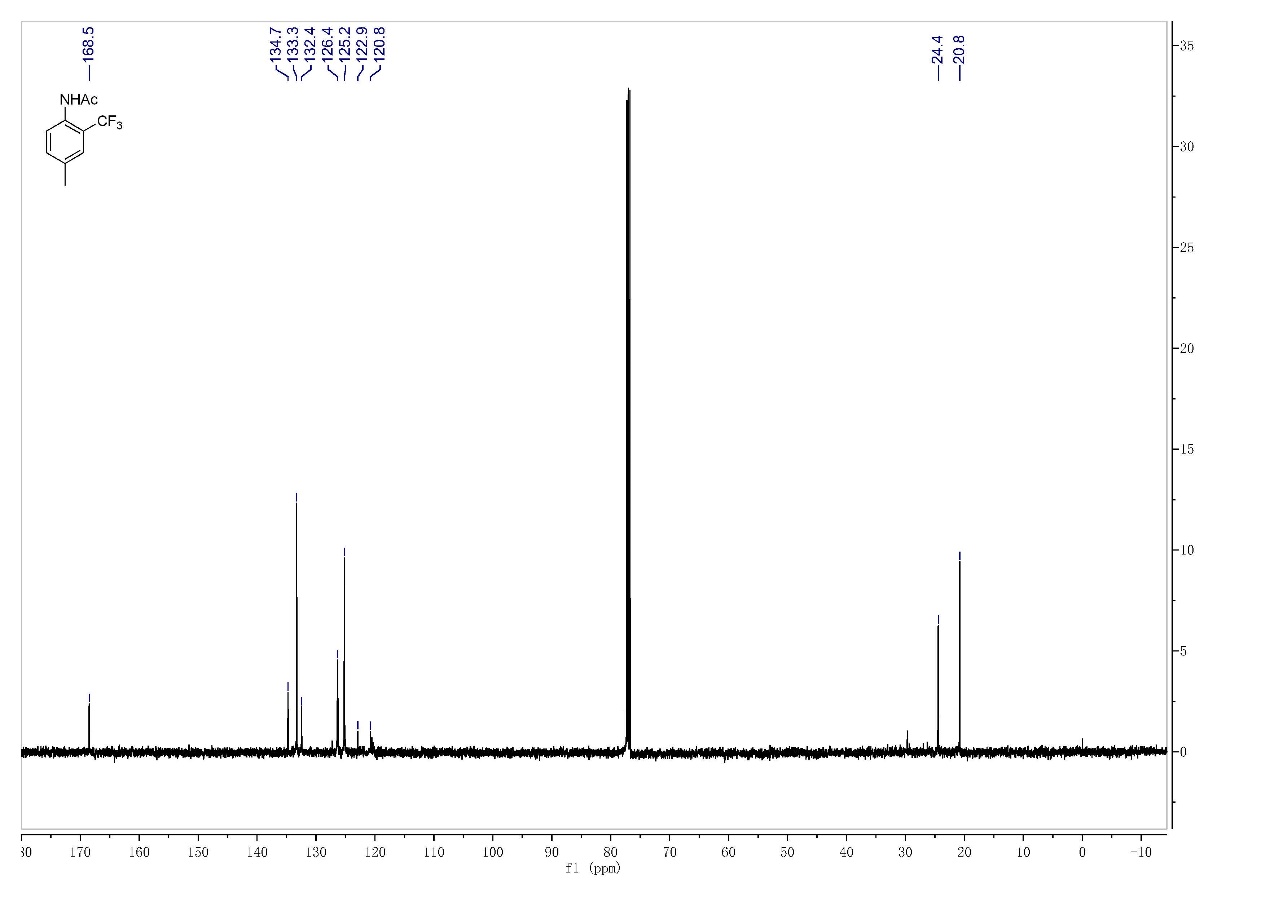

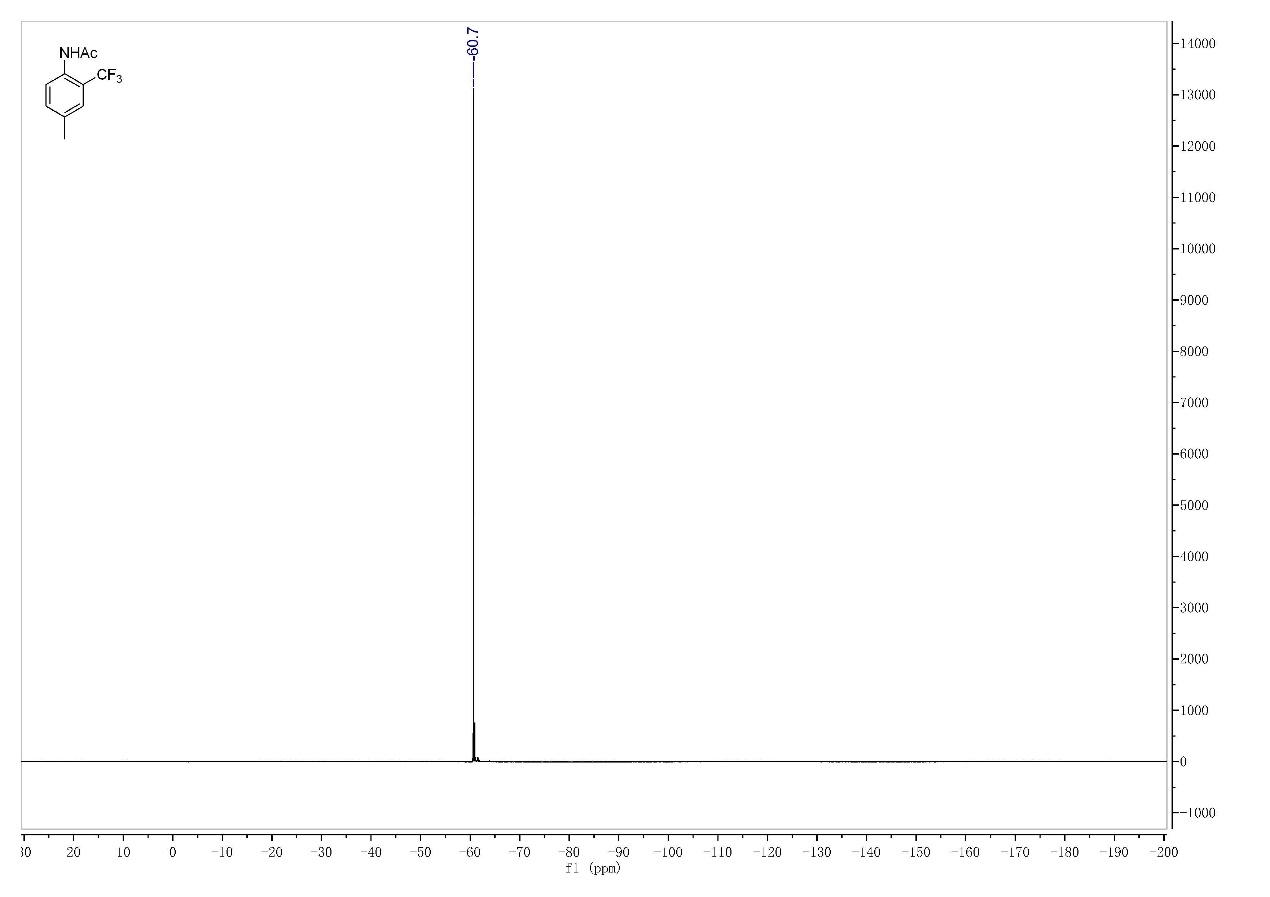

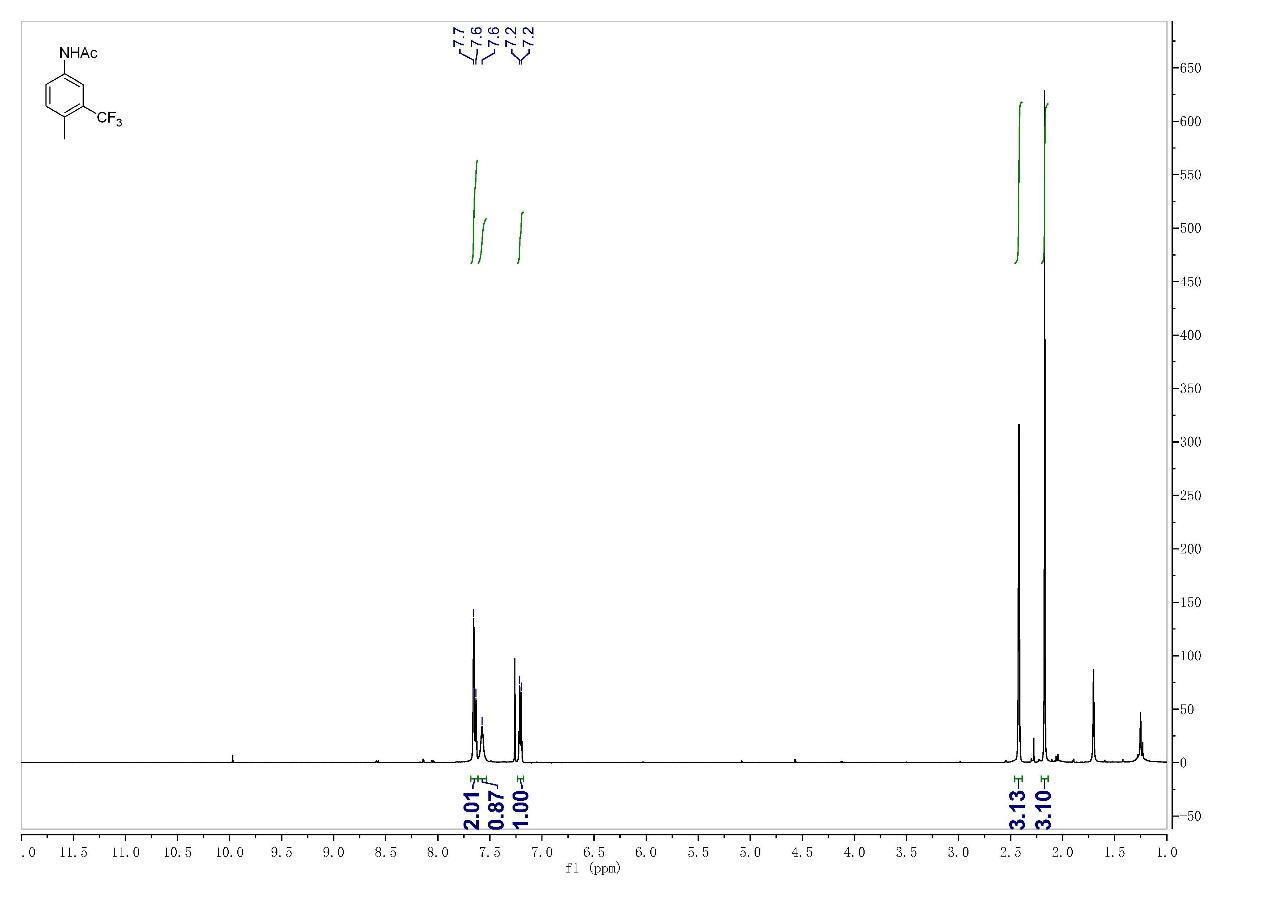

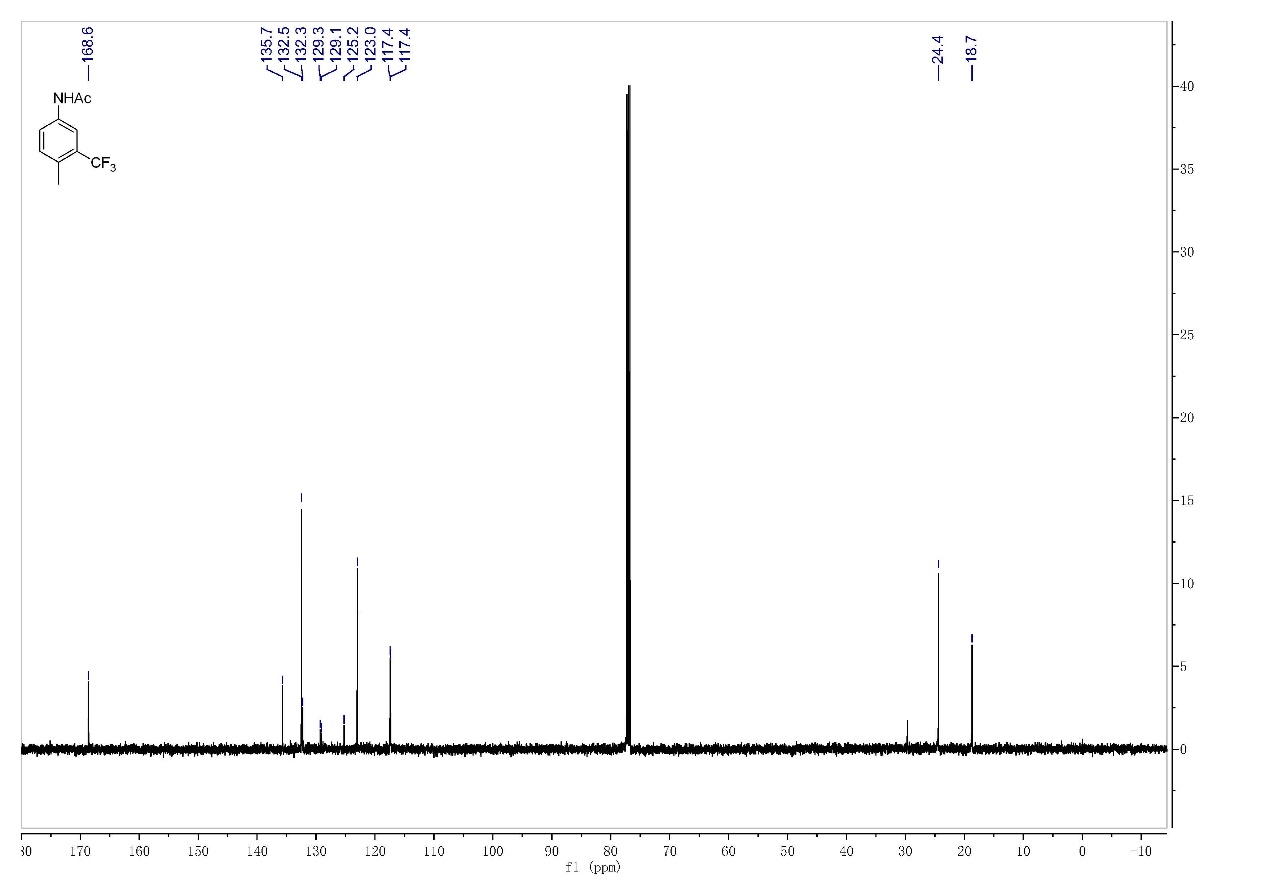

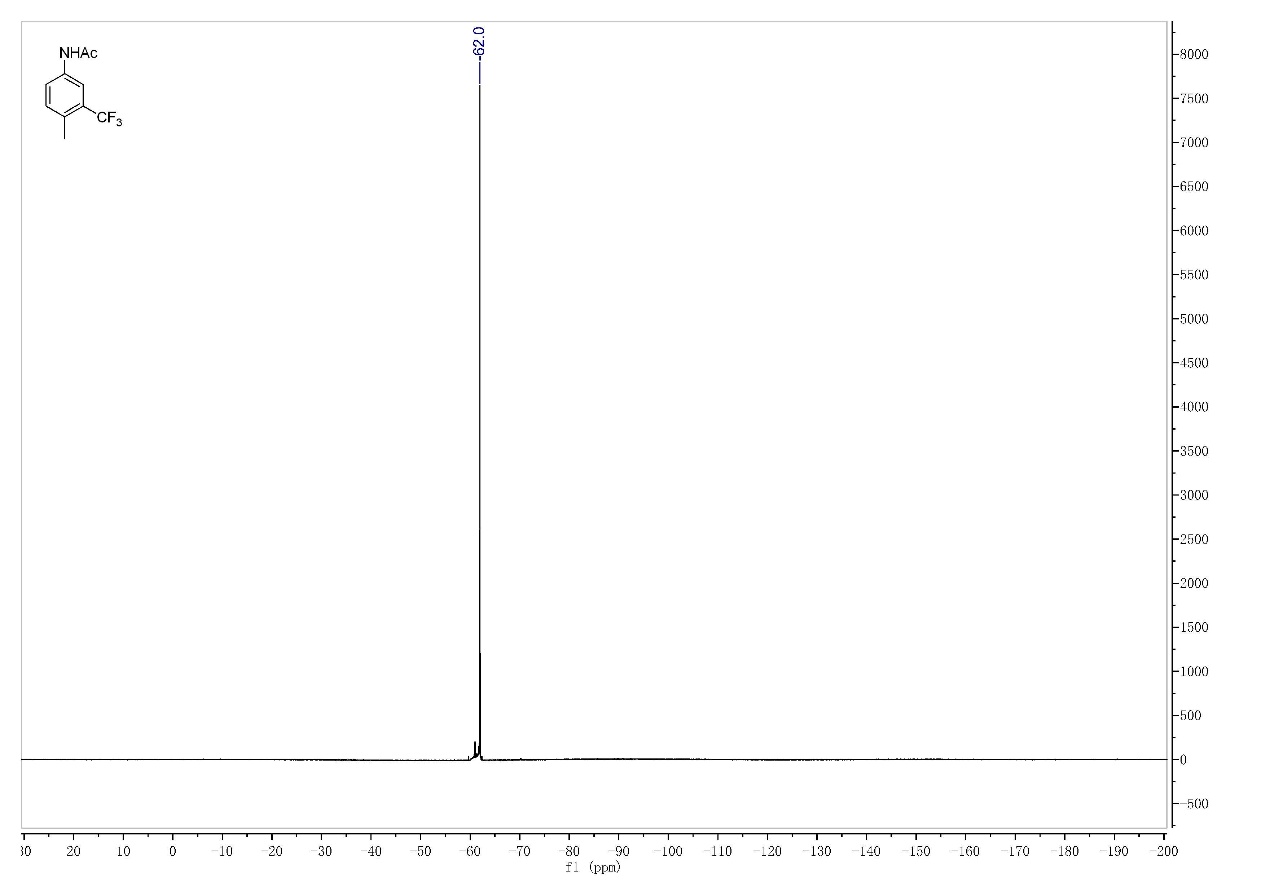

Supplement: Supplementary file 1 [file Data_Sheet_1.docx]
